# Short term association between ozone and mortality: global two stage time series study in 406 locations in 20 countries

**DOI:** 10.1136/bmj.m108

**Published:** 2020-02-10

**Authors:** Ana M Vicedo-Cabrera, Francesco Sera, Cong Liu, Ben Armstrong, Ai Milojevic, Yuming Guo, Shilu Tong, Eric Lavigne, Jan Kyselý, Aleš Urban, Hans Orru, Ene Indermitte, Mathilde Pascal, Veronika Huber, Alexandra Schneider, Klea Katsouyanni, Evangelia Samoli, Massimo Stafoggia, Matteo Scortichini, Masahiro Hashizume, Yasushi Honda, Chris Fook Sheng Ng, Magali Hurtado-Diaz, Julio Cruz, Susana Silva, Joana Madureira, Noah Scovronick, Rebecca M. Garland, Ho Kim, Aurelio Tobias, Carmen Íñiguez, Bertil Forsberg, Christofer Åström, Martina S Ragettli, Martin Röösli, Yue-Liang Leon Guo, Bing-Yu Chen, Antonella Zanobetti, Joel Schwartz, Michelle L Bell, Haidong Kan, Antonio Gasparrini

**Affiliations:** 1 Department of Public Health, Environments and Society, London School of Hygiene and Tropical Medicine, London WC1H 9SH, UK; 2 Institute of Social and Preventive Medicine, University of Bern, Bern, Switzerland; 3 Oeschger Centre for Climate Change Research, University of Bern, Bern, Switzerland; 4 School of Public Health, Key Lab of Public Health Safety of the Ministry of Education and Key Lab of Health Technology Assessment of the Ministry of Health, Fudan University, Shanghai, China; 5 Department of Epidemiology and Preventive Medicine, School of Public Health and Preventive Medicine, Monash University, Melbourne, Australia; 6 Shanghai Children’s Medical Centre, Shanghai Jiao Tong University School of Medicine, Shanghai, China; 7 School of Public Health, Institute of Environment and Population Health, Anhui Medical University, Hefei, China; 8 School of Public Health and Social Work, Queensland University of Technology, Brisbane, Australia; 9 Air Health Science Division, Health Canada, Ottawa, Canada; 10 School of Epidemiology and Public Health, University of Ottawa, Ottawa, Canada; 11 Institute of Atmospheric Physics, Czech Academy of Sciences, Prague, Czech Republic; 12 Faculty of Environmental Sciences, Czech University of Life Sciences, Prague, Czech Republic; 13 Institute of Family Medicine and Public Health, University of Tartu, Tartu, Estonia; 14 Santé Publique France, French National Public Health Agency, Saint Maurice, France; 15 Department of Physical, Chemical and Natural Systems, Universidad Pablo de Olavide, Sevilla, Spain; 16 Potsdam Institute for Climate Impact Research, Potsdam, Germany; 17 Institute of Epidemiology, Helmholtz Zentrum München–German Research Center for Environmental Health, Neuherberg, Germany; 18 Department of Hygiene, Epidemiology and Medical Statistics, National and Kapodistrian University of Athens, Greece; 19 School of Population Health and Environmental Sciences, King’s College London, London, UK; 20 Department of Epidemiology, Lazio Regional Health Service/ASL Roma 1, Rome, Italy; 21 Department of Global Health Policy, School of International Health, Graduate School of Medicine, University of Tokyo, Tokyo, Japan; 22 Faculty of Health and Sport Sciences, University of Tsukuba, Tsukuba, Japan; 23 School of Tropical Medicine and Global Health, Nagasaki University, Nagasaki, Japan; 24 Department of Environmental Health, National Institute of Public Health, Cuernavaca Morelos, Mexico; 25 Department of Epidemiology, Instituto Nacional de Saúde Dr Ricardo Jorge, Lisbon, Portugal; 26 EPIUnit–Instituto de Saúde Pública, Universidade do Porto, Porto, Portugal; 27 Department of Environmental Health, Instituto Nacional de Saúde Dr Ricardo Jorge, Porto, Portugal; 28 Department of Environmental Health, Rollins School of Public Health, Emory University, Atlanta, USA; 29 Natural Resources and the Environment Unit, Council for Scientific and Industrial Research, Pretoria 0001, South Africa; 30 Unit for Environmental Sciences and Management, North-West University, Potchefstroom, South Africa; 31 Department of Geography, Geo-informatics and Meteorology, University of Pretoria, Pretoria, South Africa; 32 Graduate School of Public Health and Institute of Health and Environment, Seoul National University, Seoul, Republic of Korea; 33 Institute of Environmental Assessment and Water Research, Spanish Council for Scientific Research, Barcelona, Spain; 34 Department of Statistics and Computational Research, University of Valencia, Valencia, Spain; 35 Spanish Consortium for Research on Epidemiology and Public Health (CIBERESP), Madrid, Spain; 36 Department of Public Health and Clinical Medicine, Umeå University, Umeå, Sweden; 37 Swiss Tropical and Public Health Institute, Basel, Switzerland; 38 University of Basel, Basel, Switzerland; 39 Environmental and Occupational Medicine, National Taiwan University and NTU Hospital, Taiwan; 40 Department of Environmental Health, Harvard T.H. Chan School of Public Health, Boston, MA, USA; 41 School of Forestry and Environmental Studies, Yale University, New Haven, CT, USA; 42 Shanghai Key Laboratory of Atmospheric Particle Pollution and Prevention (LAP3), Fudan University, Shanghai, China; 43 Centre for Statistical Methodology, London School of Hygiene and Tropical Medicine, London, UK; 44 Centre on Climate Change and Planetary Health, London School of Hygiene and Tropical Medicine, London, UK

## Abstract

**Objective:**

To assess short term mortality risks and excess mortality associated with exposure to ozone in several cities worldwide.

**Design:**

Two stage time series analysis.

**Setting:**

406 cities in 20 countries, with overlapping periods between 1985 and 2015, collected from the database of Multi-City Multi-Country Collaborative Research Network.

**Population:**

Deaths for all causes or for external causes only registered in each city within the study period**.**

**Main outcome measures:**

Daily total mortality (all or non-external causes only).

**Results:**

A total of 45 165 171 deaths were analysed in the 406 cities. On average, a 10 µg/m^3^ increase in ozone during the current and previous day was associated with an overall relative risk of mortality of 1.0018 (95% confidence interval 1.0012 to 1.0024). Some heterogeneity was found across countries, with estimates ranging from greater than 1.0020 in the United Kingdom, South Africa, Estonia, and Canada to less than 1.0008 in Mexico and Spain. Short term excess mortality in association with exposure to ozone higher than maximum background levels (70 µg/m^3^) was 0.26% (95% confidence interval 0.24% to 0.28%), corresponding to 8203 annual excess deaths (95% confidence interval 3525 to 12 840) across the 406 cities studied. The excess remained at 0.20% (0.18% to 0.22%) when restricting to days above the WHO guideline (100 µg/m^3^), corresponding to 6262 annual excess deaths (1413 to 11 065). Above more lenient thresholds for air quality standards in Europe, America, and China, excess mortality was 0.14%, 0.09%, and 0.05%, respectively.

**Conclusions:**

Results suggest that ozone related mortality could be potentially reduced under stricter air quality standards. These findings have relevance for the implementation of efficient clean air interventions and mitigation strategies designed within national and international climate policies.

## Introduction

Ground level ozone is a highly reactive, oxidative gas commonly found in urban and suburban environments, mostly derived from anthropogenic emissions. Numerous epidemiological studies and several reviews from health and environmental agencies worldwide have reported that exposure to this pollutant is associated with adverse health outcomes, including increased short term mortality and morbidity.^1^ ^2^ ^3^ ^4^ Evidence on the health impacts related to ozone exposure has important implications in climate change research, as ozone levels are predicted to increase with global warming.^5^

Short term ozone-mortality associations have been widely assessed in several multi-location time series studies in Europe, the United States, Canada, Latin America, and Asia.^2^ ^6^ ^7^ ^8^ The general methodological framework consists of pooling location specific estimated risks, accounting for potential heterogeneity in the magnitude of the effect and uncertainty. In addition, the increased statistical power of multi-location analyses allows for the exploration of potentially complex features of the association (ie, non-linearity, delayed effects and harvesting, or differential risks by season).^9^ ^10^ ^11^ However, previous multi-location studies included a small number of cities and countries, were generally of limited geographical scope, and applied heterogeneous analytical approaches and modelling choices, making it difficult to draw consistent and comprehensive conclusions across different regions of the world.

Although ozone-mortality associations have been widely assessed, results are rarely reported in terms of health impacts, such as excess deaths.^12^ Available figures are mostly derived from long term exposure metrics and risks estimated in specific subgroups, which are usually extrapolated to the general population.^13^ ^14^ Quantification of health burdens from air pollution can be extremely useful for the design of efficient public health interventions, including the definition, assessment, and review of air quality standards. Current air quality standards vary greatly between countries, and only a few of them meet the stricter World Health Organization recommendation.^15^ Comparting the effects on health of ozone levels above different air quality standards can provide valuable insights into potential public health benefits achieved by strengthening current clean air policies. Although a few studies have attempted to tackle this problem, a widespread evaluation across several countries, which would help to identify more affected areas with a greater need for intervention, is still lacking.^16^ ^17^

We carried out a multi-location time series analysis of mortality associated with short term exposure to ozone using data from 406 cities in 20 countries from multiple geographical regions. Next, we explored potential complexities of the association—namely, non-linearity, mortality displacement, and seasonality. Finally, we quantified the impacts on ozone associated mortality of specific concentration ranges consistent with the current air quality standards levels and then compared these estimates across countries.

## Methods

### Data collection

We initially extracted data for 434 locations across the 20 countries from the database of the Multi-city Multi-country (MCC) Collaborative Research Network (http://mccstudy.lshtm.ac.uk/) available at the time of the study. These include location specific daily mortality counts and environmental measures (weather and air pollutants) in largely overlapping periods from 1 January 1985 to 31 December 2015. For each location we derived daily time series of ozone (maximum eight hour average), particulate matter with an aerodynamic diameter less than or equal to 10 µm (PM_10_, per µg/m^3^, 24 hour average), particulate matter with an aerodynamic diameter less than or equal to 2.5 µm (PM_2.5_, per µg/m^3^, 24 hour average), nitrogen dioxide (24 hour average), total mortality, mean temperature (°C), and relative humidity (%). Mortality was represented by all cause deaths in Canada, the Czech Republic, Estonia, France, Germany, Greece, Italy, Japan, Mexico, Portugal, South Africa, South Korea, Sweden, Taiwan, UK, and US, whereas deaths due to non-external causes (eg, excluding self-intentional harm, poisoning) were used in Australia, China, and Spain, and non-external causes other than unintentional injuries in Switzerland (see supplementary eMethods 1 for the specific international classification of diseases codes used in each country). City specific air pollution series were derived from daily measurements of one or more monitors of the national or regional network. When more than one monitor was available, we computed the daily level of each pollutant (24 hour average or eight hour maximum) as the average across monitors of the city, consistent with previous multi-city studies.^2^ We excluded 28 cities as a result of poor quality data or limited periods (less than three years), with 406 locations included in the final analysis (see supplementary eMethods 1 for a detailed description of the data, exposure assessment, and exclusion criteria).

### Statistical analysis

The general statistical framework applied here is an extension of the classic two stage design^6^ and incorporates complex multivariable associations, hierarchical pooling methods, and the computation of impact measures.^18^ ^19^ ^20^ Briefly, we first estimated city specific ozone-mortality risks from separate time series regression models and then pooled these through a meta-analysis in the second stage. In a final step, we derived impact estimates, expressed as excess mortality fractions associated with ozone, from the pooled country specific risks and city specific exposure series. Using this general statistical framework, we performed a set of additional and sensitivity analyses to investigate specific features of the association. The analyses were conducted with R software (version 3.5.2) using the dlnm and mixmeta packages.

#### Main analysis

In the first stage, we performed city specific time series analyses using generalised linear models with quasi-Poisson family. In this type of regression model, to properly scale the standard deviation of the coefficients proportionally to the potential overdispersion, a quasi-likelihood is applied. This phenomenon is common in these types of data, when the variability is larger than that expected under the assumption of a Poisson distribution. We assessed short term ozone-mortality associations using unconstrained distributed lag linear models.^11^ ^21^ These models account for delayed effects of time varying exposures and quantify net effects over a predefined lag period.^20^ For the main model, we selected lag 0-1, estimating cumulative associations with the same and previous day’s exposures. The regression model included a natural spline of time with seven degrees of freedom each year, selected based on a quasi-likelihood version of the Akaike information criterion for 4, 6, 7, 8, and 10 degrees of freedom, and indicator variables for the day of the week, to control for long term, seasonal, and weekly variations in risk. Unlike in most previous studies on ozone, we applied a stricter control for temperature by using distributed lag non-linear models, an extension of distributed lag linear models for modelling complex non-linear and lagged associations. Following modelling choices applied in published analyses, we modelled the net temperature-mortality association over lag 0-21 (see supplementary eMethods 2).^22^

In the second stage we pooled city specific estimates through a multilevel meta-analysis. This novel meta-analytical model defines more complex random effects that can account for variations in risk across two nested grouping levels, represented by cities within countries.^19^ This approach allowed the derivation of improved estimates of ozone-mortality associations at both city and country level, defined as best linear unbiased predictions. Best linear unbiased predictions borrow information across units within the same hierarchical level and can provide more accurate estimates, especially in locations with small daily mortality counts or short series. We tested the presence of heterogeneity and reported it using multilevel extensions of Cochran Q test and I^2^ statistic.^23^ Association estimates, expressed as relative risk of mortality per 10 µg/m^3^ increase of ozone and 95% confidence interval were derived for each country from the corresponding best linear unbiased predictions.

Risk estimates for ozone related mortality were then translated into impact measures, represented by excess mortality, following a method described elsewhere.^18^ Briefly, for each city we computed the daily number of deaths attributable to ozone (or daily excess deaths) using the corresponding risk estimate associated with the level of ozone in each day. Regarding the latter, we used country specific best linear unbiased predictions instead of the city specific estimates to avoid imbalances due to selection of cities and periods within each country. City specific estimates were reported as annual average number of excess deaths and 95% confidence intervals, so allowing for a proper comparison between locations with different lengths of study period. Then, country specific impacts were represented by excess mortality fractions (%) computed as the sum of the city specific daily excess deaths divided by the total mortality for each country. We used fractions instead of number of excess deaths, as excess deaths are not comparable across countries given the dependency on the denominator (ie, total mortality), which at the same time depends on the number of locations included. Although no evidence of a “safe” threshold exists, we computed associated deaths only for days with ozone levels above 70 µg/m^3^, as in previous health impact assessments.^4^ We considered this counterfactual scenario of 70 µg/m^3^ because ozone levels below this threshold could be mostly attributed to non-anthropogenic sources. A counterfactual scenario defined at 0 µg/m^3^ would not be appropriate either as it is not realistic given the ubiquitous presence of low levels of ozone derived from natural sources. We also disaggregated mortality impacts into contributions for exposure ranges above and between current air quality standards: 100 µg/m^3^ (WHO), 120 µg/m^3^ (European Union directive), 140 µg/m^3^ (National Ambient Air Quality Standard (NAAQS) in the US, about 0.070 parts per million), and 160 µg/m^3^ (Chinese Ambient Air Quality Standard (CAAQS) level 2).^15^

#### Additional complexities and sensitivity analyses

We performed a series of additional subanalyses to explore more complex features of the association, such as potential non-linearity, lagged effects, and seasonal differences. Firstly, we modelled exposure-response functions with a non-linear function consisting of a cubic B spline with internal knots at 50 µg/m^3^ and 60 µg/m^3^ of ozone. Secondly, we assessed delayed risks and potential mortality displacement by extending the lag dimension of the distributed lag linear model up to 30 days. Lag-response associations were modelled using a natural cubic spline with three internal knots placed at equally spaced lag values in the log scale. Thirdly, we assessed seasonal differences through interaction models between an indicator of season and the distributed lag linear model of ozone, as described elsewhere.^24^ We derived the ozone-mortality risk for the warm season (June to August in the northern hemisphere, December, January, and February in the southern hemisphere) and cold seasons (the remaining months).

Modelling choices in the main model and extensions previously described were assessed and compared through the quasi-likelihood version of the Akaike information criterion and multivariate extensions of the Wald test. For sensitivity analyses, we first assessed changes in control for time trends and the potential confounding from other air pollutants (PM_10,_ PM_2.5_, and nitrogen dioxide) and relative humidity by including each of these terms separately in the model. We then assessed the exclusion of a subset of US cities with data for summer only, which were included in the main analysis, and then different modelling approaches to control for temperature. See supplementary eMethods 1 and 2 for a description of the modelling details.

### Patient and public involvement

This was a multinational collaboration using aggregated city level mortality and environmental data. Patients and members of the public did not contribute to the steering committee, design, or other areas of the study, which involved complex research methods and analysis. 

## Results

**Fig 1:**
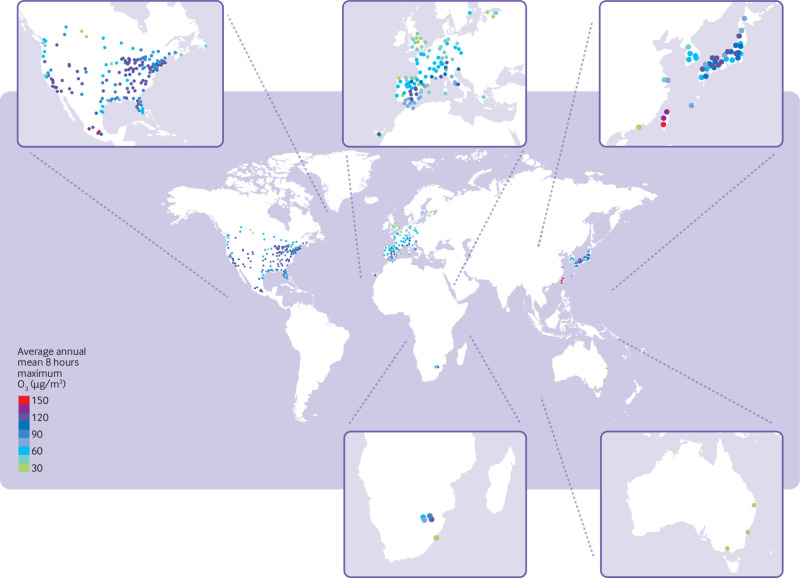
Geographical distribution of city specific average annual means of ozone (O_3_, maximum eight hour average) of 406 cities of the Multi-City Multi-Country Collaborative Research Network included in the study

Table 1 provides a summary description of the data included for each country. A total of 45 165 171 deaths were analysed in the 406 cities, with an average time series of 13 years. Average annual mean ozone levels were widely heterogeneous across cities both between and within countries (fig 1). For example, lower levels were registered in Australian and northern European cities, whereas higher annual averages were found in some cities in the central area of the US, in Mexico, and in Taiwan. Supplementary eTable1 provides country specific descriptive summaries of the other air pollutants and humidity and eTable 2 reports the corresponding city specific descriptive results.

**Table 1:** Environmental and mortality data

Countries	No of cities	Period	No of deaths*	Median (interquartile range) No of daily deaths	Median (interquartile range) ozone level (µg/m^3^)†	Median (interquartile range) mean temperature (°C)
Australia	3	2000-19	513 527	49.3 (43.7-55.7)	31.2 (24.2-38.6)	18.3 (14.8-21.5)
Canada	26	1986-2011	2 914 630	12.8 (10.5-15.3)	69.2 (53.9-88.4)	7.3 (−1.0-15.7)
China	3	1996-2015	780 655	87.3 (71.7-140.3)	49.3 (27.8-77.5)	20.4 (13.0-25.7)
Czech Republic	1	1994-2009	214 062	36.0 (32.0-41.0)	69.3 (47.4-95.0)	9.2 (2.5-15.3)
Estonia	4	2002-15	80 043	5.0 (3.5-6.5)	48.9 (36.7-61.8)	6.0 (0.2-13.6)
France	18	2000-10	1 197 555	16.3 (13.7-19.1)	67.8 (46.8-87.4)	12.7 (7.6-17.9)
Germany	12	1993-2015	3 099 176	30.4 (26.4-34.8)	57.1 (35.8-79.2)	10.5 (4.8-15.9)
Greece	1	2001-10	287 969	78.0 (70.0-87.0)	75.1 (52.8-97.5)	17.9 (12.9-24.9)
Italy	9	2006-15	373 421	15.1 (12.6-17.9)	74.1 (50.5-97.0)	15.8 (10.2-22.1)
Japan	45	2011-15	1 856 232	22.3 (19.1-25.7)	78.5 (62.4-98.4)	16.1 (7.5-22.7)
Mexico	7	2000-12	2 018 313	61.0 (53.7-69.4)	108.9 (85.1-135)	18.6 (15.9-20.5)
Portugal	2	1997-2012	536 958	47.0 (41.0-54.0)	64.2 (50.2-79.2)	16.1 (12.5-19.6)
South Africa	5	2004-13	924 478	58.4 (48.8-67.0)	69.5 (52.9-89.5)	18.3 (14.2-21.2)
South Korea	7	1999-2015	1 662 199	38.3 (34.0-42.7)	59.5 (42.7-81.9)	15.1 (5.8-22.1)
Spain	48	2004-14	1 294 162	6.7 (5.1-8.4)	70.0 (53.9-84.7)	15.3 (10.3-21.1)
Sweden	1	1990-2010	201 197	26.0 (22.0-30.0)	61.9 (48.9-76.0)	6.8 (1.2-13.9)
Switzerland	8	1995-2013	230 587	4.2 (2.9-5.6)	72.8 (47.0-98.1)	10.7 (4.4-16.5)
Taiwan	3	2008-14	443 680	57.0 (51.0-63.7)	109.1 (82.1-138.6)	24.8 (20-28.2)
UK	15	1993-2006	2 073 285	28.4 (24.5-32.9)	51.6 (36.7-65.2)	10.4 (6.5-14.6)
USA	188	1985-2006	24 463 042	16.3 (13.6-19.3)	80.1 (58.9-104.0)	14.9 (7.5-21.9)

* Deaths due to non-external causes (Australia, China, Spain, Switzerland (including unintentional injuries)) or to all cause mortality (remaining countries). See supplementary eMethods 1 for a description of the data. Country specific summaries of other air pollutants and relative humidity are provided in supplementary eTable 1 and city specific descriptive summaries are reported in supplementary eTable 2.

† Daily maximum eight hour mean.

**Fig 2:**
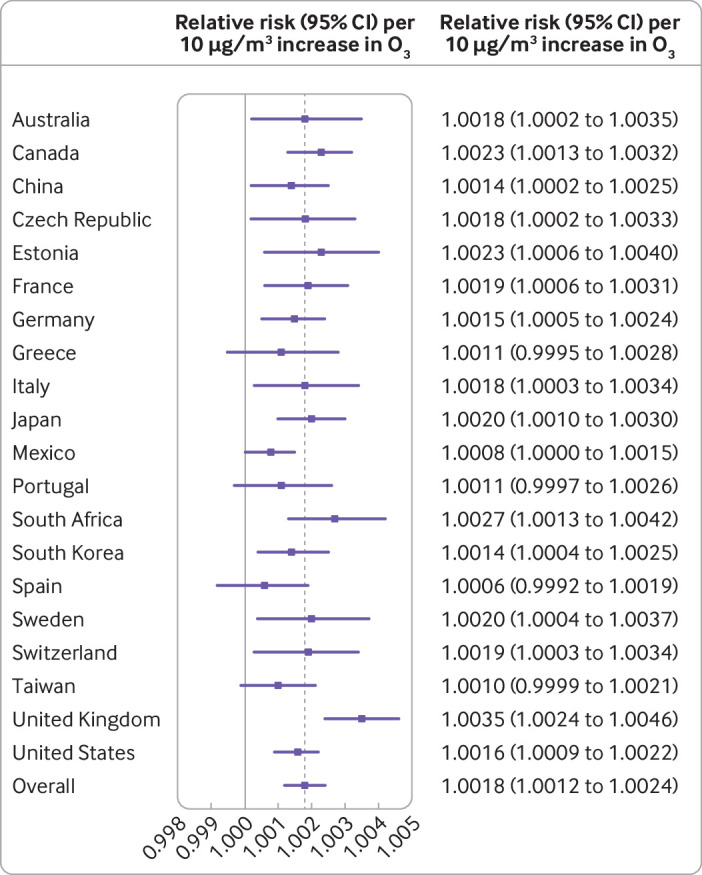
Overall and country specific short term ozone-mortality association, expressed as relative risk per 10 µg/m^3^ increase in ozone (O_3_, maximum eight hour average) (lag 01)

On average, each 10 µg/m^3^ increase in ozone was associated with an overall relative risk of mortality of 1.0018 (95% confidence interval 1.0012 to 1.0024) (fig 2). Some heterogeneity was found across country and city specific risks (I^2^=29.8%, Cochran Q P<0.001). Larger risk estimates were found in the UK (1.0035 (1.0024 to 1.0046)), South Africa (1.0027 (1.0013 to 1.0042)), Estonia (1.0023 (1.0006 to 1.0040)), and Canada (1.0023 (1.0013 to 1.0032)), whereas Australia, China, the Czech Republic, France, Germany, Italy, Japan, South Korea, Sweden, Switzerland, and the US showed similar risks, ranging between 1.0014 and 1.0020. Lower and imprecise associations were estimated for Greece (1.0011 (0.9995 to 1.0028)), Mexico (1.0008 (1.000 to 1.0015)), Portugal (1.0011 (0.9997 to 1.0026)), Spain (1.0006 (0.9992 to 1.0019)), and Taiwan (1.0010 (0.9999 to 1.0021)). Supplementary eFigure 1 provides the corresponding figures with the relative risks for an increase in 10 parts per billion of ozone.

**Fig 3:**
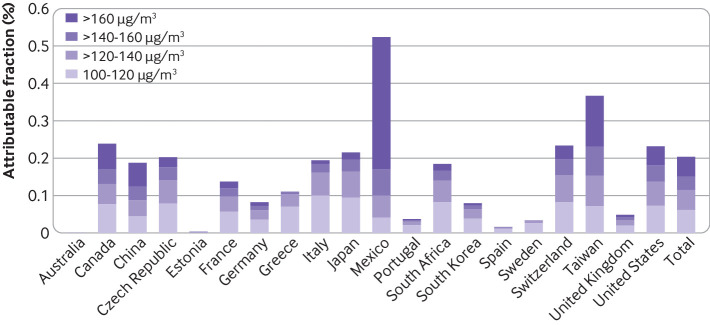
Overall and country specific excess mortality (%) associated with ozone by specific ranges defined between thresholds consistent with current air quality standards. (No excess mortality associated with ozone was found in Australia, as daily ozone levels were below the maximum background level of 70 µg/m^3^). 100 µg/m^3^, World Health Organization guideline; 120 µg/m^3^, European Union directive; 140 µg/m^3^ (about 0.070 parts per million); National Ambient Air Quality Standard (NAAQS) in the US; 160 µg/m^3^ Chinese Ambient Air Quality Standard (CAAQS)

Figure 3 depicts the excess mortality fractions above the WHO guideline and their distribution across intervals between the other air quality standards for each country, whereas supplementary eTable 3 and eTable 4 report the corresponding figures for excess fractions for total ozone (>70 µg/m^3^) and above and between air quality standards. Table 2 shows fractions and annual number of excess deaths associated with ozone for the total range of exposure and above the WHO guideline for a selection of the main cities in each country and overall across the 406 locations (supplementary eTable5 shows the estimates for all cities). Total mortality associated with ozone greater than 70 µg/m^3^ accounted for 0.26% of deaths (95% confidence interval 0.24% to 0.28%), which translates into 8203 annual excess deaths (95% confidence interval 3525 to 12 840) across the 406 locations studied (table 2). A substantial residual excess mortality of 0.20% (95% confidence interval 0.18% to 0.22%) corresponding to 6262 (95% confidence interval 1413 to 11 065) annual excess deaths remained when restricting to days with levels above the WHO guideline of 100 µg/m^3^. This proportion varied greatly by country, with considerably larger fractions in Mexico (0.52% (0.14% to 0.92%)) and Taiwan (0.37% (0.08% to 0.64%)) (fig 3, supplementary eTable 3). A mortality excess around 0.20% was estimated in Canada, China, Italy, Japan, South Africa, Switzerland, and the US, whereas France, Germany, South Korea, and the UK reported smaller percentages, ranging between 0.14% and 0.05% (fig 3, supplementary eTable 3). Imprecise or almost null estimates were found in the Czech Republic, Estonia, Greece, Portugal, Spain, and Sweden (supplementary eTable 3). Overall mortality fractions above more lenient air quality standards (ie, the European Union, NAAQS, and CAAQS) decreased progressively to 0.14%, 0.09%, and 0.05%, respectively (supplementary eTable 3). Only Mexico reported a considerably higher fraction, of 0.35% above the highest air quality standards of 160 µg/m^3^, although this finding was highly uncertain (black bar in fig 3, supplementary eTable 3). Null excess deaths were found in Australia, as daily exposure levels were all below 70 µg/m^3^. A similar pattern was found across estimates for the main cities in each country (table 2). A substantial number of annual excess deaths were associated with ozone levels above the WHO guideline—namely, 694 (95% confidence interval 22 to 1317) in the Valley of Mexico, 211 (112 to 307) in Los Angeles, 170 (40 to 304) in Tokyo, 128 (59 to 197) in Toronto, 82 (19 to 148) in Johannesburg, 48 (0 to 96) in Paris, and 37 (15 to 57) in London (table 2). Supplementary eTable 5 shows the corresponding estimates for the 406 cities.

**Table 2:** Excess mortality associated with ozone for total (>70 µg/m^3^) and above World Health Organization guideline of 100 µg/m^3^ in main cities of each participating country and overall estimates for the 406 cities

Countries	Cities	Total (>70 µg/m^3^)*			Above WHO guideline (100 µg/m^3^)	
		% Excess fraction (95% CI)	No of annual excess deaths (95% CI)		% Excess fraction (95% CI)	No of annual excess deaths (95% CI)
Australia†	Sydney	0 (0 to 0)	0 (0 to 0)		0 (0 to 0)	0 (0 to 0)
Canada	Toronto	0.59 (0.34 to 0.85)	159 (90 to 228)		0.48 (0.22 to 0.73)	128 (59 to 197)
China	Shanghai	0.32 (0.04 to 0.57)	117 (15 to 209)		0.27 (−0.01 to 0.53)	99 (−4 to 195)
Czech Republic	Prague	0.27 (0.02 to 0.48)	38 (3 to 69)		0.20 (−0.06 to 0.44)	29 (−9 to 63)
Estonia	Tallinn	0.01 (0.00 to 0.02)	1 (0 to 1)		0.00 (−0.01 to 0.01)	0 (−1 to 1)
France	Paris	0.15 (0.05 to 0.26)	70 (24 to 119)		0.11 (0.00 to 0.21)	48 (0 to 96)
Germany	Berlin	0.12 (0.04 to 0.20)	46 (14 to 74)		0.08 (−0.01 to 0.17)	30 (−3 to 62)
Greece	Athens	0.16 (−0.07 to 0.41)	52 (−23 to 132)		0.11 (−0.13 to 0.37)	35 (−42 to 117)
Italy	Rome	0.27 (0.05 to 0.52)	69 (13 to 132)		0.19 (−0.05 to 0.44)	48 (−12 to 111)
Japan	Tokyo	0.27 (0.14 to 0.40)	249 (127 to 371)		0.18 (0.04 to 0.32)	170 (40 to 304)
Mexico	Valley of Mexico	0.73 (0.04 to 1.38)	707 (39 to 1,339)		0.72 (0.02 to 1.36)	694 (22 to 1,317)
Portugal	Lisbon	0.09 (−0.03 to 0.2)	20 (−6 to 45)		0.04 (−0.09 to 0.17)	9 (−20 to 39)
South Africa	City of Johannesburg	0.32 (0.15 to 0.49)	121 (59 to 187)		0.22 (0.05 to 0.39)	82 (19 to 148)
South Korea	Seoul	0.10 (0.03 to 0.17)	41 (13 to 71)		0.06 (−0.01 to 0.14)	27 (−3 to 58)
Spain	Madrid	0.03 (−0.04 to 0.11)	9 (−12 to 31)		0.01 (−0.07 to 0.10)	3 (−21 to 27)
Sweden	Stockholm	0.10 (0.02 to 0.18)	10 (2 to 18)		0.03 (−0.07 to 0.13)	3 (−7 to 13)
Switzerland	Zurich	0.31 (0.05 to 0.54)	13 (2 to 22)		0.23 (−0.02 to 0.48)	10 (−1 to 20)
Taiwan	Taipei	0.34 (−0.05 to 0.72)	131 (−21 to 276)		0.28 (−0.11 to 0.67)	109 (−43 to 258)
UK	London	0.10 (0.07 to 0.12)	63 (44 to 81)		0.06 (0.02 to 0.09)	37 (15 to 57)
USA	Los Angeles	0.41 (0.24 to 0.57)	242 (142 to 335)		0.36 (0.19 to 0.52)	211 (112 to 307)
20 MCC countries‡	406 MCC cities	0.26 (0.24 to 0.28)	8,203 (3,525 to 12 840)		0.20 (0.18 to 0.22)	6,262 (1,413 to 11 065)

* Total refers to ozone related deaths when levels above 70 µg/m^3^ (defined as maximum background levels).

† No excess mortality associated with ozone were found in Australia, as daily ozone levels were below the maximum background level set up at 70 µg/m^3^.

‡ Countries contributing to the Multi-City Multi-Country (MCC) Collaborative Research Network included in the present study.

Additional analyses suggested no evidence of non-linearity in the concentration-response association (according to the quasi-likelihood version of the Akaike information criterion) (supplementary eFigure 2). The assessment of the lagged associations confirmed an immediate ozone-mortality association during the first week. However, lag specific estimates below 1 were found after the second week, which resulted in a slightly lower overall cumulative association of 1.0015 (95% confidence interval 0.9991 to 1.0032) when considering the delayed effects over the first 30 days after the exposure. Finally, no evidence of seasonal differences in ozone-mortality association were found (warm season: 1.0012 (95% confidence interval 1.000 to 1.0026); cold season: 1.0015% (1.0006 to 1.0024), Wald test P=0.37).

Results from sensitivity analyses suggest that risk estimates of the main analysis were robust to the different modelling choices related to the control for time trends and adjustment by the three air pollutants and humidity (supplementary eTable 6). However, ozone-mortality risk estimates seemed to be sensitive to the approach to control for temperature (supplementary eFigure 3). We found larger ozone-mortality association estimates using less stringent control, although quasi-likelihood Akaike information criterion values suggested that the model with distributed lag non-linear model of temperature (main model) provided the best fit.

## Discussion

On average, this study found an overall short term ozone-mortality association of 1.0018 (95% confidence interval 1.0012 to 1.0024) per 10 µg/m^3^ increase in ozone. This evidence is supported by previous epidemiological and experimental studies suggesting several pathophysiological mechanisms (e.g. systemic inflammation, haemostatic alterations).^25^ ^26^ Larger associations were found in previous multi-country studies, including a subset of countries investigated here (eg, relative risk of 1.0022 in APHEA (Air Pollution and Health: A European Approach), 1.0026 in APHENA (Air Pollution and Health: A Combined European and North American Approach), per 10 µg/m^3^ increase),^11^ ^21^ or single country studies (eg, relative risk of 1.0025 in the US (originally 1.0052 per 10 parts per billion increase), and China 0.55% per 10 µg/m^3^ increase, and 1.015 in Italy).^27^ ^28^ ^29^ Differences in the definition of the exposure variable (eg, moving average, single lag) and modelling approach could explain these discrepancies in the magnitude of the association. For example, compared with previous studies, we applied a stronger control for temperature (ie, distributed lag non-linear models), fully accounting for non-linearity and lagged temperature-mortality associations.^22^ In fact, results from sensitivity analyses are consistent with previous findings showing that ozone-mortality risk estimates were sensitive to the modelling strategy to control for temperature, reporting larger risks when using simpler approaches (supplementary eFigure 3).^27^ Moreover, one of the novelties of the applied statistical framework is the use of multilevel meta-analytical models in the second stage, properly accounting for heterogeneity across cities and countries.

Our results showed important differences in the ozone-mortality association across countries. For example, while some areas such as UK, South Africa, Canada, and Estonia reported the largest risk estimates above 1.0020, smaller or imprecise estimates below 1.0011 were found in Greece, Mexico, Spain, and Taiwan. This unclear pattern would suggest that although several community level factors have been proposed as potential modifiers in single country studies (eg, population characteristics), these might not fully characterise differences between countries.^30^ Future multi-country studies are needed to provide further evidence on the factors defining the level of vulnerability of a population to air pollution.

This study also provides evidence on the potential public health benefits of stricter clean air policies. In particular, we found that 0.20% excess mortality, which translates into more than 6000 deaths each year, related to short term exposure to ozone could have been avoided if ambient levels were below the WHO guideline of 100 µg/m^3^ in the 406 cities included in the study. Recent reviews found that most of the current air quality standards do not comply with the WHO air quality guideline,^15^ and that 80% of the world’s population in urban areas are exposed to air pollution levels above this threshold.^31^ Moreover, an additional 0.06% of excess deaths is associated with ozone levels between 70 µg/m^3^ and 100 µg/m^3^. These findings support the WHO initiative of encouraging countries to reconsider current air quality standards and enforce stronger emission restrictions and other public health interventions to meet its recommendations. Additionally, our results have important implications for healthcare practice. Apart from the implementation of clean air policies, individual strategies to reduce personal exposure to air pollutants are also desirable.^32^ In this regard, clinicians play an important role in counselling patients with potentially a higher susceptibility to adverse health outcomes related to air pollution. For example, professionals can advise sensitive individuals to stay indoors or avoid doing exercise during episodes of high ambient ozone.

Previous studies showed that important health benefits could be achieved if reductions of ozone levels are reached.^9^ ^13^ ^16^ However, in this multi-country study we compared excess mortality estimates across air quality standards and countries, providing additional insights on specific areas with more urgent need of further interventions. For example, we found that 0.52% of total mortality in Mexico was associated with ozone above the WHO limit, the largest mortality fraction among the studied countries. This was associated with the highest ozone levels registered in the Mexican cities, especially above the 160 µg/m^3^ limit, which is close to its current air quality standards of 156 µg/m^3^. This means that attaining the current lenient standards would prevent a substantial proportion of ozone related deaths in this country. In contrast, results for the UK show a lower mortality fraction, despite the strongest ozone-mortality association, owing to the lower ozone levels registered in this country.

### Strengths and limitations of this study

This large epidemiological investigation on short term ozone-mortality associations included almost 50 million deaths from 406 cities in 20 countries from different regions across the world. Given its large sample size and wide geographical coverage, we were able to obtain consistent evidence of an association between short term exposure to ozone and total mortality. In addition, we provided ozone related impact estimates, quantified as excess mortality, across different air quality standards, countries and cities, providing evidence with important public health implications.

We were able to explore additional complexities of the association by taking advantage of the large statistical power and advanced statistical techniques. Firstly, our results support the conclusions of previous studies on a generally linear concentration-response functions, with no indication of threshold.^9^ ^27^ Secondly, we found evidence of a potential mortality displacement in the third and fourth week after the exposure. A similar lag pattern has been previously observed.^10^ ^11^ However, potential mechanisms explaining this delayed and sustained pattern remain unclear. Finally, we found no evidence of seasonal differences in the ozone-mortality association. Previous multi-site studies have provided conflicting results, with larger risks in cold seasons in Asia^27^ and in warm seasons in the US and Europe.^6^ Further analyses are warranted to characterise different patterns across regions.

This study has some limitations. Firstly, our results should not be considered truly global estimates, because several areas of the world such as South America, Africa, and the Middle East are unrepresented or were not assessed. In addition, the reported nationwide results might not be representative of the true impacts for some countries with a limited number of cities included in the study (eg, Sweden, Czech Republic, China). In particular, the estimated number of total excess deaths attributed to ozone should be interpreted as the sum of impacts in the 406 observed locations and not as total estimates across the 20 countries. Although excess fractions could be considered proper representations of the impacts for each country, the total excess number of deaths for each country is highly dependent on the total mortality considered in the study—that is, the number of locations included in each country. Systematic differences could also exist between countries in the characteristics of monitors (type, proximity to study area), study area boundaries, temporal coverage, data processing before data collection, and the collection of mortality data (eg, case ascertainment, codification). However, we ensured that the data fulfilled a minimum set of requirements for quality, a similar definition for the eight hour maximum metric, and location of the monitor (ie, within the study area or close enough to ensure its representativeness). Risks and impact estimates were only reported for total mortality (ie, deaths due to all or non-external causes) and we did not seek to identify the sources of heterogeneity of the results across countries. We acknowledge that the applied approach prevents us from understanding the potential mechanisms or differential susceptibility of the population, together with contextual differences across locations. Further studies are warranted to clarify this complex research question, including, for example, cause specific mortality and morbidity, and more complex two stage analyses. Finally, although the risk estimates were small they apply to the whole population, thus translating into substantial mortality impacts as shown in our estimates of excess mortality. By the same token, owing to the nature of the study design (time series analysis) the obtained excess mortality estimates refer to transient impact measures and not to the mortality burden or person years of life lost attributed to chronic exposure to ozone.^33^

### Conclusions

This large multi-country study provided evidence on the short term association between ozone and mortality. We also show that clean air policies with the enactment of air quality standards can constitute essential public health tools to minimise the health burden. In particular, our results suggest that ozone related health impacts can be largely preventable by attaining effective air quality standards in line with the WHO guideline. Moreover, interventions to further reduce ozone pollution would provide additional health benefits, even in regions that meet current regulatory standards and guidelines. These findings have important implications for the design of future public health actions; particularly, for example, in relation to the implementation of mitigation strategies to reduce the impacts of climate change.

**What is already known on this topic**

Studies on the short term association between ground level ozone and mortality have been mostly performed in a few locations, in limited geographical areas, and using various designs and modelling approaches
Although most of the studies found positive associations, results are heterogeneous, and a critical comparison across different countries and regions is made difficult by the limited statistical power and differences across studies
Estimates of the association are usually reported as relative risks, a summary measure that does not quantify the actual health impact and makes it difficult to evaluate comparative health benefits of different regulatory limits

**What this study adds**

This large multi-country study found increased mortality risks associated with exposure to ozone across locations and countries, with an average 0.18% per 10 µg/m^3^, reinforcing the evidence of a potential causal association
Risk estimates were translated in measures of excess mortality, and it was found that more than 6000 deaths each year, corresponding to 0.20% of the total mortality, would have been avoided in the 406 cities studied if countries had implemented stricter air quality standards compliant with the WHO guideline
Moreover, smaller but still substantial mortality impacts were found below WHO guideline, supporting the WHO initiative of encouraging countries to revisit current air quality guidelines and enforcing stronger emission restrictions to meet these recommendations

## Web extra

Extra material supplied by authors

**Supplementary information:** methods, e-tables 1-6, and e-figures 11-3

## Notes

Contributors: AG and HK are senior authors and contributed equally to this work. AG, YG, MH, and BA set up the collaborative network. AMV-C, AG, FS, and HK designed the study. AMV-C coordinated the work and took the lead in drafting the manuscript and interpreting the results. AG and FS developed the statistical methods. AMV-C conducted the statistical analysis. BA, AH, FS, AG, KK, ES, MS, AT, CI, VH, AS, JS, NS, RG, and EL provided substantial scientific input in interpreting the results and drafting the manuscript. CL, AM, YG, ST, EL, JK, AU, HO, EI, MP, VH, AS, KK, ES, MS, MS, MH, YH, CFSN, MH, JC, SS, JM, NS, RG, HK, AT, CI, BF, CA, MSR, MR, YLLG, BYC, AZ, JS, MB, and HK provided the data and contributed to the interpretation of the results and to the submitted version of the manuscript. AMV-C, AG, and HK are the guarantors. The corresponding author attests that all listed authors meet authorship criteria and that no others meeting the criteria have been omitted.

Ethical approval: Not required.

Data sharing: Data have been collected within the MCC (Multi-City Multi-Country) Collaborative Research Network (http://mccstudy.lshtm.ac.uk/) under a data sharing agreement and cannot be made publicly available. Researchers can refer to MCC participants listed as coauthors for information on accessing the data for each country. The R code for the analysis is available from the corresponding author.

The lead authors (AG and HK) affirm that the manuscript is an honest, accurate, and transparent account of the study being reported; that no important aspects of the study have been omitted; and that any discrepancies from the study as planned (and, if relevant, registered) have been explained.

Dissemination to participants and related patient and public communities: Dissemination of the findings will be carried out through press releases by the research institutions of the contributing authors.

Publisher’s note: Published maps are provided without any warranty of any kind, either express or implied. BMJ remains neutral with regard to jurisdictional claims in published maps.
