# Supplementary material for: Short term association between ozone and mortality: global two stage time series study in 406 locations in 20 countries
Source: BMJ. 2020 Feb 10;368:m108. doi: 10.1136/bmj.m108 (PMC7190035; doi:10.1136/bmj.m108)
Supplement: Supplementary file 1 — Supplementary information: methods, e-tables 1-6, and e-figures 11-3 [file vica050789.ww1.pdf]

# Online-only Supplements

**Table of contents**


- **eMethods 1.** Description of the data.
- **eMethods2.** Additional information on the statistical analysis.

- **eTable 1.** Complementary table with a description of other environmental data not included in Table 1.
- **eTable 2.** Description of the city-specific series.
- **eTable 3.** Overall and country-specific excess mortality fractions (%, 95% confidence interval) associated to ozone for the total (above 70 µg/m$^3$) and above specific thresholds consistent with current air quality standards (AQS).
- **eTable 4.** Overall and country-specific mortality fractions (and 95% confidence interval (CI)) associated to ozone between specific thresholds consistent with current AQS levels.
- **eTable 5.** Excess mortality associated to ozone for the total (above 70 µg/m$^3$) and above WHO guideline of 100 µg/m$^3$ in the 406 cities.
- **eTable 6.** Overall pooled estimates of ozone-mortality association (lag01) (95% confidence interval (CI)) expressed as relative risk (RR) per 10-µg/m$^3$ increase in ozone for each of the sensitivity analyses, except for the different approaches to control for temperature (reported in eFigure 3). Details on the analytical approach of each sub-analysis reported in eMethods 2.
-

- **eFigure 1.** Overall and country-specific short-term ozone-mortality association, expressed as relative risk (RR) per 10-ppb increase in ozone (maximum 8-hour average) (lag 01).
- **eFigure 2.** Results from the additional analyses. Comparison of the concentration-response shapes using linear and non-linear functions (left) and lag-response association up to 30 days (right). q-AIC linear: 10376950, Q-AIC non-linear: 10379020.
- **eFigure 3.** Overall and country-specific short-term ozone-mortality association, expressed as relative risk (RR) per 10-unit increase in ozone (DLM lag 01), using different control for temperature detailed in eMethods 2. (Black, main model (qAIC: 10376950); red, approach 1 (qAIC: 10377749); green, approach 2 (qAIC: 10380318); blue, approach 3 (qAIC: 10389132); pink, approach 4 (qAIC: 10387640)).

**eMethods 1 – Description of the MCC data**

Here we provide a detailed description of the mortality and environmental data used in the present study. As mentioned in the method section of the main manuscript, this data is included in a large dataset collected through the Multi-Country Multi-city (MCC) Collaborative Research Network (http://mccstudy.lshtm.ac.uk/). The dataset has been used in previous publications on temperature-related mortality and related topics (e.g. Gasparrini et al. 2015 Lancet) and a recent study on mortality associated to inhalable particles (Liu et al. 2019 NEJM). A detailed description of the air pollution data is provided in the latter. Below we describe for each country the specific data sources, the definition of the variables and the quality checks that were applied.

*Selection of the study locations*

We initially included 434 locations available in the MCC database at the time of the study, with available data on daily mortality, mean temperature and ozone (computed as 8-hour maximum). The selection was restricted to cities or metropolitan areas included in the MCC dataset (i.e., regions or provinces were excluded). We then selected 423 that provided data for more than 3 years between 1985 and 2015. Note that for most of the cities, air pollutants were available during specific time intervals which might not be homogeneous within country and not consistent with the mortality and temperature data. The main study period reported in Table 1 is defined as the time between the earliest year and latest year for which ozone data was available in each city within country and by air pollutant. Then, an exploratory analysis of the ozone data was performed as a quality check. From the 423 locations initially selected, we excluded 6 locations (1 in Italy and 5 in the US) with a percentage of missing values above 75% in the 30-days moving average of ozone. We then excluded 6 locations for which the temporal patterns in daily ozone did not follow the expected seasonal trends for this air pollutant, for instance showing abrupt steps, potentially due to changes in the monitors (e.g. relocation, change in the device) or other technical problems. Finally, 5 locations were excluded (1 in Mexico, 1 in South Africa, and 3 in the US) where mortality series presented more than 50% of missing counts. We specify below the locations that were excluded and the reason for exclusion in each country. For each sensitivity analysis using other pollutants, additional exclusions were performed depending on data availability of each air pollutant and weather variable.

*Characteristics of the air pollution data*

A unique daily series of each pollutant was derived from one or more monitoring stations of the national or regional network available in each city. When more than one monitor, an average daily measurement was derived. See information in each country on the number and characteristics of the monitor considered.

At the time of the data collection, countries provided air pollution levels in $\mu g/m^3$ for nitrogen dioxide and inhalable particles (PM10, PM2.5), and for ozone in $\mu g/m^3$, ppm or ppb. Prior to data analysis, ozone series were harmonized in terms of units of measure to $\mu g/m^3$. The conversion factors used were: 1 ppb = $10^{-3}$ ppm = 2 $\mu g/m^3$.

Refer to Table S1 for city-specific periods and descriptive summaries of the data.

*Description of the data in each country*

Australia (3 cities, 2000-2009)
Daily mortality, gathered from the Australian Bureau of Statistics, is represented by counts of deaths due to non-external causes (ICD-9: 0-799; ICD-10: A00-R99). Mean daily temperature (˚C) and relative humidity (%) were obtained from the Australian Bureau of Meteorology.

Hourly measurements of inhalable particulate matter with an aerodynamic diameter of 10 μm or less ($PM_{10}$), inhalable particulate matter with an aerodynamic diameter of 2.5 μm or less ($PM_{2.5}$) only between 2003 and 2009, nitrogen dioxide ($NO_2$), and ozone ($O_3$) were collected from urban monitoring stations run by local EPA. Daily $PM_{10}$, $PM_{2.5}$ and $NO_2$ measurements were computed as 24-hour average and daily maximum 8-hour average for $O_3$. In total, missing data amount for 0.0, 0.0, 1.4, 3.2, 8.3 and 4.0% of the mortality, mean temperature, relative humidity, $O_3$, $PM_{10,}$ $PM_{2.5}$ and $NO_2$ series, respectively.

Canada (26 metropolitan areas, 1986-2011)

Daily mortality, obtained from Statistics Canada through access to the Canadian Mortality Database, is represented by counts of deaths for all causes. Mean daily temperature (in ˚C), computed as the 24-hour average based on hourly measurements, and relative humidity (%) were obtained from Environment Canada. A single weather station was selected for each city using the airport monitoring station located closest to the CMA center. Hourly measures of $PM_{10}$ available in 16 cities between 2000-2011, $PM_{2.5}$ in 25 cities between 2006 and 2011, $NO_2$ in 25 cities in 2007-2011, and measures of $O_3$ were collected from monitors located in urban areas of the National Air Pollution Surveillance (NAPS) network of Environment Canada, a government institution that operates ground monitoring stations across Canada. Daily $PM_{10}$, $PM_{2.5}$ and $NO_2$ levels were computed as the 24-hour average and daily maximum 8-hour average for $O_3$ from hourly measurements in different stations, and then averaged across stations within the same CMA with no missing data, with an average of 4 stations per city. In total, missing data amount for 2.1, 1.0, 8.8, 4.1, 19.9, 7.1 and 7.2% of the mortality, mean temperature, relative humidity, $O_3$, $PM_{10}$, $PM_{2.5}$ and $NO_2$ series, respectively.

China (3 cities, 1996-2015 period)

Initially, we collected data from 4 Chinese cities from the Municipal Center for Disease Control. Daily mortality is represented by counts of deaths for non-external causes (ICD-9: 0-799; ICD-10: A00-R99). Each city had a different study period between 1996 and 2015, see in eTable2. Mean daily temperature (in ˚C), computed as the 24-hour average from hourly measurements, and relative humidity (%) were collected from the meteorological departments of each city. Measures of $PM_{10}$, $NO_2$ and ozone $O_3$ were collected from urban monitoring stations run by China National Environmental Monitoring Center. Daily $PM_{10}$ and $NO_2$ levels were computed as the 24-hour mean and daily maximum 8-hour average for $O_3$ using hourly measurements. The analysis was restricted to 3 cities with a minimum of three years of data. $PM_{10}$ and $NO_2$ measurements were available between 2005 and 2015. In total, missing data amount for 17.8, 17.8, 11.1, 21.9, 18.3 and 18.1% of the mortality, mean temperature, relative humidity, $O_3$, $PM_{10}$ and $NO_2$ series, respectively.

Czech Republic (1 city, 1994-2009)

Daily mortality is represented by counts of all-cause deaths obtained from the Czech Statistical Office and the Institute of Health Information and Statistics. Mean daily temperature (in ˚C) and relative humidity (%), computed as the average of observations in standard climatic terms (7:00, 14:00 and 21:00 local time) were collected by the Czech Hydrometeorological Institute. The average value was calculated according to formula (T07 + T14 + 2*T21)/4. Information about daily $PM_{10}$ and $NO_2$ levels computed as 24-hour average and maximum 8-hour average for $O_3$ were provided by the Czech Hydrometeorological Institute. The daily values were calculated from 4 stations (2 urban + 2 suburban). In total, missing data amount for 0.0, 0.0, 0.0, 0.1, 0.1 and 0.1% of the mortality, mean temperature, relative humidity, $O_3$, $PM_{10}$ and $NO_2$ series, respectively.

Estonia (4 cities, 2002-2015)

Daily mortality is represented by counts of deaths for non-external causes (ICD-9: 0-799; ICD-10: A00-R99) obtained from Estonian Causes of Death Registry. Mean daily temperature (in ˚C) and relative humidity (%) were computed as the 24-h average of hourly measurements collected from Estonian Environment Agency. A single weather station located nearby the urban area was selected for each city. Hourly measurements $PM_{10}$, $PM_{2.5}$, $NO_2$ and $O_3$ were collected from urban background stations run by the Estonian Environmental Research Centre. Daily $PM_{10}$, $PM_{2.5}$ and $NO_2$ levels were computed as 24-hour average and daily maximum 8-hour average for $O_3$; for each pollutant, city average among monitoring stations was calculated. $PM_{10}$ and $NO_2$ measurements were available between 2009 and 2015 and $PM_{2.5}$ between 2010 and 2015. In total, missing data amount for 0.0, 0.0, 0.0, 5.1, 6.2, 7.7 and 7.8% of the mortality, mean temperature, relative humidity, $O_3$, $PM_{10,}$ $PM_{2.5}$ and $NO_2$ series, respectively.

France (18 cities, 2000-2010)

Daily mortality, obtained from French National Institute of Health and Medical Research (CepiDC), is represented by counts of deaths for all causes. Mean daily temperature (in ˚C), computed as the mean of the minimal and maximal temperature, and relative humidity (%) was obtained from Meteo France. Hourly measurements of $PM_{10}$ and $O_3$ were collected through the French local air quality monitoring network (Associations Agréées de Surveillance de la Qualité de l'Air AASQA). For $PM_{10}$, we used only urban stations, and for $O_3$, urban and peri-urban stations. Daily $PM_{10}$ levels were computed as 24h average and daily maximum 8-hour average for $O_{3.}$ Measurements were obtained from multiple stations (with different numbers for each city). $PM_{10}$ measurements were available between 2001 and 2010. In total, missing data amount for 0.0, 0.1, 0.1, 0.1 and 1.8% of the mortality, mean temperature, relative humidity, $O_3$ and $PM_{10}$ series, respectively.

Germany (12 cities, 1993-2015)

Daily mortality, obtained from the Research Data Centres of the Federation and the Federal States of Germany (Forschungsdatenzentrum der Statistischen Ämter des Bundes und der Länder), is represented by counts of deaths for all causes. Mean daily temperature (in ˚C), computed as the 24-h average based on hourly measurements, was obtained from the Climate Data Centre of the German National Meteorological Service (Deutscher Wetterdienst). Where several weather stations existed within the city boundaries, stations closest to the city centre were chosen, provided that measurements were available for the whole study period. Hourly measurements of $PM_{10}$, $PM_{2.5,}$ $NO_2$ and $O_3$ were collected through the German Environment Agency (Umweltbundesamt) from urban background stations. Daily $PM_{10}$, $PM_{2.5}$ and $NO_2$ levels were computed as 24-hour average and daily maximum 8-hour average for $O_3$. Measurements were obtained from multiple stations (with different numbers for each city). $PM_{10}$ measurements were available between 2003 and 2015, $PM_{2.5}$ between 2010 and 2015 only in 11 cities and $NO_2$ between 1994 and 2015. In total, missing data amount for 0.0, 0.0, 5.1, 4.3, 5.8 and 5.3% of the mortality, mean temperature, $O_3$, $PM_{10}$, $PM_{2.5}$ and $NO_2$ series, respectively.

Greece (1 city, 2001-2010)

Daily mortality is represented by counts of deaths for all causes (ICD-9: 0-799; ICD-10: A00-R99) collected by Hellenic Statistical Authority. Mean daily temperature (in ˚C) and relative humidity (%) were computed as the 24-h average based on hourly measurements collected from the National observatory of Athens (http://www.noa.gr/) from site "Thisio" located in the city of Athens. Hourly measurements $PM_{10}$, $PM_{2.5}$, $NO_2$ and ozone ($O_3$) were obtained from the Ministry of Environment and Energy fixed site monitoring network. Urban or suburban fixed monitoring background or traffic sites with at least 75% complete information per year of the study period were selected Daily $PM_{10}$, $PM_{2.5}$ and $NO_2$ levels were computed as 24-hour

average and daily maximum 8-hour average for $O_3$. $PM_{2.5}$ was only available between 2007 and 2010. In total, missing data amount for 0.0, 6.0, 5.9, 0.0, 1.5, 1.8, and 0.0% of the mortality, mean temperature, relative humidity, $O_3$, $PM_{10}$, $PM_{2.5}$ and $NO_2$ series, respectively.

Italy (9 cities, 2006-2015)

Data on 14 Italian cities were initially included, but only 9 cities were selected according to the selection criteria. Daily mortality, obtained from local mortality registries and from the rapid mortality surveillance system, is represented by counts of deaths for all causes (ICD-9: 0-799; ICD-10: A00-R99). Mean daily temperature (in ˚C) was computed as the 24-h average based on 6-h measurements obtained from the Meteorological Service of the Italian Air Force. A single weather station was selected for each city, using the airport monitoring station located closest to the city center. Hourly measurements of $PM_{10}$ and $O_3$ were obtained from the same period. Daily $PM_{10}$ levels were computed as 24h average and daily maximum 8-hour average for $O_3$. In total, missing data amount for 0.0, 0.5, 1.7 and 1.7% of the mortality, mean temperature, $O_3$ and $PM_{10}$ series, respectively.

Japan (45 cities, 2011-2015)

Data was initially collected from each capital city of the 47 prefectures of Japan, but 2 locations with a short study period (less than 3 years) were excluded. Daily mortality, obtained from computerized death certificate data from the Ministry of Health, Labour and Welfare, Japan, is represented by counts of deaths for all causes. Mean daily temperature (in ˚C) and relative humidity (in %), computed as the 24-hour average based on hourly measurements, were obtained from the Japan Meteorological Agency. A single weather station located within the urban area of the capital city was selected for each prefecture. Hourly measurements of $PM_{10}$, $PM_{2.5}$, $NO_2$ and $O_3$ were collected from the urban monitors within the capital cities maintained by the Ministry of the Environment of Japan. Daily $PM_{10}$, $PM_{2.5}$ and $NO_2$ levels were computed as 24-hour average and daily maximum 8-hour average for $O_3$. $PM_{10}$ measurements were available between 2013 and 2015, and $PM_{2.5}$ between 2014 and 2015. In total, missing data amount for 0.0, 0.1, 0.1, 0.9, 3.1, 6.4, and 0.8% of the mortality, mean temperature, relative humidity, $O_3$, $PM_{10}$, and $NO_2$ series, respectively.

Mexico (7 cities, 2000-2012)

Daily mortality, obtained from National Institute of Statistics, Geography and Informatics, is represented by counts of deaths for all causes. Mean daily temperature (in ˚C) and relative humidity (%) were computed as the 24-hour average based on hourly measurements collected through the Servicio Meteorológico Nacional (SMN) and the Instituto Nacional de Ecología y Cambio Climático (INECC). Hourly measurements of $PM_{10}$, $PM_{2.5}$ and $O_3$ were obtained from urban monitors of the local monitoring network. Daily $PM_{10}$ and $PM_{2.5}$ levels were computed as the 24-hour mean and daily maximum 8-hour average for $O_3$ from hourly measurements. Data from 9 cities were initially included, but 2 locations were eventually excluded due to poor quality of $O_3$ data. $PM_{10}$ measurements were available between 2010 and 2012, and $PM_{2.5}$ between 2011 and 2012 only in 5 cities. In total, missing data amount for 0.0, 17.3, 7.0 and 5.5% of the mortality, mean temperature, $O_3$ and $PM_{10}$ series, respectively.

Portugal (2 cities, 1997-2012)

Daily mortality, obtained from Statistics Portugal, is represented by counts of deaths for non-external causes only (ICD-9: 0-799; ICD-10: A00-R99). Mean daily temperature (in ˚C) was computed as the 24-hour average based on hourly measurements collected from the National Oceanic and Atmospheric Administration (NOAA). Hourly measurements of $PM_{10}$, $PM_{2.5}$, $NO_2$ and $O_3$ were gathered from the “online database of air quality” through Portuguese Environment Agency from urban monitors. Daily $PM_{10}$, $PM_{2.5}$ and $NO_2$ levels were computed

as the 24-hour mean and daily maximum 8-hour average for $O_3$ from hourly measurements. $PM_{10}$ and $NO_2$ measurements were available between 1999 and 2012, and $PM_{2.5}$ between 2004 and 2012 only in 1 city. In total, missing data amount for 0.0, 0.1, 5.0, 4.8, 8.2 and 0.1% of the mortality, mean temperature, $O_3$, $PM_{10}$, $PM_{2.5}$ and $NO_2$ series, respectively.

South Korea (7 cities, 1999-2015)

Daily mortality was obtained from the Korea National Statistics Office, and is represented by counts of deaths for all causes. Mean daily temperature (in ˚C) and relative humidity (%) were computed as the 24-hour average based on hourly measurements. Measures of $PM_{10}$, $NO_2$ and $O_3$ were available in the period 1999-2015 from monitors of the National Institute of Environmental Research. Daily $PM_{10}$ and $NO_2$ levels were computed as the 24-hour mean and daily maximum 8-hour average for $O_3$ from hourly measurements. In total, missing data amount for 0.9, 0.9, 0.9, 0.9, 1.4 and 0.9% of the mortality, mean temperature, relative humidity, $O_3$, $PM_{10}$ and $NO_2$ series, respectively.

South Africa (5 cities, 2004-2013)
Daily mortality, provided by Statistics South Africa, is represented by counts of all recorded deaths from all causes at the level of the district municipality. Mean daily temperature (in ˚C) was computed as the average between daily minimum and maximum collected from the Agricultural Research Council of South Africa and the National Oceanic and Atmospheric Administration (NOAA). Hourly measurements of $PM_{10}$ and $O_3$ were collected at sites managed by the Department of Environmental Affairs (DEA) run by South African Weather Service (SAWS), the eThekwini Municipality, The City of Johannesburg, Sasol, and ESKOM. The eThekwini sites are within the coastal city of Durban on the Indian Ocean. The other sites are in the urban and industrial highveld region of South Africa. The monitoring sites in the industrial highveld include a range of site characteristics, including regional background, traffic, urban, power plant plume, industrial, residential, and low-income residential sites. Daily $PM_{10}$ levels were computed as the 24-hour mean, and daily maximum 8-hour running average for $O_3$ from the respective provided hourly measurements. The average 24-hour mean or daily maximum 8-hour running average values per district municipality (DM) were then calculated from all sites within each DM. All air quality data, except for the ESKOM run stations, were accessed through SAAQIS (http://www.saaqis.org.za/), which is run and hosted by SAWS. Data from 6 cities in South Africa were initially included, but 1 location was eventually excluded due to the poor quality of the ozone data. In total, 7.3%, 14.4% and 10.1% of the mortality, mean temperature, $O_3$ and $PM_{10}$ series was missing, respectively. We thank the Statistics South Africa for providing the mortality data, the Agricultural Research Council for providing the meteorological data, and DEA, SAAQIS, SAWS as well as all the data providers (ESKOM, Sasol, City of Johannesburg, and the eThekwini Municipality) for providing the air pollution data. Statistics South Africa had no had no role in the study design, data analysis or interpretation.

Spain (48 cities, 2004-2014)

Data was collected from the 48 capital cities of each province in Spain. Daily mortality, obtained from Spain National Institute of Statistics, is represented by counts of deaths for non-external causes (ICD-9: 0-799; ICD-10: A00-R99). Mean daily temperature (in ˚C), computed as the 24-hour average based on hourly measurements, and was obtained from weather stations of the Spain National Meteorology Agency. A single weather station, located within the urban area or at the near airport, was selected for each city. Hourly measurements of $PM_{10}$, $PM_{2.5}$, $NO_2$ and $O_3$ were collected from the free national repository (Magrama) from urban and suburban monitors. Daily $PM_{10}$, $PM_{2.5}$ and $NO_2$ levels were computed as 24-hour average (only for 43, 20 and 45 cities, respectively) and daily maximum 8-hour average for

$O_3$. $PM_{10}$ and $NO_2$ were collected between 2010 and 2014, and $PM_{2.5}$ between 2013 and 2014. In total, missing data amount for 0.0, 0.5, 5.9, 9.7, 11.8 and 6.7% of the mortality, mean temperature, $O_3$, $PM_{10}$ , $PM_{2.5}$ and $NO_2$ series, respectively.

Sweden (1 city, 1990-2010)

Daily mortality, obtained from the Swedish Cause of Death Register at the Swedish National Board of Health and Welfare, is represented by counts of deaths for all causes. Mean daily temperature (in ˚C) and relative humidity (%), computed as the 24-hour average based on hourly measurements, were obtained from the Environment and Health Administration. A single weather station, located at Torkel Knutssongatan in Central Stockholm, was selected. Hourly measurements of $PM_{10}$, $PM_{2.5}$, $NO_2$ and $O_3$ were collected from the main urban background monitor run by the local monitoring network. Daily $PM_{10}$, $PM_{2.5}$ and $NO_2$ levels were computed as the 24-hour average and for $O_3$ as 8-hour maximum. $PM_{10}$ and $PM_{2.5}$ were collected between 1993 and 2014, and 2001 and 2010, respectively. In total, missing data amount for 0.0, 0.2, 0.3, 4.2, 9.9, 9.3 and 0.1% of the mortality, mean temperature, $O_3$, $PM_{10}$ and $NO_2$ series, respectively.

Switzerland (8 cities, 1995-2013)

Data on the 8 cities in Switzerland was collected. We extended the catching area of Lugano to the small municipalities around the main city with similar altitude. Daily mortality, provided by the Federal Office of Statistics (Switzerland), is represented by counts of non-external deaths other than accidents (ICD-10codes A00-R99, V01-V99, W00-X59). Mean daily temperature (in ˚C) and relative humidity (%), computed as the 24-hour average based on hourly measurements, were obtained from the IDAWEB database (a service provided by MeteoSwiss, the Swiss Federal Office of Meteorology and Climatology). A single weather station located within or near the urban area was selected for each city. Hourly measurements of $PM_{10}$, $PM_{2.5}$, $NO_2$ and $O_3$ were provided by the Immissionsdatenbank Luft (IDB, Federal Office of the Environment, Bern, Switzerland). Daily $PM_{10}$, $PM_{2.5}$ and $NO_2$ levels were computed as the 24-hour average from urban monitoring stations, and as 8h-maximum for $O_3$ from urban and sub-urban monitoring stations. $PM_{10}$ and $PM_{2.5}$ were collected between 2003 and 2013. In total, missing data amount for 0.0, 0.0, 0.0, 4.8, 1.8, 6.9 and 0.5% of the mortality, mean temperature, relative humidity, $O_3$, $PM_{10,}$ $PM_{2.5}$ and $NO_2$ series, respectively.

Taiwan (3 cities, 2008-2014)

Daily mortality, obtained from the Department of Health in Taiwan, is represented by counts of deaths for all causes. Mean daily temperature (in ˚C) and relative humidity (%) were computed as the 24-hour average based on hourly measurements. Hourly measurements of $PM_{10}$, $PM_{2.5}$, $NO_2$ and $O_3$ were obtained from urban monitors of the local monitoring network. Daily $PM_{10}$, $PM_{2.5}$ and $NO_2$ levels were computed as the 24-hour average and for $O_3$ as 8-hour maximum. Data were pooled from 1 meteorological station and 11 air quality monitoring stations in Kaohsiung, 2 meteorological station and 5 air quality monitoring stations in Taichung, and 3 meteorological station and 15 air quality monitoring stations in Taipei. In total, missing data amount for 0.0, 0.0, 0.0, 0.0, 0.0, 0.1 and 0.0% of the mortality, mean temperature, relative humidity, $O_3$ $PM_{10}$, $PM_{2.5}$ and $NO_2$ series, respectively.

United Kingdom (15 conurbations, 1993-2006)

Daily mortality, gathered from Office for National Statistics, is represented by counts of deaths for all causes. Mean daily temperature (in ˚C) and relative humidity (in %) were obtained from the Meteorological Department obtained from British Atmospheric Data Centre. Series for each city daily mean temperatures were similarly constructed from all meteorological stations providing data for at least 75% of days. All data on air pollution levels were downloaded from

the UK Air Quality Archive, which reports results from the network of monitoring stations operated by the UK government. Hourly measurements of $PM_{10}$ and $O_3$ were available in the same period from urban and sub-urban monitoring stations. Daily $PM_{10}$ levels were computed as the 24-hour average from urban monitoring stations, and as 8hour-maximum for $O_3$. In total, missing data amount for 0.0, 0.0, 13.6, 7.3 and 7.9% of the mortality, mean temperature, relative humidity, $O_3$ and $PM_{10}$ series, respectively.

United States (188 cities, 1985-2006)

Daily mortality is represented by counts of deaths for all causes. Mean daily temperature (in ˚C) and relative humidity (%), computed as the 24-hour average based on hourly measurements, were obtained from the National Climatic Data Center (NCDC) of the National Oceanic and Atmospheric Administration (NOAA). Hourly measurements of $PM_{10}$, $PM_{2.5}$, $NO_2$ and $O_3$ were gathered from the U.S. Environmental Protection Agency (EPA) Air Quality System (AQS), from urban and sub-urban monitoring stations. Daily $PM_{10}$, $PM_{2.5}$ and $NO_2$ levels were computed as the 24-hour average from urban monitoring stations (only for 176 and 137 cities), and as 8-hour-maximum for $O_3$ from monitors located in the county or set of contiguous counties in which the city is located. 91 out the 182 US cities registered daily $O_3$ levels only during the summer period. Data from 205 cities were initially collected, but 8 locations with a short study period (less than 3 years) were excluded, along with other 9 locations with a poor quality of $O_3$ data. $PM_{10}$ and $PM_{2.5}$ were collected between 2005 and 2006, and 2004 and 2006, respectively. In total, missing data amount for 0.8, 0.8, 5.2, 23.4, 64.7, 41.2 and 14.5% of the mortality, mean temperature, relative humidity, $O_3$, $PM_{10}$, $PM_{2.5}$ and $NO_2$ series, respectively.

## eMethods 2. Additional information on the statistical analysis

Control for temperature in the main model

We control for temperature with distributed lag non-linear model (DLNM) of mean daily temperature. According to a previous study of MCC collaborative network (Gasparrini et al. Lancet 2015), the non-linear and delayed temperature-mortality association was modelled through a quadratic b-spline with internal knots in the $10^{th}$, $75^{th}$, $90^{th}$ percentiles in the exposure-response dimension, and natural spline with 3 equally-spaced internal knots in the log scale of the lag dimension, up to 21 days of lag.

Description of the additional analysis

Here we provide a detailed description of specific features of the additional analyses.

As described in the main manuscript, we explored potential non-linearity and delayed associations between ozone and mortality. The modelling choices selected (specifications of modelling function of ozone) for each sub-analysis were chosen based on q-AIC among different combinations of smoothing functions and placement and number of knots for the exposure-response and lag dimension, respectively.

- Modelling choices for the non-linear concentration-response function: natural cubic spline and quadratic b-spline with 1 or 2 internal knots placed in 50 and 60 $\mu g/m^3$.
- Modelling choices for the lag-association function: natural cubic spline with 1, 2 or 3 internal knots placed equally-spaced in the natural or log scale of the lag dimension up to 30 days.

City-specific non-linear exposure-response associations and lag-response associations obtained in these two additional analyses were pooled following the same method described in the main manuscript for the final model. More details on this methodology can be found in Gasparrini et al. 2013 BMC Med Res Methodol.

Description of the sensitivity analyses

*Different control for time trends*: natural spline with 4 and 10 degrees of freedom per year.

*Control for* $PM_{10}$, $PM_{2.5}$ *and* $NO_2$: air pollutants were included one by one in the main model as linear unconstrained distributed-lag linear models (DLMs) for the same and previous day of the exposure (lags 0 and 1).

*Control for relative humidity*: we controlled for relative humidity in the model as a linear term of lag0.

*Modelling approaches used to control for temperature*:

- Main model: quadratic b-spline with internal knots in the $10^{th}$, $75^{th}$, $90^{th}$ percentiles in the exposure-response dimension, and natural spline with 3 equally-spaced internal knots in the log scale of the lag dimension, up to 21 days of lag.
- Approach 1: quadratic b-spline with internal knots in the $10^{th}$, $75^{th}$, $90^{th}$ percentiles in the exposure-response dimension, and natural spline with 3 equally-spaced internal knots in the log scale of the lag dimension, up to 13 days of lag.
- Approach 2: quadratic b-spline with 3 internal knots equally-spaced quantiles in the exposure-response dimension, and natural spline with 1 internal knot equally-spaced in the log scale of the lag dimension, up to 13 days of lag.
- Approach 3: natural cubic spline with 3 degrees of freedom of the moving average lag013.
- Approach 4: natural cubic spline with 3 degrees of freedom of the moving average lag03.

*References*

Gasparrini A, Guo Y, Hashizume M, Lavigne E, Zanobetti A, Schwartz J, Tobias A, Tong S, Rocklöv J, Forsberg B, Leone M, De Sario M, Bell ML, Guo YL, Wu CF, Kan H, Yi SM, de Sousa Zanotti Stagliorio Coelho M, Saldiva PH, Honda Y, Kim H, Armstrong B. Mortality risk attributable to high and low ambient temperature: a multicountry observational study. Lancet. 2015 Jul 25;386(9991):369-75. doi: 10.1016/S0140-6736(14)62114-0.

Gasparrini A, Armstrong B. Reducing and meta-analysing estimates from distributed lag non-linear models. BMC Med Res Methodol. 2013 Jan 9;13:1. doi: 10.1186/1471-2288-13-1.

Liu C, Chen R, Sera F, Vicedo-Cabrera AM, Guo Y, Tong S, Coelho MSZS, Saldiva PHN, Lavigne E, Matus P, Valdes Ortega N, Osorio Garcia S, Pascal M, Stafoggia M, Scortichini M, Hashizume M, Honda Y, Hurtado-Díaz M, Cruz J, Nunes B, Teixeira JP, Kim H, Tobias A, Íñiguez C, Forsberg B, Åström C, Ragettli MS, Guo YL, Chen BY, Bell ML, Wright CY, Scovronick N, Garland RM, Milojevic A, Kyselý J, Urban A, Orru H, Indermitte E, Jaakkola JJK, Ryti NRI, Katsouyanni K, Analitis A, Zanobetti A, Schwartz J, Chen J, Wu T, Cohen A, Gasparrini A, Kan H. Ambient Particulate Air Pollution and Daily Mortality in 652 Cities. N Engl J Med. 2019.

**eTable 1.** Complementary table with a description of other environmental data not included in Table 1.

| | $PM_{10}$ (Median [IQR]) | $PM_{2.5}$ (Median [IQR]) | $NO_2$ (Median [IQR]) | Relative humidity (Median [IQR]) |
|---|---|---|---|---|
| *Australia* | 18.2 [14.5; 23.2] | 6.0 [4.3; 8.4] | 21.4 [14.1; 27.9] | 70.1 [62.6; 77.1] |
| *Canada* | 12.0 [7.6; 20.3] | 7.3 [4.7; 11.5] | 13.9 [9.5; 19.4] | 73.7 [64.7; 82.3] |
| *China* | 66.7 [46.5; 97.2] | -- | 37.6 [28.0; 50.5] | 76.7 [69.1; 83.6] |
| *Czech Republic* | 29.4 [19.9; 44.7] | -- | 30.8 [24.2; 38.7] | 78.0 [68.0; 86.0] |
| *Estonia* | 14.4 [9.3; 21.8] | 7.4 [4.3; 11.8] | 11.4 [7.6; 16.7] | 84.2 [74.8; 91.2] |
| *France* | 18.7 [13.9; 25.1] | -- | -- | 76.2 [67.5; 84.1] |
| *Germany* | 20.1 [14.3; 28.7] | 12.1 [8.1; 18.3] | 29.6 [21.8; 38.4] | -- |
| *Greece* | 39.5 [29.5; 53.1] | -- | 50.2 [39.6; 61.6] | 66.0 [54.0; 75.4] |
| *Italy* | 28.2 [21.0; 40.6] | -- | -- | -- |
| *Japan* | 16.8 [11.7; 23.9] | 12.6 [8.4; 18.3] | 17.5 [12.7; 24.4] | 69.6 [60.6; 77.7] |
| *Mexico* | 53.4 [38.5; 72.7] | 25.6 [19.0; 33.1] | -- | 60.8 [50.0; 71.1] |
| *Portugal* | 27.2 [18.5; 40.8] | 11.0 [7.1; 18.0] | 26.9 [17.2; 39.0] | -- |
| *South Africa* | 48.1 [31.4; 73.1] | 27.0 [18.2; 39.6] | -- | -- |
| *South Korea* | 46.0 [33.3; 63.0] | -- | 44.5 [33.6; 58.5] | 65.0 [52.3; 75.6] |
| *Spain* | 25.2 [19.2; 32.7] | 11.5 [8.1; 16.2] | 26.4 [20.2; 34.0] | -- |
| *Sweden* | 12.5 [9.3; 17.9] | 6.6 [4.7; 9.5] | 26.8 [20.0; 34.8] | 79.6 [68.4; 87.6] |
| *Switzerland* | 21.0 [13.9; 31.1] | 16.0 [10.4; 24.6] | 32.0 [23.8; 41.8] | 75.5 [66.4; 83.3] |
| *Taiwan* | 54.6 [37.5; 74.1] | 32.0 [20.5; 43.8] | 35.9 [27.6; 45.6] | 75.3 [70.5; 79.8] |
| *UK* | 23.3 [18.0; 31.5] | -- | -- | 74.2 [64.2; 83.3] |
| *USA* | 25.9 [17.9; 36.7] | 11.6 [7.6; 17.2] | 31.7 [23.0; 42.4] | 66.5 [56.5; 76.6] |

$PM_{10}$: inhalable particulate matter with an aerodynamic diameter of 10 µm or less, µg/m$^3$. $PM_{2.5}$: inhalable particulate matter with an aerodynamic diameter of 2.5 µm or less, µg/m$^3$. $NO_2$: nitrogen dioxide, µg/m$^3$. IQR: interquartile range. Relative humidity, in %. Detailed description of the data provided in eMethods 1. City-specific descriptive summaries reported in eTable 2.

**eTable 2.** Description of city-specific series.

| City | Country | Period | N deaths | Ozone (μg/m$^3$) Median [IQR] | $PM_{10}$ (μg/m$^3$) Median [IQR] | $PM_{2.5}$ (μg/m$^3$) Median [IQR] | $NO_2$ (μg/m$^3$) Median [IQR] | Mean temperature (˚C) Median [IQR] | Relative humidity (%) Median [IQR] |
|---|---|---|---|---|---|---|---|---|---|
| Brisbane | Australia | 2000-2009 | 89299 | 35.1 [29.0; 41.6] | 17.2 [14.6; 20.5] | 5.6 [4.3; 7.3] | 12.9 [9.5; 17.9] | 20.8 [17.1; 23.5] | 69.5 [63.8; 75.2] |
| Melbourne | Australia | 2000-2009 | 196355 | 31.2 [23.8; 38.9] | 17.5 [13.6; 23.3] | 6.3 [4.6; 8.9] | 24.6 [16.4; 30.8] | 15.5 [12.3; 19.1] | 69.7 [62.0; 77.3] |
| Sydney | Australia | 2000-2009 | 227873 | 27.4 [19.6; 35.3] | 19.8 [15.2; 25.8] | 6.1 [4.1; 9.0] | 26.7 [16.4; 34.9] | 18.8 [15.1; 21.9] | 71.1 [62.0; 78.7] |
| Abbotsford | Canada | 1986-2011 | 27098 | 66.6 [49.0; 80.4] | 11.6 [8.3; 16.5] | 5.1 [3.4; 7.2] | 12.3 [9.1; 15.6] | 10.5 [6.2; 15.7] | 76.2 [68.4; 84.3] |
| Calgary | Canada | 1986-2011 | 132883 | 76.4 [62.7; 92.6] | 20.6 [13.8; 29.7] | 8.4 [5.4; 12.3] | 21.7 [16.2; 28.7] | 5.6 [-1.8; 12.9] | 62.0 [50.8; 73.5] |
| Edmonton | Canada | 1986-2011 | 154899 | 72.5 [54.9; 92.1] | 17.6 [11.4; 26.9] | 7.5 [4.8; 12.2] | 19.1 [13.4; 26.8] | 5.4 [-3.5; 13.9] | 69.9 [61.7; 78.1] |
| Halifax | Canada | 1986-2011 | 71264 | 60.8 [49.0; 74.5] | NA | 7.1 [4.8; 9.9] | 14.7 [10.8; 19.3] | 7.1 [-0.4; 14.9] | 79.7 [69.5; 88.9] |
| Hamilton | Canada | 1986-2011 | 113876 | 72.5 [54.9; 98.0] | 27.2 [18.5; 38.7] | 9.0 [5.6; 14.3] | 17.6 [12.7; 23.0] | 8.3 [0; 17.2] | 73.2 [64.4; 81.7] |
| Kingston | Canada | 1988-2011 | 36438 | 70.6 [54.9; 92.1] | NA | 6.8 [4.3; 11.1] | 4.3 [3.1; 6.1] | 8.1 [-0.2; 17] | 70.6 [61.4; 79.2] |
| Kitchener-Waterloo | Canada | 1986-2011 | 71387 | 72.5 [54.9; 96.0] | NA | 7.5 [4.4; 12.9] | 12.0 [7.5; 18.8] | 7.4 [-1; 16.2] | 72.2 [63.3; 80.2] |
| London Ontario | Canada | 1986-2011 | 96799 | 68.6 [51.0; 96.0] | 15.8 [11.2; 23.4] | 8.4 [5.2; 13.2] | 15.4 [9.7; 21.7] | 8.7 [0; 17.3] | 75.8 [68.3; 82.9] |
| Montreal | Canada | 1986-2009 | 255272 | 78.4 [58.8; 98.0] | 20.5 [15.3; 28.2] | 8.3 [5.3; 13.6] | 17.5 [12.8; 23.0] | 7.7 [-2.1; 17.1] | 70.2 [61.4; 78.6] |
| Niagara | Canada | 1988-1996 | 31770 | 74.5 [54.9; 105.8] | NA | NA | NA | 8.9 [1.1; 17.9] | 78.3 [70.8; 86.3] |
| Oakville | Canada | 1986-2011 | 58991 | 72.5 [54.9; 96.0] | NA | 7.0 [4.3; 11.4] | 14.6 [9.8; 20.0] | 8.7 [0.9; 17.5] | 69.5 [61.4; 78.5] |
| Oshawa | Canada | 1986-2011 | 72386 | 70.6 [54.9; 88.2] | NA | 7.2 [4.3; 12.2] | 15.3 [8.8; 23.1] | 7.9 [0; 16.6] | NA |
| Ottawa | Canada | 1986-2011 | 136955 | 66.6 [51.0; 84.3] | NA | 6.0 [3.5; 10.2] | 16.0 [8.9; 23.3] | 7.6 [-2.5; 17.1] | 71.0 [61.5; 80.6] |
| Regina | Canada | 1986-2011 | 49530 | 52.9 [39.2; 68.6] | 19.4 [11.9; 29.4] | 6.7 [4.4; 9.6] | 12.5 [9.0; 17.4] | 4.7 [-6.4; 14.5] | 72.3 [61.4; 82.4] |
| Sarnia | Canada | 1986-2011 | 28656 | 76.4 [60.8; 100.0] | 16.8 [10.5; 24.6] | 11.5 [7.5; 17.7] | 13.3 [8.7; 19.6] | 9.1 [0.7; 17.8] | 77.2 [69.4; 84.3] |
| Sudbury | Canada | 1986-2011 | 40907 | 70.6 [58.8; 88.2] | NA | 4.0 [2.4; 6.8] | 7.9 [5.0; 11.7] | 5.1 [-5; 15.1] | 73.0 [62.3; 83.1] |
| Saint John NB | Canada | 1986-2011 | 44302 | 72.5 [58.8; 86.2] | 12.5 [8.3; 18.8] | 5.7 [3.5; 9.0] | 6.4 [3.7; 10.4] | 6.2 [-1.7; 13.7] | 76.5 [66.3; 86.1] |
| St John's NFL | Canada | 1989-2011 | 48656 | 62.7 [51.0; 72.5] | NA | 4.4 [2.9; 6.4] | 7.5 [4.4; 12.1] | 5.1 [-1.1; 12.1] | 83.5 [75.0; 90.8] |
| Sault Ste Marie | Canada | 1986-2011 | 28838 | 68.6 [56.8; 84.3] | 13.8 [9.2; 22.1] | 5.3 [2.9; 9.2] | 8.3 [4.3; 13.8] | 5.7 [-3; 14.7] | 78.5 [71.0; 85.2] |
| Saskatoon | Canada | 1986-2011 | 56891 | 56.8 [43.1; 72.5] | NA | 5.2 [3.4; 7.8] | 11.5 [8.0; 16.0] | 4.4 [-7; 14.1] | 73.0 [62.5; 82.5] |

| City | Country | Period | N deaths | Ozone (μg/m³) Median [IQR] | $PM_{10}$ (μg/m³) Median [IQR] | $PM_{2.5}$ (μg/m³) Median [IQR] | $NO_2$ (μg/m³) Median [IQR] | Mean temperature (˚C) Median [IQR] | Relative humidity (%) Median [IQR] |
|---|---|---|---|---|---|---|---|---|---|
| Thunder Bay | Canada | 1986-2011 | 35663 | 64.7 [52.9; 78.4] | 11.7 [7.8; 14.1] | 4.9 [3.0; 7.8] | 10.0 [6.3; 14.9] | 4.2 [-5.7; 13.4] | 74.4 [64.8; 82.6] |
| Toronto | Canada | 1986-2011 | 673074 | 84.3 [66.6; 109.8] | 18.2 [13.6; 25.0] | 8.1 [5.1; 13.0] | 21.5 [16.2; 27.1] | 8.8 [0.5; 17.7] | 71.8 [63.7; 79.5] |
| Victoria | Canada | 1986-2011 | 84747 | 62.7 [49.0; 78.4] | 12.1 [9.2; 16.1] | 5.7 [3.9; 8.4] | 9.7 [6.3; 14.1] | 9.9 [6.3; 14.5] | 78.2 [70.0; 86.0] |
| Vancouver | Canada | 1986-2011 | 329577 | 70.6 [60.8; 84.3] | 11.6 [8.6; 15.5] | 5.5 [3.8; 7.7] | 16.9 [13.7; 20.6] | 10.2 [6.2; 15.4] | 79.3 [72.3; 86.5] |
| Windsor | Canada | 1986-2011 | 65259 | 70.6 [47.0; 101.9] | 21.0 [15.3; 29.7] | 9.5 [5.8; 14.5] | 20.4 [14.6; 27.1] | 10.4 [1.6; 19.3] | 70.6 [62.5; 79.2] |
| Winnipeg | Canada | 1986-2011 | 168512 | 62.7 [51.0; 80.4] | 12.0 [7.6; 20.3] | 6.4 [4.3; 9.2] | 11.8 [7.9; 17.1] | 4.8 [-7.4; 15.5] | 73.1 [64.2; 82.2] |
| Hong Kong | China | 1996-2002 | 215242 | 31.4 [19.0; 50.6] | 45.4 [31.7; 66.5] | NA | 14.7 [9.6; 22.2] | 24.7 [19.8; 27.8] | 79.0 [74.0; 84.0] |
| Shanghai | China | 2001-2015 | 515429 | 68.2 [42.8; 100.2] | 75.0 [51.8; 114.1] | NA | 53.0 [39.3; 71.2] | 18.3 [10; 24.4] | 73.0 [64.4; 80.8] |
| Suzhou | China | 2005-2008 | 49984 | 48.3 [21.6; 81.7] | 79.6 [56.0; 111.0] | NA | 45.0 [35.0; 58.0] | 18.3 [9.2; 24.9] | 78.0 [69.0; 86.0] |
| Prague | Czech Republic | 1994-2009 | 214062 | 69.3 [47.4; 95.0] | 29.4 [19.9; 44.7] | NA | 30.8 [24.2; 38.7] | 9.2 [2.5; 15.3] | 78.0 [68.0; 86.0] |
| Kohtla-Jarve | Estonia | 2002-2015 | 12199 | 55.0 [43.7; 67.4] | 12.4 [8.1; 19.2] | 5.0 [2.6; 8.3] | 5.1 [3.3; 8.0] | 5.6 [-0.4; 13.3] | 84.0 [74.0; 91.0] |
| Narva linn | Estonia | 2009-2015 | 5993 | 50.6 [38.6; 64.0] | 12.9 [8.7; 18.5] | 7.1 [4.4; 10.9] | 9.0 [6.3; 13.7] | 5.5 [-0.4; 13.3] | 85.0 [75.0; 92.0] |
| Tallinn | Estonia | 2005-2015 | 52878 | 42.5 [30.7; 54.5] | 17.3 [10.3; 27.6] | NA | 21.1 [13.6; 29.7] | 6.4 [0.7; 13.7] | 84.0 [74.0; 91.0] |
| Tartu linn | Estonia | 2008-2015 | 8973 | 47.5 [33.9; 61.2] | 14.8 [10.1; 21.8] | 7.8 [4.3; 12.7] | 10.4 [7.2; 15.4] | 6.6 [0.8; 14] | 84.0 [76.0; 91.0] |
| Bordeaux | France | 2000-2010 | 53219 | 67.4 [47.5; 87.2] | 17.9 [13.8; 23.8] | NA | NA | 13.8 [9.2; 18.7] | 77.0 [68.0; 85.0] |
| Clermont Ferrand | France | 2000-2010 | 19040 | 73.3 [54.4; 91.3] | 16.8 [12.1; 23.2] | NA | NA | 12.3 [6.8; 17.6] | 72.0 [65.0; 80.0] |
| Dijon | France | 2000-2010 | 18786 | 66.3 [44.4; 87.5] | 17.2 [12.3; 23.5] | NA | NA | 11.6 [5.6; 17.1] | 79.0 [69.0; 87.0] |
| Grenoble | France | 2000-2010 | 32738 | 66.1 [35.1; 92.8] | 19.4 [13.2; 27.3] | NA | NA | 12.7 [5.8; 18.4] | 78.0 [70.0; 85.0] |
| Le Havre | France | 2000-2010 | 24323 | 67.3 [53.7; 80.0] | 17.5 [14.0; 23.0] | NA | NA | 11.6 [7.7; 15.9] | 82.0 [75.0; 88.0] |
| Lille | France | 2000-2010 | 90900 | 57.6 [38.1; 74.9] | 19.0 [14.5; 25.2] | NA | NA | 11.3 [6.4; 16] | 81.0 [73.0; 88.0] |
| Lens-Douai | France | 2000-2010 | 36686 | 57.7 [39.1; 75.0] | 18.0 [13.5; 24.5] | NA | NA | 11.3 [6.4; 16] | 81.0 [73.0; 88.0] |
| Lyon | France | 2000-2010 | 77106 | 66.6 [40.0; 91.0] | 19.0 [13.8; 26.5] | NA | NA | 13.1 [7; 18.9] | 71.0 [61.0; 81.0] |
| Montpellier | France | 2000-2010 | 26978 | 81.3 [61.2; 100.8] | 19.8 [13.2; 27.0] | NA | NA | 15.2 [10.1; 20.9] | 68.0 [55.0; 80.0] |
| Marseille | France | 2000-2010 | 94792 | 79.4 [53.6; 101.4] | 25.7 [19.3; 33.3] | NA | NA | 15.5 [10; 21.5] | 66.0 [56.0; 75.0] |

| City | Country | Period | N deaths | Ozone (µg/m³) Median [IQR] | $PM_{10}$ (µg/m³) Median [IQR] | $PM_{2.5}$ (µg/m³) Median [IQR] | $NO_2$ (µg/m³) Median [IQR] | Mean temperature (˚C) Median [IQR] | Relative humidity (%) Median [IQR] |
|---|---|---|---|---|---|---|---|---|---|
| Nice | France | 2000-2010 | 51959 | 89.0 [58.4; 112.2] | 23.0 [18.0; 29.1] | NA | NA | 15.9 [11.2; 21.4] | 69.0 [60.0; 76.0] |
| Nancy | France | 2000-2010 | 28945 | 61.6 [44.7; 81.9] | 17.7 [12.9; 25.3] | NA | NA | 11.2 [5.4; 16.6] | 78.0 [70.0; 86.0] |
| Nantes | France | 2000-2010 | 43547 | 69.0 [53.4; 85.0] | 15.2 [12.0; 19.3] | NA | NA | 12.6 [8.5; 17] | 81.0 [73.0; 88.0] |
| Paris | France | 2000-2010 | 455460 | 56.2 [35.2; 75.8] | 19.3 [14.9; 25.2] | NA | NA | 12.6 [7.7; 17.6] | 73.0 [64.0; 81.0] |
| Rennes | France | 2000-2010 | 16600 | 65.0 [50.7; 79.9] | 15.1 [11.4; 20.0] | NA | NA | 12.3 [8.1; 16.8] | 80.0 [73.0; 86.0] |
| Rouen | France | 2000-2010 | 41927 | 62.3 [45.9; 78.4] | 18.0 [14.5; 23.5] | NA | NA | 10.8 [6.2; 15.4] | 83.0 [75.0; 90.0] |
| Strasbourg | France | 2000-2010 | 34874 | 60.1 [34.9; 85.4] | 18.0 [13.0; 25.5] | NA | NA | 11.4 [5.4; 17.3] | 78.0 [70.0; 86.0] |
| Toulouse | France | 2000-2010 | 49675 | 73.5 [52.7; 93.0] | 19.5 [14.5; 25.5] | NA | NA | 13.6 [8.6; 19.3] | 75.0 [65.0; 83.0] |
| Berlin | Germany | 1993-2015 | 811051 | 55.1 [33.2; 79.5] | 21.6 [15.8; 30.6] | 14.4 [10.3; 21.7] | 27.9 [21.2; 35.5] | 10.4 [4.2; 16.5] | NA |
| Bremen | Germany | 1993-2015 | 150608 | 55.6 [37.0; 75.2] | 17.3 [13.1; 23.4] | 11.6 [7.7; 18.8] | 22.8 [16.3; 30.2] | 10 [4.8; 15] | NA |
| Dresden | Germany | 1993-2015 | 125866 | 62.2 [41.9; 85.5] | 20.9 [13.7; 31.5] | 11.7 [7.1; 20.2] | 26.5 [19.1; 34.9] | 9.9 [3.6; 15.9] | NA |
| Dortmund | Germany | 1993-2015 | 155233 | 52.9 [34.2; 74.6] | 22.1 [16.1; 31.3] | 13.4 [9.5; 20.4] | 31.7 [23.9; 40.8] | 10.8 [5.7; 15.6] | NA |
| Duesseldorf | Germany | 1993-2015 | 160069 | 53.6 [32.5; 73.4] | 19.7 [15.2; 26.8] | 12.0 [8.2; 18.8] | 29.9 [21.2; 39.5] | 11.1 [6; 16] | NA |
| Frankfurt | Germany | 1993-2015 | 168417 | 51.4 [26.9; 76.7] | 21.2 [15.2; 30.2] | 12.9 [9.2; 19.6] | 42.1 [32.3; 51.8] | 11.2 [5.3; 16.9] | NA |
| Hamburg | Germany | 1993-2015 | 445338 | 54.2 [33.5; 72.7] | 22.6 [16.3; 31.6] | 12.7 [8.6; 18.7] | 30.5 [22.7; 39.3] | 10.2 [4.9; 15.3] | NA |
| Hannover | Germany | 1993-2015 | 279125 | 61.3 [42.2; 80.7] | 18.2 [13.0; 26.9] | 9.9 [6.4; 15.8] | 23.6 [16.2; 32.1] | 10.2 [4.8; 15.3] | NA |
| Koeln | Germany | 1993-2015 | 229457 | 54.8 [34.0; 75.9] | 19.4 [13.5; 27.7] | NA | 30.9 [21.7; 39.9] | 10.9 [5.6; 15.8] | NA |
| Leipzig | Germany | 1994-2015 | 146172 | 64.4 [44.2; 86.4] | 18.1 [12.8; 26.0] | 10.1 [6.3; 17.4] | 19.7 [14.3; 26.9] | 10.3 [4.3; 16.1] | NA |
| Muenchen | Germany | 1993-2015 | 290962 | 60.0 [36.0; 83.4] | 20.6 [13.7; 30.7] | 11.6 [7.6; 17.0] | 36.5 [28.2; 46.2] | 10.3 [3.9; 16.2] | NA |
| Stuttgart | Germany | 1993-2015 | 136878 | 60.2 [34.5; 86.5] | 19.0 [13.0; 28.0] | 11.4 [7.5; 18.0] | 33.7 [24.9; 43.2] | 11 [5; 16.6] | NA |
| Athens | Greece | 2001-2010 | 287969 | 75.1 [52.8; 97.5] | 39.5 [29.5; 53.1] | 20.3 [15.0; 26.4] | 50.2 [39.6; 61.6] | 17.9 [12.9; 24.9] | 66.0 [54.0; 75.4] |
| Cagliari | Italy | 2006-2010 | 6440 | 58.9 [48.5; 71.4] | 30.6 [24.1; 38.8] | NA | NA | 16.7 [12.4; 22.4] | NA |
| Florence | Italy | 2006-2010 | 19609 | 76.3 [49.2; 104.3] | 29.8 [23.5; 38.9] | NA | NA | 15.1 [9.7; 21.5] | NA |
| Frosinone | Italy | 2006-2015 | 3787 | 87.1 [63.8; 110.4] | 32.0 [23.0; 59.0] | NA | NA | 15.4 [9.1; 22] | NA |

| City | Country | Period | N deaths | Ozone (µg/m³) Median [IQR] | $PM_{10}$ (µg/m³) Median [IQR] | $PM_{2.5}$ (µg/m³) Median [IQR] | $NO_2$ (µg/m³) Median [IQR] | Mean temperature (˚C) Median [IQR] | Relative humidity (%) Median [IQR] |
|---|---|---|---|---|---|---|---|---|---|
| Genoa | Italy | 2006-2010 | 37943 | 77.5 [57.3; 100.7] | 27.0 [21.0; 34.6] | NA | NA | 16.3 [11.4; 21.5] | NA |
| Latina | Italy | 2006-2015 | 8661 | 78.5 [58.6; 96.1] | 26.0 [20.0; 35.0] | NA | NA | 16.9 [11.5; 23.1] | NA |
| Milan | Italy | 2006-2010 | 53837 | 55.0 [18.7; 92.2] | 36.5 [25.7; 60.6] | NA | NA | 14.1 [7.2; 21.1] | NA |
| Rieti | Italy | 2008-2015 | 3358 | 77.2 [51.1; 100.4] | 20.0 [14.0; 28.2] | NA | NA | 17 [10.3; 23.5] | NA |
| Rome | Italy | 2006-2015 | 226662 | 75.1 [44.2; 98.1] | 28.7 [22.0; 38.0] | NA | NA | 15.5 [10.4; 21.9] | NA |
| Trieste | Italy | 2006-2010 | 13124 | 81.6 [62.9; 99.8] | 23.2 [15.6; 32.2] | NA | NA | 15.6 [9.4; 21.7] | NA |
| Aikita | Japan | 2011-2015 | 17222 | 81.3 [70.3; 98.1] | 12.8 [9.5; 18.5] | 11.4 [8.1; 17.0] | 11.8 [9.0; 15.7] | 12.3 [3.3; 20.6] | 73.0 [66.0; 80.0] |
| Aomori | Japan | 2011-2015 | 17107 | 74.7 [63.7; 88.4] | 16.0 [12.7; 21.3] | 10.6 [7.7; 14.9] | 11.4 [8.1; 17.9] | 11.2 [2; 18.9] | 77.0 [70.0; 83.0] |
| Chiba | Japan | 2011-2015 | 38145 | 76.4 [59.8; 97.9] | 16.9 [11.9; 24.4] | 11.9 [7.7; 17.8] | 23.4 [16.5; 33.9] | 17 [8.9; 22.7] | 69.0 [53.0; 77.0] |
| Fukushima | Japan | 2011-2015 | 15116 | 74.0 [60.8; 92.1] | 15.3 [11.2; 21.2] | 10.0 [6.1; 15.2] | 15.5 [10.7; 22.4] | 14.1 [4.8; 21.4] | 70.0 [61.0; 78.0] |
| Fukuoka | Japan | 2011-2015 | 54231 | 81.7 [64.4; 101.5] | 19.9 [14.1; 28.0] | 16.2 [11.0; 22.9] | 21.2 [15.2; 29.4] | 18 [10; 23.6] | 68.0 [59.0; 78.0] |
| Fukui | Japan | 2011-2015 | 13479 | 85.2 [69.8; 104.8] | 17.2 [11.7; 25.1] | 13.4 [8.9; 19.1] | 10.7 [7.7; 14.9] | 15.4 [6.3; 22.6] | 76.0 [68.0; 83.0] |
| Gifu | Japan | 2011-2015 | 20852 | 82.3 [63.4; 104.5] | 12.7 [7.9; 19.6] | 12.5 [8.2; 17.8] | 17.8 [13.1; 23.1] | 16.9 [7.9; 23.8] | 65.0 [57.0; 74.0] |
| hiroshima | Japan | 2011-2015 | 48257 | 79.9 [62.2; 101.8] | 22.3 [16.2; 30.0] | 15.6 [10.2; 22.4] | 22.0 [16.1; 29.4] | 16.9 [8.3; 23.7] | 66.0 [60.0; 73.0] |
| Kagoshima | Japan | 2011-2015 | 28646 | 70.2 [53.1; 86.5] | 22.6 [16.8; 30.1] | 15.4 [11.0; 22.0] | 12.6 [8.7; 17.2] | 19.5 [12.2; 24.8] | 73.0 [64.0; 80.0] |
| Kumamoto | Japan | 2011-2015 | 32357 | 82.1 [63.1; 103.0] | 22.5 [15.6; 30.7] | 16.3 [9.3; 22.9] | 17.3 [11.3; 25.5] | 18 [9.6; 24] | 70.0 [62.0; 78.0] |
| Kanazawa | Japan | 2011-2015 | 21061 | 87.7 [73.1; 104.0] | 13.7 [9.0; 21.1] | 10.2 [6.8; 15.2] | 13.1 [9.9; 17.9] | 15.6 [6.5; 22.6] | 71.0 [64.0; 78.0] |
| Kobe | Japan | 2011-2015 | 73782 | 77.5 [61.3; 98.9] | 15.9 [11.2; 23.6] | 12.3 [8.4; 18.2] | 25.9 [18.6; 35.2] | 17.8 [9.1; 24] | 63.0 [56.0; 72.0] |
| Kochi | Japan | 2011-2015 | 17800 | 81.1 [64.6; 98.4] | 13.9 [9.5; 20.9] | 13.0 [9.4; 18.8] | 11.2 [8.3; 15.4] | 18.1 [9.9; 23.6] | 69.0 [59.0; 80.0] |
| Kofu | Japan | 2011-2015 | 10710 | 80.8 [64.7; 104.2] | 18.5 [13.0; 25.5] | 10.7 [6.8; 16.2] | 18.8 [14.0; 29.2] | 15.7 [6.9; 22.8] | 64.0 [53.0; 72.0] |
| Kyoto | Japan | 2011-2015 | 69191 | 79.9 [62.7; 100.0] | 14.0 [9.5; 20.4] | 12.5 [8.8; 18.4] | 22.4 [16.5; 30.3] | 16.9 [7.8; 23.7] | 66.0 [60.0; 73.0] |
| Matsue | Japan | 2011-2015 | 11146 | 91.4 [77.4; 109.5] | 12.2 [7.7; 18.6] | 12.4 [7.9; 18.1] | 4.6 [3.3; 6.4] | 15.6 [7.5; 22.2] | 77.0 [70.0; 83.0] |
| Maebashi | Japan | 2011-2015 | 17449 | 84.3 [68.3; 111.0] | 12.6 [6.1; 20.7] | 14.0 [8.0; 20.4] | 14.0 [9.4; 19.4] | 15.6 [6.7; 22.5] | 59.0 [49.0; 71.0] |
| Mito | Japan | 2011-2015 | 12662 | 81.1 [65.7; 101.2] | 18.4 [13.7; 24.9] | 10.4 [7.0; 15.2] | 12.3 [9.4; 17.3] | 14.9 [6.4; 21.2] | 75.0 [63.0; 83.0] |

| City | Country | Period | N deaths | Ozone (μg/m³) Median [IQR] | $PM_{10}$ (μg/m³) Median [IQR] | $PM_{2.5}$ (μg/m³) Median [IQR] | $NO_2$ (μg/m³) Median [IQR] | Mean temperature (˚C) Median [IQR] | Relative humidity (%) Median [IQR] |
|---|---|---|---|---|---|---|---|---|---|
| Morioka | Japan | 2011-2015 | 14084 | 70.8 [58.3; 87.2] | 10.7 [6.5; 16.8] | 11.6 [7.9; 16.6] | 13.2 [9.0; 22.0] | 10.9 [1.6; 19.5] | 74.0 [67.0; 82.0] |
| Matsuyama | Japan | 2011-2015 | 25345 | 73.8 [59.3; 93.8] | 20.4 [14.2; 28.5] | 16.6 [11.5; 22.8] | 20.0 [14.9; 26.3] | 17.4 [9; 23.3] | 66.0 [58.0; 75.0] |
| Nagano | Japan | 2011-2015 | 19818 | 77.0 [62.9; 95.7] | 13.8 [8.9; 20.6] | 10.2 [6.3; 15.4] | 13.4 [9.8; 20.8] | 12.8 [3.1; 21] | 74.0 [66.0; 80.0] |
| Nagoya | Japan | 2011-2015 | 100810 | 79.5 [60.6; 102.7] | 19.8 [13.4; 27.4] | 14.1 [9.6; 19.7] | 28.1 [20.4; 39.0] | 16.9 [8.1; 23.7] | 66.0 [57.0; 74.0] |
| Naha | Japan | 2011-2015 | 12468 | 69.0 [32.5; 83.3] | 19.0 [14.3; 27.1] | 8.8 [6.3; 12.7] | 10.0 [5.8; 16.5] | 23.6 [19.2; 27.8] | 76.0 [66.0; 82.0] |
| Nara | Japan | 2011-2015 | 16901 | 77.9 [63.2; 98.7] | 15.4 [10.7; 21.6] | 11.7 [7.5; 17.3] | 15.9 [11.2; 22.7] | 15.8 [6.9; 22.5] | 73.0 [66.0; 80.0] |
| Nagasaki | Japan | 2011-2015 | 24586 | 75.9 [61.1; 94.1] | 20.1 [14.8; 28.0] | 14.4 [9.4; 20.2] | 13.7 [10.3; 17.9] | 18 [10.3; 23.5] | 72.0 [63.0; 81.0] |
| Niigata | Japan | 2011-2015 | 40382 | 82.6 [71.2; 98.1] | 16.3 [12.7; 21.9] | 10.5 [7.0; 15.5] | 12.3 [9.0; 16.9] | 14.2 [5.3; 22.1] | 72.0 [65.0; 79.0] |
| Oita | Japan | 2011-2015 | 19860 | 72.0 [57.7; 89.1] | 16.4 [11.6; 23.0] | 15.1 [10.0; 21.8] | 13.9 [10.6; 18.3] | 17.2 [9.3; 23.1] | 69.0 [60.0; 78.0] |
| Okayama | Japan | 2011-2015 | 31950 | 76.0 [60.0; 96.5] | 19.4 [13.6; 28.5] | 15.0 [10.6; 21.5] | 21.9 [16.0; 29.0] | 16.8 [7.8; 23.6] | 65.0 [58.0; 74.0] |
| Osaka | Japan | 2011-2015 | 135736 | 74.9 [56.8; 96.8] | 19.6 [14.3; 27.7] | 15.6 [10.8; 21.8] | 36.5 [27.8; 49.0] | 17.6 [8.9; 24] | 63.0 [55.0; 71.0] |
| Otsu | Japan | 2011-2015 | 13929 | 81.9 [65.6; 102.5] | 16.1 [11.5; 22.5] | 10.8 [6.7; 16.3] | 15.8 [11.4; 22.3] | 15.8 [7; 22.8] | 72.0 [67.0; 80.0] |
| Saga | Japan | 2011-2015 | 12400 | 83.4 [66.4; 104.6] | 16.3 [11.3; 23.2] | 13.2 [8.4; 20.0] | 9.3 [7.1; 13.2] | 17.7 [9.2; 23.8] | 70.0 [63.0; 79.0] |
| Saitama | Japan | 2011-2015 | 47101 | 77.7 [59.2; 101.5] | 18.7 [12.9; 26.7] | 12.6 [8.1; 18.4] | 27.8 [20.3; 37.9] | 16.2 [7.1; 22.7] | 65.0 [51.0; 76.0] |
| Sendai | Japan | 2011-2015 | 40680 | 75.1 [62.5; 93.6] | 14.0 [9.8; 21.1] | 10.5 [6.6; 16.3] | 17.0 [12.0; 24.1] | 13.6 [4.7; 20.3] | 71.0 [61.0; 81.0] |
| Shizuoka | Japan | 2011-2015 | 37343 | 82.1 [64.0; 104.7] | 17.3 [12.0; 23.7] | 10.7 [7.4; 15.7] | 19.9 [15.1; 25.2] | 17.6 [10; 23.3] | 69.0 [57.0; 77.0] |
| Sapporo | Japan | 2011-2015 | 84189 | 64.3 [53.4; 81.4] | 10.0 [7.2; 14.1] | 8.8 [6.0; 13.0] | 20.0 [13.9; 31.2] | 9.7 [0.5; 18.3] | 70.0 [62.0; 76.0] |
| Takamatsu | Japan | 2011-2015 | 20843 | 78.5 [63.0; 94.5] | 19.9 [13.6; 29.1] | 15.2 [10.2; 22.1] | 19.4 [13.4; 28.8] | 17.3 [8.6; 23.8] | 66.0 [58.0; 75.0] |
| Tokushima | Japan | 2011-2015 | 13790 | 80.3 [64.7; 103.5] | 16.8 [11.7; 24.3] | 12.5 [8.2; 18.5] | 13.3 [10.1; 17.6] | 17.5 [9; 23.5] | 68.0 [58.0; 76.0] |
| Tokyo | Japan | 2011-2015 | 374187 | 71.0 [53.8; 92.3] | 19.1 [13.5; 26.3] | 13.9 [9.8; 19.9] | 35.1 [26.0; 47.0] | 17.4 [8.9; 23.1] | 65.0 [49.0; 74.0] |
| Toyama | Japan | 2011-2015 | 22126 | 84.2 [71.2; 102.6] | 12.6 [7.6; 19.4] | 9.4 [5.9; 14.0] | 11.9 [8.7; 16.4] | 15.2 [5.8; 22.4] | 76.0 [67.0; 84.0] |
| Tsu | Japan | 2011-2015 | 14919 | 85.3 [68.0; 106.3] | 21.5 [15.8; 28.9] | 13.0 [8.6; 18.6] | 13.8 [9.8; 19.7] | 16.7 [8.3; 23.5] | 67.0 [58.0; 77.0] |
| Utsunomiya | Japan | 2011-2015 | 21851 | 79.9 [60.8; 104.7] | 17.4 [12.5; 24.6] | 11.9 [8.0; 17.3] | 21.0 [16.0; 29.3] | 15.2 [6; 21.8] | 68.0 [58.0; 78.0] |
| Wakayama | Japan | 2011-2015 | 21449 | 80.9 [65.3; 103.0] | 19.5 [14.5; 26.3] | 14.2 [9.7; 19.9] | 16.7 [13.0; 21.8] | 17.7 [9.1; 23.7] | 65.0 [57.0; 73.0] |

| City | Country | Period | N deaths | Ozone (μg/m$^3$) Median [IQR] | $PM_{10}$ (μg/m$^3$) Median [IQR] | $PM_{2.5}$ (μg/m$^3$) Median [IQR] | $NO_2$ (μg/m$^3$) Median [IQR] | Mean temperature (˚C) Median [IQR] | Relative humidity (%) Median [IQR] |
|---|---|---|---|---|---|---|---|---|---|
| Yokohama | Japan | 2011-2015 | 146525 | 69.8 [51.6; 96.9] | 21.6 [15.8; 29.2] | 13.3 [9.1; 19.3] | 29.8 [20.4; 40.4] | 17.2 [9; 22.7] | 70.0 [54.0; 79.0] |
| Yamaguchi | Japan | 2011-2015 | 10481 | 77.8 [61.5; 98.5] | 10.7 [6.6; 17.2] | 13.0 [8.3; 18.8] | 16.4 [11.8; 21.8] | 16.3 [7.7; 22.8] | 73.0 [66.0; 80.0] |
| Yamagata | Japan | 2011-2015 | 13266 | 77.7 [63.4; 95.1] | 14.6 [9.8; 21.5] | 10.7 [6.8; 16.6] | 15.6 [11.2; 24.4] | 12.3 [2.5; 20.9] | 74.0 [66.0; 81.0] |
| Busan | South Korea | 1999-2015 | 323037 | 64.5 [49.9; 82.7] | 47.9 [36.3; 64.5] | NA | 40.7 [31.0; 52.6] | 16 [8.3; 21.6] | 64.6 [48.1; 77.5] |
| Daegu | South Korea | 1999-2015 | 198083 | 61.3 [43.8; 87.4] | 48.0 [35.0; 64.9] | NA | 43.2 [32.0; 57.9] | 15.8 [6.3; 22.8] | 58.9 [45.1; 69.9] |
| Daejeon | South Korea | 1999-2015 | 102449 | 59.2 [40.4; 84.0] | 40.9 [28.7; 56.8] | NA | 36.6 [27.0; 50.7] | 14.4 [4.5; 22.2] | 68.0 [57.3; 77.1] |
| Gwangju | South Korea | 1999-2015 | 104787 | 60.8 [43.0; 84.2] | 41.2 [29.8; 56.9] | NA | 36.9 [27.6; 49.1] | 15.4 [6; 22.6] | 67.5 [58.0; 76.0] |
| Incheon | South Korea | 1999-2015 | 195684 | 57.1 [40.7; 78.5] | 50.0 [36.0; 68.2] | NA | 50.6 [37.9; 66.7] | 14 [4.3; 21.6] | 69.8 [57.9; 80.9] |
| Seoul | South Korea | 1999-2015 | 666658 | 49.6 [31.5; 72.9] | 49.9 [34.1; 71.3] | NA | 65.7 [50.4; 84.5] | 14.4 [4; 22.3] | 61.5 [50.5; 72.0] |
| Ulsan | South Korea | 1999-2015 | 71501 | 64.2 [49.6; 83.9] | 43.8 [33.2; 58.3] | NA | 37.5 [29.2; 48.1] | 15.5 [7; 21.6] | 65.0 [49.0; 75.6] |
| Guadalajara | Mexico | 2000-2012 | 267190 | 113.7 [88.2; 149.0] | 46.8 [33.1; 62.4] | 24.1 [18.5; 32.3] | NA | 21.3 [19.4; 22.9] | 59.1 [42.5; 75.1] |
| Leon | Mexico | 2005-2012 | 54605 | 107.8 [84.3; 129.4] | 55.5 [40.3; 76.0] | NA | NA | 20 [17; 22] | 47.0 [35.0; 62.0] |
| Monterrey | Mexico | 2000-2012 | 218397 | 96.0 [72.5; 119.6] | 74.1 [57.7; 95.1] | 26.3 [20.1; 33.7] | NA | 23.2 [18.3; 26.8] | 63.8 [53.3; 73.1] |
| Puebla-Tlaxcala | Mexico | 2000-2011 | 143078 | 92.1 [68.6; 121.5] | 34.7 [26.1; 49.6] | 17.7 [14.4; 23.0] | NA | 16.9 [15.1; 18.1] | 63.0 [54.8; 70.6] |
| Tijuana | Mexico | 2000-2012 | 73801 | 76.4 [64.7; 90.2] | 60.5 [46.0; 84.3] | NA | NA | 18 [14; 20] | 76.0 [69.0; 80.0] |
| Toluca de Lerdo | Mexico | 2000-2012 | 98959 | 111.7 [90.2; 133.3] | 60.2 [38.8; 87.3] | 31.9 [24.1; 39.0] | NA | 14.4 [12.7; 15.5] | 63.0 [53.0; 71.7] |
| Valley of Mexico | Mexico | 2000-2012 | 1162283 | 164.6 [127.4; 201.9] | 49.3 [35.3; 65.7] | 24.9 [17.9; 32.6] | NA | 16.5 [14.9; 17.8] | 54.0 [42.5; 65.0] |
| Lisboa | Portugal | 1997-2012 | 337920 | 66.8 [52.2; 82.2] | 24.4 [17.1; 35.5] | 11.0 [7.1; 18.0] | 29.2 [18.0; 43.1] | 17.1 [13.3; 20.9] | NA |
| Porto | Portugal | 1999-2012 | 199038 | 61.6 [48.2; 76.2] | 30.1 [19.9; 46.1] | NA | 24.7 [16.5; 34.8] | 15.2 [11.8; 18.4] | NA |
| Johannesburg | South Africa | 2004-2013 | 344680 | 65.6 [47.5; 88.7] | 50.4 [36.5; 70.5] | 32.8 [23.1; 47.4] | NA | 17.6 [13.5; 20.1] | NA |
| eThekwini | South Africa | 2004-2013 | 354160 | 55.2 [41.6; 71.1] | NA | NA | NA | 21.6 [18.9; 24.2] | NA |
| Gert Sibande DM | South Africa | 2008-2013 | 73257 | 84.3 [67.0; 101.6] | 46.5 [27.3; 75.8] | 25.5 [15.7; 38.6] | NA | 16.8 [13.3; 19.7] | NA |
| Nkangala DM | South Africa | 2008-2013 | 74552 | 68.9 [51.1; 96.5] | 36.2 [20.8; 61.8] | 17.8 [11.5; 28.2] | NA | 17 [11.8; 20.3] | NA |
| Sedibeng DM | South Africa | 2007-2013 | 77829 | 73.5 [57.5; 89.7] | 59.2 [40.8; 84.5] | 31.9 [22.5; 44.2] | NA | 18.6 [13.3; 21.5] | NA |

| City | Country | Period | N deaths | Ozone (μg/m$^3$) Median [IQR] | $PM_{10}$ (μg/m$^3$) Median [IQR] | $PM_{2.5}$ (μg/m$^3$) Median [IQR] | $NO_2$ (μg/m$^3$) Median [IQR] | Mean temperature (˚C) Median [IQR] | Relative humidity (%) Median [IQR] |
|---|---|---|---|---|---|---|---|---|---|
| A Coruna | Spain | 2005-2014 | 22778 | 56.9 [35.1; 75.1] | 25.0 [19.5; 32.0] | 9.8 [4.2; 13.7] | 26.6 [18.8; 37.2] | 15.2 [12.2; 18.4] | NA |
| Albacete | Spain | 2004-2014 | 12068 | 85.5 [64.2; 103.0] | 36.3 [27.5; 47.1] | 11.9 [8.2; 16.7] | 13.0 [8.4; 19.1] | 14.2 [8.2; 21.7] | NA |
| Alicante | Spain | 2004-2014 | 27376 | 76.1 [61.3; 88.0] | 19.0 [14.6; 22.9] | NA | 30.1 [24.3; 37.1] | 18.6 [13.6; 23.7] | NA |
| Almeria | Spain | 2004-2014 | 14734 | 79.7 [66.9; 92.3] | 23.5 [19.2; 30.5] | NA | 21.3 [16.4; 27.4] | 18.5 [14.4; 23.8] | NA |
| Avila | Spain | 2004-2014 | 4977 | 69.8 [59.4; 81.6] | 21.5 [18.1; 25.1] | NA | 21.6 [17.3; 26.1] | 11.1 [5.8; 17.8] | NA |
| Badajoz | Spain | 2005-2014 | 10466 | 51.8 [36.2; 67.2] | 28.1 [14.3; 97.0] | NA | 7.7 [4.4; 12.4] | 17 [11.6; 23.2] | NA |
| Bilbao | Spain | 2004-2014 | 38517 | 56.8 [42.2; 68.4] | 35.3 [30.3; 41.7] | 11.0 [6.9; 16.2] | 36.1 [28.3; 44.8] | 15.2 [10.9; 19.3] | NA |
| Barcelona | Spain | 2004-2014 | 167513 | 60.2 [40.6; 75.3] | 28.8 [21.0; 40.2] | 18.4 [13.2; 26.0] | 44.2 [34.4; 54.9] | 16.6 [11.6; 22.4] | NA |
| Burgos | Spain | 2004-2014 | 16854 | 75.8 [60.2; 92.0] | 25.6 [20.2; 32.5] | 7.6 [5.0; 12.2] | 23.0 [17.2; 31.2] | 10.6 [5.4; 16.6] | NA |
| Cadiz | Spain | 2004-2014 | 13347 | 81.0 [65.9; 94.1] | 33.3 [26.3; 44.5] | NA | 17.6 [11.8; 25.0] | 18.4 [14.6; 23] | NA |
| Caceres | Spain | 2005-2014 | 6535 | 64.2 [56.0; 72.1] | 33.9 [17.8; 89.7] | 5.9 [4.2; 8.2] | 11.3 [6.5; 17.4] | 15.8 [10.4; 22.7] | NA |
| Ciudad Real | Spain | 2008-2014 | 3863 | 83.4 [66.2; 99.4] | 19.3 [12.8; 27.8] | NA | 8.4 [4.6; 14.3] | 15.2 [9.1; 23.2] | NA |
| Ceuta | Spain | 2004-2014 | 5421 | 77.8 [58.8; 97.3] | NA | NA | NA | 18.1 [14.9; 22.6] | NA |
| Cordoba | Spain | 2004-2014 | 27705 | 77.3 [64.5; 89.3] | 42.7 [36.1; 51.3] | NA | 29.6 [25.0; 34.7] | 18 [12.2; 25] | NA |
| Castellon | Spain | 2004-2014 | 13790 | 64.7 [56.2; 72.0] | 30.1 [25.5; 35.1] | NA | 39.3 [33.4; 46.2] | 18 [13; 23.5] | NA |
| Cuenca | Spain | 2008-2014 | 3435 | 81.8 [60.8; 99.8] | 25.5 [17.2; 34.2] | NA | 16.2 [8.7; 23.9] | 13.2 [7.4; 20.5] | NA |
| Guadalajara | Spain | 2004-2014 | 5856 | 83.1 [56.6; 107.2] | 22.5 [14.9; 32.6] | NA | 24.7 [15.2; 36.0] | 12.2 [6.8; 19.4] | NA |
| Granada | Spain | 2004-2014 | 23395 | 72.0 [56.5; 85.8] | 36.0 [26.6; 46.0] | NA | 38.8 [30.3; 48.2] | 15.1 [9.1; 22.3] | NA |
| Huesca | Spain | 2004-2014 | 5372 | 81.2 [62.5; 99.8] | 17.3 [12.3; 22.9] | 10.6 [8.1; 14.3] | 18.1 [12.9; 26.1] | 14.3 [8.4; 20.8] | NA |
| Jaen | Spain | 2004-2014 | 9584 | 89.5 [69.1; 107.7] | 36.1 [24.9; 50.6] | NA | 17.9 [13.2; 24.9] | 16.4 [10.8; 23.6] | NA |
| Leon | Spain | 2004-2014 | 15023 | 60.8 [43.7; 77.5] | 22.3 [16.8; 29.5] | 5.1 [3.2; 7.8] | 27.5 [20.7; 36.7] | 10.8 [5.5; 16.7] | NA |
| Lleida | Spain | 2004-2013 | 10700 | 71.5 [43.8; 92.1] | NA | NA | 25.2 [18.0; 33.9] | 15.4 [8.8; 22] | NA |
| Lugo | Spain | 2005-2014 | 8745 | 57.1 [41.8; 73.8] | 20.3 [15.3; 24.8] | NA | 22.2 [18.1; 26.6] | 12.3 [8.2; 16.7] | NA |
| Malaga | Spain | 2004-2014 | 48400 | 78.2 [66.3; 89.3] | 24.6 [20.6; 30.0] | NA | 31.8 [26.2; 38.0] | 18.7 [14.4; 23.9] | NA |

| City | Country | Period | N deaths | Ozone (µg/m³) Median [IQR] | $PM_{10}$ (µg/m³) Median [IQR] | $PM_{2.5}$ (µg/m³) Median [IQR] | $NO_2$ (µg/m³) Median [IQR] | Mean temperature (˚C) Median [IQR] | Relative humidity (%) Median [IQR] |
|---|---|---|---|---|---|---|---|---|---|
| Madrid | Spain | 2004-2014 | 284880 | 58.0 [38.2; 77.1] | 24.1 [16.6; 33.9] | 10.9 [8.2; 14.3] | 43.7 [33.9; 56.4] | 14.6 [8.8; 22.2] | NA |
| Melilla | Spain | 2004-2014 | 4748 | 80.3 [61.2; 104.5] | NA | NA | NA | 18.6 [15; 23.4] | NA |
| Murcia | Spain | 2009-2014 | 17074 | 80.2 [54.9; 98.5] | 22.2 [18.4; 26.6] | NA | 45.1 [39.8; 50.5] | 19.2 [13.6; 25.4] | NA |
| Ourense | Spain | 2004-2014 | 11763 | 65.4 [46.7; 83.9] | 14.1 [10.6; 20.2] | NA | 29.6 [20.7; 40.4] | 15.2 [10.4; 20.3] | NA |
| Oviedo | Spain | 2004-2014 | 23282 | 61.0 [46.8; 73.2] | 27.8 [20.9; 37.9] | 10.4 [7.2; 14.7] | 28.2 [21.2; 38.1] | 13.6 [9.5; 17.5] | NA |
| Palmas Gu Canaria | Spain | 2004-2014 | 31691 | 48.4 [40.0; 59.6] | 24.6 [18.2; 32.6] | 6.5 [5.1; 8.9] | 22.1 [15.9; 30.0] | 21.2 [19; 23.6] | NA |
| Palma Mallorca | Spain | 2004-2014 | 31053 | 69.3 [57.0; 81.1] | 21.4 [16.5; 27.0] | 10.6 [8.3; 13.6] | 25.5 [19.9; 32.1] | 16.6 [11.8; 22.5] | NA |
| Palencia | Spain | 2004-2014 | 8610 | 69.2 [60.5; 78.8] | 26.2 [22.8; 30.4] | NA | 27.6 [23.3; 32.6] | 11.6 [6.1; 17.9] | NA |
| Pamplona | Spain | 2004-2014 | 17757 | 65.2 [50.6; 79.1] | NA | NA | NA | 13.2 [7.8; 18.8] | NA |
| Segovia | Spain | 2004-2014 | 5412 | 73.3 [63.1; 83.1] | 21.2 [16.7; 25.4] | NA | 29.7 [25.4; 34.0] | 12.1 [6.4; 18.8] | NA |
| Salamanca | Spain | 2004-2014 | 16314 | 61.1 [47.8; 74.6] | 20.2 [15.9; 26.0] | 6.7 [5.0; 9.6] | 31.6 [26.1; 38.5] | 12 [6.6; 18.2] | NA |
| San Sebastian | Spain | 2004-2014 | 19051 | 57.5 [44.0; 71.0] | 22.2 [17.1; 29.3] | 10.1 [7.7; 14.0] | 27.8 [20.8; 36.9] | 13.9 [9.8; 17.8] | NA |
| Santander | Spain | 2004-2014 | 19877 | 53.8 [41.6; 66.0] | 26.0 [20.3; 33.1] | NA | 26.6 [20.8; 33.7] | 14.9 [11; 18.8] | NA |
| Soria | Spain | 2004-2014 | 3872 | 68.1 [52.6; 83.0] | 22.4 [14.3; 31.1] | NA | 24.7 [17.4; 32.5] | 10.6 [5.3; 17] | NA |
| Sevilla | Spain | 2004-2014 | 63663 | 76.7 [53.9; 96.1] | 39.3 [31.8; 48.6] | 13.8 [10.7; 18.6] | 31.5 [23.6; 40.5] | 19.2 [13.7; 25.6] | NA |
| Teruel | Spain | 2004-2014 | 3354 | 82.0 [65.2; 97.9] | 18.2 [13.3; 25.1] | NA | 14.3 [9.6; 20.8] | 12.3 [6.2; 19.2] | NA |
| Tenerife | Spain | 2010-2014 | 8541 | 73.9 [63.4; 83.9] | 16.0 [12.0; 22.0] | 6.7 [4.6; 10.6] | 18.2 [12.6; 26.0] | 21.6 [19.2; 24.4] | NA |
| Toledo | Spain | 2004-2014 | 5627 | 84.4 [70.8; 96.4] | 33.8 [29.0; 39.5] | 11.5 [7.8; 16.2] | 24.6 [20.7; 29.4] | 15.3 [9.4; 23.1] | NA |
| Tarragona | Spain | 2004-2014 | 10255 | 78.0 [55.5; 93.5] | NA | NA | 24.6 [17.8; 33.5] | 18 [12.6; 24.1] | NA |
| Vitoria | Spain | 2004-2011 | 13389 | 65.8 [49.9; 81.1] | 20.3 [14.0; 29.7] | 7.6 [5.8; 10.4] | 27.9 [20.0; 36.8] | 11.8 [6.8; 16.6] | NA |
| Valladolid | Spain | 2004-2014 | 29470 | 74.5 [54.3; 91.8] | 17.8 [12.5; 23.9] | 11.3 [7.8; 16.1] | 28.1 [21.2; 36.4] | 12.6 [6.8; 19.1] | NA |
| Valencia | Spain | 2004-2014 | 76883 | 60.1 [43.7; 71.3] | 24.2 [18.0; 32.0] | 14.5 [9.5; 21.0] | 37.9 [29.3; 48.2] | 18.5 [13.8; 23.6] | NA |
| Zamora | Spain | 2004-2014 | 6834 | 69.2 [59.3; 78.6] | 22.6 [19.5; 26.5] | NA | 28.0 [24.1; 32.2] | 12.9 [7.4; 19.4] | NA |
| Zaragoza | Spain | 2004-2014 | 64308 | 48.5 [30.1; 63.6] | 27.5 [18.5; 40.0] | NA | 40.3 [32.1; 48.9] | 15.8 [9.8; 22.4] | NA |

| City | Country | Period | N deaths | Ozone (µg/m³) Median [IQR] | $PM_{10}$ (µg/m³) Median [IQR] | $PM_{2.5}$ (µg/m³) Median [IQR] | $NO_2$ (µg/m³) Median [IQR] | Mean temperature (˚C) Median [IQR] | Relative humidity (%) Median [IQR] |
|---|---|---|---|---|---|---|---|---|---|
| Basel | Switzerland | 1995-2013 | 37607 | 71.7 [44.0; 94.4] | 18.7 [11.9; 28.4] | 13.5 [8.2; 21.5] | 23.7 [14.5; 35.2] | 11.2 [5.1; 16.7] | 75.9 [67.5; 82.8] |
| Bern | Switzerland | 2003-2013 | 15142 | 74.3 [50.3; 97.3] | 26.3 [19.1; 36.7] | 17.4 [12.2; 24.8] | 45.9 [37.5; 55.3] | 9.7 [2.8; 15.6] | 78.6 [70.3; 85.8] |
| Geneve | Switzerland | 1995-2013 | 26306 | 64.9 [39.8; 87.2] | 20.2 [13.3; 29.9] | NA | 36.6 [27.7; 46.6] | 11.3 [4.9; 17] | 72.7 [64.2; 80.9] |
| Lausanne | Switzerland | 1995-2013 | 20810 | 73.5 [50.7; 97.2] | 23.3 [15.7; 34.2] | NA | 44.7 [35.5; 53.4] | 11.5 [5.5; 17.2] | 72.0 [63.6; 79.3] |
| Lugano | Switzerland | 1995-2013 | 28567 | 73.8 [33.5; 112.0] | 24.8 [15.7; 37.6] | 19.3 [11.9; 30.3] | 35.7 [24.2; 49.0] | 12.8 [6.7; 19] | 70.5 [59.7; 80.2] |
| Luzern | Switzerland | 1995-2013 | 15073 | 66.6 [37.2; 92.9] | 18.7 [12.5; 27.4] | NA | 24.2 [18.0; 32.5] | 10.2 [3.7; 16.1] | 78.9 [70.3; 85.7] |
| St. Gallen | Switzerland | 1995-2013 | 13543 | 84.6 [70.0; 105.0] | 15.9 [9.9; 24.5] | NA | 12.2 [8.3; 18.8] | 9.1 [2.7; 14.5] | 77.2 [67.0; 86.0] |
| Zurich | Switzerland | 1995-2013 | 73539 | 73.2 [50.3; 98.5] | 19.9 [13.1; 29.7] | 13.9 [9.3; 21.8] | 33.2 [24.7; 43.3] | 10.1 [3.6; 15.7] | 78.0 [68.3; 85.6] |
| Stockholm | Sweden | 1990-2010 | 201197 | 61.9 [48.9; 76.0] | 12.5 [9.3; 17.9] | 6.6 [4.7; 9.5] | 26.8 [20.0; 34.8] | 6.8 [1.2; 13.9] | 79.6 [68.4; 87.6] |
| Kaohsiung | Taiwan | 2008-2014 | 119890 | 127.1 [88.0; 161.2] | 70.3 [43.7; 95.8] | 40.8 [23.6; 54.6] | 35.9 [25.4; 48.5] | 26.4 [22.4; 28.7] | 75.0 [71.0; 78.0] |
| Taipei | Taiwan | 2008-2014 | 230039 | 98.2 [79.4; 123.5] | 42.1 [31.6; 56.4] | 24.8 [17.4; 34.0] | 40.2 [33.4; 48.3] | 23.4 [18.5; 27.9] | 75.3 [69.0; 81.3] |
| Taichung | Taiwan | 2008-2014 | 93751 | 101.9 [78.9; 131.2] | 51.3 [37.3; 70.0] | 30.3 [20.5; 42.7] | 31.6 [24.0; 40.0] | 24.4 [18.9; 27.9] | 75.5 [71.5; 80.0] |
| Bristol | UK | 1993-2005 | 67017 | 56.0 [40.0; 69.0] | 25.0 [19.0; 34.0] | NA | NA | 11.2 [7.6; 15.4] | 72.1 [62.4; 81.1] |
| Cardiff | UK | 1993-2006 | 45854 | 53.0 [38.0; 67.0] | 27.0 [21.0; 36.0] | NA | NA | 11.3 [7.7; 15.4] | 71.8 [61.9; 81.2] |
| Greater London | UK | 1993-2006 | 835683 | 48.6 [32.3; 63.0] | 24.2 [19.3; 32.3] | NA | NA | 11.2 [7.2; 15.8] | 70.5 [59.6; 80.5] |
| Greater Manchester | UK | 1996-2006 | 171932 | 48.3 [35.7; 60.0] | 22.3 [17.5; 30.2] | NA | NA | 10.3 [6.5; 14.5] | 75.2 [65.1; 84.0] |
| Kingston upon Hull | UK | 1994-2002 | 30888 | 48.0 [35.0; 62.0] | 27.0 [21.0; 35.0] | NA | NA | 10.4 [6.4; 14.5] | 80.7 [71.1; 88.7] |
| Leicester | UK | 1994-2006 | 55224 | 52.0 [36.0; 67.0] | 21.0 [16.0; 28.0] | NA | NA | 9.8 [5.7; 14.1] | NA |
| Liverpool | UK | 1993-2002 | 75858 | 54.0 [37.0; 66.0] | 25.0 [20.0; 35.0] | NA | NA | 10.2 [6.7; 14.2] | 81.1 [73.1; 88.3] |
| Norwich | UK | 1997-2006 | 20082 | 59.0 [41.0; 75.0] | 20.0 [16.0; 26.0] | NA | NA | 10.3 [6.2; 14.8] | 72.3 [62.2; 81.5] |
| Nottingham | UK | 1996-2006 | 56017 | 44.0 [30.0; 59.0] | 23.0 [18.0; 30.0] | NA | NA | 10 [5.9; 14.2] | 77.1 [66.5; 86.7] |
| Sheffield | UK | 1996-2006 | 62836 | 49.5 [35.9; 62.0] | 23.0 [17.0; 33.0] | NA | NA | 10.2 [6.2; 14.5] | 70.7 [60.4; 79.9] |
| Southampton | UK | 1994-2006 | 35500 | 51.0 [37.0; 64.0] | 24.0 [19.0; 32.0] | NA | NA | 11.9 [8.1; 16.1] | 75.2 [64.7; 84.6] |
| The Potteries | UK | 1997-2006 | 39960 | 55.0 [41.0; 69.0] | 21.0 [16.0; 28.0] | NA | NA | 9.6 [5.8; 13.9] | 85.2 [79.8; 92.7] |

| City | Country | Period | N deaths | Ozone (μg/m³) Median [IQR] | $PM_{10}$ (μg/m³) Median [IQR] | $PM_{2.5}$ (μg/m³) Median [IQR] | $NO_2$ (μg/m³) Median [IQR] | Mean temperature (˚C) Median [IQR] | Relative humidity (%) Median [IQR] |
|---|---|---|---|---|---|---|---|---|---|
| Tyneside | UK | 1993-2006 | 98256 | 54.0 [40.0; 68.0] | 20.0 [15.0; 28.0] | NA | NA | 9.9 [6.3; 13.8] | 71.4 [62.4; 80.3] |
| West Midlands | UK | 1993-2006 | 335592 | 54.4 [39.7; 66.9] | 21.5 [16.3; 30.3] | NA | NA | 10 [6; 14.2] | 75.5 [64.6; 85.7] |
| West Yorkshire | UK | 1993-2006 | 142586 | 46.5 [32.2; 59.5] | 25.0 [19.0; 35.0] | NA | NA | 9.6 [5.7; 13.8] | 75.6 [65.6; 84.4] |
| Augusta | USA | 1989-2006 | 30967 | 94.6 [70.6; 118.0] | 21.0 [14.0; 31.0] | 15.7 [10.9; 21.2] | NA | 18.9 [11.8; 24.6] | 68.0 [55.9; 78.7] |
| Akron | USA | 1985-2006 | 107392 | 87.7 [60.3; 116.6] | 21.2 [15.3; 29.8] | 14.1 [9.8; 20.1] | NA | 11.1 [2; 19.1] | 72.0 [63.2; 80.2] |
| Albany | USA | 1987-2006 | 54560 | 65.9 [46.8; 88.0] | 19.7 [14.0; 29.2] | 8.9 [5.6; 14.9] | 27.1 [18.2; 37.8] | 9.8 [1.4; 18.5] | 69.0 [59.9; 78.3] |
| Albuquerque | USA | 1985-2006 | 73279 | 84.3 [64.9; 102.7] | 25.7 [17.9; 35.7] | 5.9 [4.4; 8.1] | 31.4 [22.8; 42.8] | 14.5 [6.3; 22.1] | 38.7 [27.2; 51.8] |
| Allentown | USA | 1985-2006 | 61366 | 78.2 [50.3; 108.4] | 21.1 [14.0; 31.8] | 11.7 [7.4; 18.3] | 30.8 [22.2; 42.0] | 11.7 [3.3; 19.8] | 66.7 [56.8; 76.6] |
| Anaheim | USA | 1985-2006 | 320343 | 77.8 [54.5; 99.4] | 34.0 [24.7; 43.6] | 13.0 [8.9; 19.4] | 54.0 [38.2; 75.8] | 18.8 [15.4; 22] | 63.8 [52.7; 71.0] |
| AnnArbor | USA | 1985-2006 | 33609 | 87.5 [67.8; 109.0] | 15.0 [10.0; 21.0] | 11.5 [7.4; 18.1] | NA | 10.1 [1.3; 18.8] | 71.7 [63.2; 79.7] |
| Annandale | USA | 1985-2006 | 71093 | 67.9 [42.5; 103.8] | 21.0 [14.4; 29.0] | 12.1 [8.3; 17.6] | 36.3 [27.0; 47.4] | 14.6 [6.4; 22.6] | 64.0 [53.2; 74.5] |
| Austin | USA | 1985-2006 | 69427 | 79.4 [62.2; 102.1] | 20.7 [15.5; 27.0] | 9.0 [6.5; 12.5] | 14.8 [6.5; 31.4] | 21.6 [14.8; 26.9] | 66.5 [55.8; 76.3] |
| Atlantic City | USA | 1985-2006 | 49410 | 77.8 [56.1; 105.3] | 27.0 [20.0; 36.0] | 9.1 [6.5; 14.6] | NA | 12.7 [5.1; 20.4] | 71.1 [60.8; 81.4] |
| Atlanta | USA | 1985-2006 | 310249 | 95.5 [68.5; 124.7] | 25.5 [18.2; 34.9] | 16.1 [11.6; 21.5] | 35.6 [26.0; 46.7] | 17.2 [9.9; 23.6] | 69.3 [59.6; 78.2] |
| Atzec | USA | 1997-2006 | 3858 | 94.6 [74.7; 111.5] | 14.2 [10.4; 18.5] | 5.5 [4.3; 7.3] | 18.2 [13.7; 24.6] | 11.8 [3.3; 21.2] | 39.3 [25.4; 55.8] |
| Buffalo | USA | 1985-2006 | 212201 | 68.4 [49.5; 94.1] | 19.4 [13.9; 28.3] | 11.1 [7.2; 17.1] | 33.6 [25.1; 43.5] | 9.3 [1.2; 18] | 72.5 [64.5; 80.4] |
| Bakersfield | USA | 1985-2006 | 88852 | 106.0 [68.1; 143.7] | 36.6 [24.2; 52.5] | 12.6 [8.4; 20.5] | 31.7 [24.7; 42.0] | 17.9 [11.8; 25.2] | 50.3 [37.6; 68.9] |
| Boulder | USA | 1985-2006 | 24614 | 80.4 [57.3; 102.3] | 20.6 [14.7; 28.9] | 7.2 [5.5; 9.7] | NA | 12.2 [4.4; 20.3] | 41.6 [30.9; 57.2] |
| Baltimore | USA | 1985-2006 | 319591 | 68.6 [42.7; 102.0] | 27.2 [18.8; 38.3] | 13.6 [8.9; 19.8] | 45.5 [35.0; 57.4] | 14.1 [5.9; 22.1] | 64.5 [53.2; 75.9] |
| Bangor | USA | 1985-2006 | 26523 | 74.7 [58.6; 91.9] | 16.9 [11.6; 25.0] | 7.9 [5.4; 11.9] | NA | 7.8 [-0.5; 16.4] | 68.6 [58.3; 79.8] |
| Bergen | USA | 1985-2006 | 239023 | 58.7 [35.5; 87.0] | 29.6 [21.0; 40.7] | 10.8 [7.1; 17.6] | 51.4 [38.0; 66.8] | 12.8 [4.5; 20.8] | 62.6 [51.4; 75.1] |
| Burlington | USA | 1985-2006 | 17292 | 78.6 [63.0; 97.0] | 15.4 [10.7; 22.5] | 6.9 [4.4; 11.2] | 29.4 [22.8; 36.9] | 8.6 [-0.2; 17.6] | 67.7 [59.1; 76.5] |
| Birmingham | USA | 1985-2006 | 171109 | 83.5 [59.1; 109.9] | 29.5 [19.2; 43.9] | 14.5 [10.2; 20.9] | 19.7 [14.2; 25.9] | 18.3 [10.8; 24.4] | 68.7 [58.6; 78.3] |

| City | Country | Period | N deaths | Ozone (μg/m³) Median [IQR] | $PM_{10}$ (μg/m³) Median [IQR] | $PM_{2.5}$ (μg/m³) Median [IQR] | $NO_2$ (μg/m³) Median [IQR] | Mean temperature (˚C) Median [IQR] | Relative humidity (%) Median [IQR] |
|---|---|---|---|---|---|---|---|---|---|
| Barnstable | USA | 1987-2006 | 47369 | 87.0 [70.3; 108.8] | NA | NA | 4.1 [1.7; 6.6] | 10.5 [3.9; 18.2] | 74.1 [62.1; 85.0] |
| Brownsville | USA | 1985-2006 | 36059 | 60.0 [42.4; 81.0] | 22.3 [16.2; 31.1] | 9.0 [6.6; 11.8] | NA | 24.3 [19.7; 27.9] | 76.0 [70.6; 82.0] |
| Boston | USA | 1985-2006 | 475683 | 58.1 [39.1; 82.2] | 23.1 [17.1; 31.4] | 10.4 [7.1; 15.5] | 43.8 [33.4; 55.2] | 10.7 [3.6; 18.7] | 66.2 [53.7; 79.3] |
| Baton Rouge | USA | 1985-2006 | 62561 | 73.3 [53.3; 100.2] | 26.4 [20.5; 35.0] | 12.1 [9.1; 16.4] | 24.6 [18.7; 32.8] | 21.1 [14.4; 25.8] | 74.5 [66.0; 82.0] |
| Cedar Rapids | USA | 1985-2006 | 28150 | 63.0 [45.3; 84.4] | 24.3 [17.3; 33.4] | 9.1 [5.8; 14.4] | 6.9 [4.5; 9.3] | 10.3 [0.6; 19.6] | 73.4 [63.7; 82.2] |
| Chicago | USA | 1985-2006 | 1115158 | 56.0 [37.0; 79.5] | 28.7 [20.2; 39.7] | 13.8 [9.2; 19.4] | 44.0 [35.0; 54.3] | 11.4 [2.8; 20.7] | 66.3 [56.8; 76.0] |
| Charlotte | USA | 1985-2006 | 82255 | 100.7 [72.8; 127.1] | 27.1 [19.6; 36.7] | 14.1 [10.0; 19.2] | 30.5 [21.7; 39.9] | 17 [9.4; 23.4] | 64.8 [53.5; 76.0] |
| Charleston SC | USA | 1987-2006 | 45917 | 74.5 [57.1; 95.1] | 18.6 [14.0; 24.2] | 11.0 [7.8; 15.2] | 11.2 [7.0; 17.2] | 19.6 [13.1; 25.1] | 74.1 [64.0; 81.7] |
| Chattanoga | USA | 1985-2006 | 60219 | 90.8 [63.2; 115.9] | 28.9 [20.0; 39.4] | 13.2 [8.9; 18.1] | NA | 16.8 [9; 23.4] | 69.5 [60.2; 78.3] |
| Charlestown WV | USA | 1985-2006 | 49105 | 77.7 [46.6; 108.3] | 22.0 [15.1; 31.1] | 14.5 [10.1; 20.5] | 39.5 [31.6; 48.5] | 14.2 [5.8; 21] | 71.3 [60.8; 80.1] |
| Columbus | USA | 1985-2006 | 159353 | 88.3 [61.2; 113.0] | 25.8 [18.6; 36.4] | 14.6 [10.3; 20.1] | 46.1 [35.0; 59.4] | 12.7 [3.6; 20.7] | 68.5 [60.0; 77.0] |
| Colorado Springs | USA | 1985-2006 | 51338 | 80.1 [61.7; 98.0] | 19.5 [14.8; 26.6] | 6.7 [5.4; 8.8] | 27.7 [20.9; 36.3] | 9.7 [2.2; 17.6] | 46.7 [34.5; 62.0] |
| Cleveland | USA | 1985-2006 | 404057 | 79.2 [59.1; 106.7] | 28.2 [18.8; 41.4] | 13.6 [8.7; 20.1] | 41.9 [32.5; 53.6] | 11.6 [2.9; 20.3] | 69.9 [61.5; 78.2] |
| Cincinnati | USA | 1985-2006 | 171958 | 89.1 [63.1; 114.3] | 26.9 [19.4; 37.3] | 15.4 [10.9; 21.4] | 46.1 [36.8; 56.6] | 13.6 [4.9; 21.2] | 70.6 [62.4; 78.6] |
| Canton | USA | 1985-2006 | 77288 | 93.3 [68.2; 118.6] | 22.9 [16.6; 31.7] | 15.2 [10.7; 21.3] | 27.0 [17.6; 38.0] | 10.9 [1.8; 19] | 73.1 [64.2; 81.4] |
| Columbia | USA | 1985-2006 | 75994 | 82.0 [59.0; 109.4] | 24.2 [15.0; 35.1] | 13.1 [9.4; 17.7] | 11.7 [7.4; 17.2] | 18.6 [11.2; 24.8] | 67.2 [56.7; 77.0] |
| Corpus Christi | USA | 1985-2006 | 45983 | 66.4 [48.0; 88.9] | 26.0 [19.8; 34.0] | 9.1 [6.8; 12.1] | NA | 23.4 [17.9; 27.6] | 76.2 [69.3; 82.6] |
| Davis | USA | 1985-2006 | 17954 | 98.0 [76.9; 117.6] | 22.0 [14.0; 32.0] | 6.8 [4.7; 10.5] | 31.9 [22.2; 47.6] | 10.2 [2.2; 19.7] | 49.1 [33.1; 67.2] |
| Dallas | USA | 1985-2006 | 260718 | 74.2 [52.4; 102.7] | 26.1 [19.4; 34.6] | 11.3 [8.3; 15.8] | 26.9 [19.3; 36.8] | 20.5 [12.7; 27.1] | 61.1 [51.4; 72.1] |
| Denver | USA | 1985-2006 | 182600 | 78.0 [56.3; 103.0] | 25.1 [17.1; 35.2] | 9.1 [7.0; 11.8] | 41.8 [28.3; 58.5] | 10.6 [3.1; 18.8] | 46.5 [35.1; 62.2] |
| Dodge | USA | 1985-2006 | 11259 | 82.6 [64.7; 102.9] | 14.4 [10.3; 20.4] | 9.0 [5.7; 14.7] | NA | 9.1 [0.3; 18.3] | 71.6 [62.4; 80.5] |
| Dover | USA | 1985-2006 | 19772 | 93.3 [71.1; 120.8] | 25.0 [19.0; 29.0] | 10.8 [7.2; 16.5] | NA | 13.6 [5.6; 21.4] | 69.8 [59.4; 80.3] |
| Durham | USA | 1993-2006 | 21566 | 101.9 [77.7; 123.8] | 21.0 [15.0; 28.0] | 13.1 [9.2; 17.9] | 17.8 [13.0; 22.5] | 16.3 [8.7; 23.1] | 69.3 [58.5; 78.9] |

| City | Country | Period | N deaths | Ozone (µg/m$^3$) Median [IQR] | $PM_{10}$ (µg/m$^3$) Median [IQR] | $PM_{2.5}$ (µg/m$^3$) Median [IQR] | $NO_2$ (µg/m$^3$) Median [IQR] | Mean temperature (˚C) Median [IQR] | Relative humidity (%) Median [IQR] |
|---|---|---|---|---|---|---|---|---|---|
| DesMoines | USA | 1985-2006 | 54488 | 45.8 [22.8; 71.3] | 25.9 [18.0; 37.6] | 8.7 [5.8; 13.5] | 20.5 [15.6; 27.5] | 11.3 [1.4; 20.6] | 69.3 [59.5; 78.3] |
| Detroit | USA | 1985-2006 | 729077 | 73.1 [51.5; 98.3] | 29.0 [19.1; 43.5] | 13.4 [8.7; 20.2] | 35.2 [25.4; 46.6] | 10.9 [2.1; 19.8] | 66.7 [58.3; 75.5] |
| Davenport | USA | 1985-2006 | 55256 | 79.1 [57.7; 102.2] | 26.3 [16.3; 39.5] | 10.6 [6.9; 16.1] | 10.3 [7.0; 14.7] | 11.2 [1.5; 20] | 71.3 [61.8; 80.1] |
| Daytona Beach | USA | 1992-2006 | 78470 | 74.8 [59.5; 93.1] | 18.9 [14.8; 23.8] | 8.7 [6.1; 12.1] | NA | 22.7 [17.9; 25.9] | 76.7 [69.8; 82.6] |
| Dayton | USA | 1985-2006 | 108776 | 90.9 [64.4; 115.6] | 24.0 [17.3; 33.9] | 14.5 [10.0; 20.4] | NA | 12.6 [3.4; 20.6] | 71.0 [62.1; 80.0] |
| El centro | USA | 1985-2006 | 16009 | 88.2 [69.8; 110.0] | 49.0 [36.0; 66.1] | 10.0 [7.1; 13.4] | 21.5 [13.2; 35.8] | 23.2 [16.1; 30.9] | 36.1 [28.0; 45.0] |
| Elkhart | USA | 1992-2006 | 18913 | 92.4 [74.4; 112.7] | NA | 12.7 [8.2; 19.0] | NA | 12 [2.6; 21] | 70.3 [60.7; 80.5] |
| El Paso | USA | 1985-2006 | 73269 | 78.9 [61.6; 99.0] | 30.6 [20.3; 45.8] | 9.0 [6.5; 12.5] | 37.6 [27.3; 50.2] | 19.3 [11.2; 25.7] | 36.7 [25.7; 50.0] |
| Elizabeth | USA | 1985-1997 | 59658 | 62.5 [39.4; 91.8] | 28.5 [20.5; 38.2] | NA | 57.5 [44.9; 72.1] | 13.3 [4.9; 21.8] | 61.7 [51.2; 73.8] |
| Erie | USA | 1985-2006 | 54723 | 80.1 [59.8; 106.6] | 16.7 [11.0; 25.4] | 10.8 [7.2; 16.4] | 24.7 [17.9; 33.0] | 10.6 [2.1; 18.9] | 71.0 [62.8; 79.3] |
| Essex | USA | 1985-2006 | 131712 | 70.8 [55.6; 89.8] | 15.0 [11.0; 24.0] | 8.0 [5.2; 12.9] | 17.4 [10.8; 27.3] | 10.3 [3; 18.3] | 67.7 [55.1; 80.1] |
| Eugene | USA | 1985-2006 | 52763 | 71.5 [55.9; 88.9] | 20.7 [12.9; 33.6] | 6.7 [3.7; 11.5] | NA | 11.2 [7; 16.3] | 77.9 [66.5; 87.1] |
| Evansville | USA | 1985-2006 | 37519 | 102.4 [81.1; 124.1] | 26.4 [20.0; 35.7] | 13.4 [9.4; 19.3] | 20.6 [15.2; 27.7] | 14.7 [5.7; 22.6] | 70.4 [62.0; 78.7] |
| Fargo | USA | 1993-2006 | 9567 | 68.6 [53.7; 86.0] | 14.1 [9.3; 22.5] | 6.5 [4.5; 10.0] | 10.8 [6.7; 16.4] | 6.9 [-3.8; 17.5] | 71.6 [62.1; 79.8] |
| Flint | USA | 1985-2006 | 75484 | 83.9 [65.0; 106.3] | 19.0 [13.0; 28.0] | 10.3 [6.0; 15.7] | NA | 9.6 [0.8; 18.3] | 72.7 [64.4; 80.4] |
| Fresno | USA | 1985-2006 | 104033 | 100.2 [63.7; 137.3] | 39.3 [26.5; 57.3] | 13.4 [8.8; 24.0] | 32.4 [23.9; 42.7] | 17.4 [11.3; 24.7] | 53.9 [40.3; 73.8] |
| Fort Lauderdale | USA | 1985-2006 | 308032 | 63.4 [47.9; 80.5] | 16.8 [13.4; 21.3] | 7.5 [5.6; 10.2] | 15.9 [9.7; 22.4] | 25.7 [22.9; 27.9] | 72.4 [66.0; 78.2] |
| Fort Myers | USA | 1985-2006 | 88850 | 72.5 [57.6; 89.8] | 18.2 [14.4; 22.4] | 8.1 [5.7; 10.7] | NA | 24.4 [20.8; 26.9] | 74.6 [67.9; 80.5] |
| Fort Pierce | USA | 1995-2006 | 41824 | 72.0 [55.1; 88.9] | 17.2 [13.9; 22.1] | 8.0 [5.5; 11.1] | 17.3 [12.7; 23.5] | 23.9 [20.2; 26.3] | 76.9 [70.5; 82.0] |
| Fort Worth | USA | 1985-2006 | 172892 | 79.8 [59.5; 109.4] | 21.1 [16.0; 28.6] | 10.8 [7.8; 14.8] | 25.2 [16.1; 36.4] | 19.9 [11.9; 26.5] | 61.9 [51.7; 72.9] |
| Fort Wayne | USA | 1985-2006 | 50899 | 97.7 [78.2; 118.6] | 19.4 [13.8; 29.0] | 12.2 [8.2; 17.9] | 17.4 [10.8; 25.4] | 11.2 [1.9; 19.6] | 73.5 [64.7; 81.8] |
| Fayetville | USA | 1985-2006 | 34790 | 101.9 [78.4; 125.0] | 23.0 [16.5; 31.0] | 12.9 [9.2; 18.1] | NA | 18.1 [10.4; 24.4] | 65.8 [55.0; 76.2] |
| Gary | USA | 1985-2006 | 90669 | 89.7 [69.6; 115.2] | 23.8 [15.8; 35.7] | 13.9 [9.6; 19.2] | 36.9 [27.7; 47.3] | 12.1 [3.1; 21.1] | 68.0 [59.3; 77.4] |

| City | Country | Period | N deaths | Ozone (μg/m$^3$) Median [IQR] | $PM_{10}$ (μg/m$^3$) Median [IQR] | $PM_{2.5}$ (μg/m$^3$) Median [IQR] | $NO_2$ (μg/m$^3$) Median [IQR] | Mean temperature (˚C) Median [IQR] | Relative humidity (%) Median [IQR] |
|---|---|---|---|---|---|---|---|---|---|
| Greensburg | USA | 1985-2006 | 86732 | 82.2 [60.0; 105.8] | 23.4 [17.2; 32.5] | 13.5 [9.2; 19.6] | 28.4 [21.1; 36.3] | 13.3 [4.2; 21.3] | 59.1 [49.8; 69.1] |
| Grand heaven | USA | 1989-2006 | 22493 | 90.7 [72.3; 114.7] | 14.1 [10.3; 20.1] | 11.2 [6.4; 17.5] | 17.8 [14.2; 22.2] | 9.2 [1.2; 17.9] | 73.4 [65.2; 81.1] |
| Grand Junctio | USA | 1986-1992 | 5209 | 89.4 [74.5; 101.9] | 25.1 [18.9; 34.7] | NA | NA | 12.6 [2.2; 21.3] | 44.1 [27.6; 64.0] |
| Grand Rapids | USA | 1985-2006 | 78804 | 84.4 [65.0; 109.8] | 20.0 [14.0; 28.9] | 11.4 [7.0; 18.0] | 27.8 [20.2; 37.0] | 9.4 [0.7; 18.5] | 72.7 [64.1; 81.0] |
| Greensboro | USA | 1985-2006 | 65906 | 106.8 [82.8; 128.1] | 22.5 [15.5; 31.0] | 13.2 [9.1; 18.3] | NA | 15.6 [7.9; 22.3] | 66.9 [55.1; 77.8] |
| Gasinesville | USA | 1985-2006 | 27589 | 78.2 [59.7; 100.2] | 17.1 [13.7; 21.7] | 9.0 [6.6; 12.2] | NA | 21.4 [16.2; 25.2] | 76.2 [68.0; 83.1] |
| Gettysburg | USA | 2002-2006 | 4083 | 93.8 [73.3; 113.4] | NA | 11.0 [7.0; 16.9] | 5.7 [3.7; 8.4] | 12.1 [3.8; 20.1] | 70.5 [59.4; 80.3] |
| Holland | USA | 1990-2006 | 7264 | 94.3 [76.0; 121.0] | 14.0 [8.0; 20.5] | 9.9 [5.7; 16.3] | 13.9 [9.2; 20.1] | 9.4 [1.6; 18.3] | 73.6 [65.1; 81.2] |
| Harrisburg | USA | 1985-2006 | 49992 | 79.4 [52.6; 108.9] | 19.9 [13.1; 28.6] | 13.4 [8.4; 20.3] | 33.4 [24.0; 44.4] | 12.9 [4.2; 21.1] | 64.0 [53.0; 75.6] |
| Hartford | USA | 1985-2006 | 159050 | 76.2 [55.1; 98.2] | 16.7 [11.2; 25.9] | 9.3 [6.2; 14.9] | 32.4 [22.6; 44.6] | 11.7 [3.4; 19.8] | 65.7 [54.2; 77.2] |
| Houston | USA | 1985-2006 | 366340 | 66.4 [47.8; 95.8] | 27.0 [19.1; 36.9] | 12.1 [9.0; 15.8] | 32.5 [23.4; 43.8] | 22.2 [16.3; 26.9] | 75.3 [66.6; 82.3] |
| Indianapolis | USA | 1985-2006 | 149459 | 98.9 [77.2; 121.5] | 27.0 [19.1; 38.0] | 14.6 [10.0; 20.3] | 33.7 [25.6; 42.9] | 12.9 [3.5; 21.1] | 71.2 [62.1; 80.0] |
| Iowa city | USA | 1985-1991 | 2871 | 68.6 [50.0; 90.6] | NA | NA | NA | 10.4 [0.5; 19.9] | 71.4 [61.6; 80.4] |
| Jacksonville | USA | 1985-2006 | 124017 | 77.3 [59.6; 99.0] | 26.0 [20.0; 33.0] | 9.6 [6.8; 13.0] | 26.4 [19.9; 34.3] | 22.4 [16.9; 26.5] | 69.3 [61.2; 76.6] |
| Jersey city | USA | 1985-2006 | 103084 | 60.0 [36.3; 90.9] | 28.5 [20.0; 39.6] | 12.8 [8.3; 19.6] | 49.7 [37.4; 64.0] | 10.3 [3.6; 17.7] | 73.8 [62.6; 84.3] |
| Kalamazoo | USA | 1991-2006 | 26670 | 84.8 [61.4; 106.3] | 16.0 [10.0; 24.5] | 11.9 [7.2; 18.7] | 25.0 [18.6; 32.6] | 10.6 [1.6; 19.6] | 70.3 [60.7; 78.9] |
| Kenosha | USA | 1985-2006 | 23194 | 89.5 [70.8; 114.7] | NA | 9.8 [6.0; 15.5] | 25.6 [16.0; 38.3] | 10 [1.6; 18.9] | 72.3 [63.9; 80.6] |
| Kansas | USA | 1985-2006 | 218933 | 63.0 [39.2; 91.9] | 29.2 [21.0; 40.1] | 10.8 [7.4; 15.2] | 24.1 [17.0; 32.6] | 14.8 [5.2; 23] | 64.6 [55.4; 74.3] |
| Knoxville | USA | 1985-2006 | 80418 | 91.3 [71.0; 115.0] | 27.0 [19.0; 36.5] | 14.5 [10.1; 20.0] | 6.0 [2.6; 15.2] | 15.9 [7.9; 22.8] | 70.9 [61.6; 79.5] |
| La Fayette LA | USA | 1985-2006 | 24508 | 76.0 [56.4; 101.7] | 18.0 [14.0; 24.0] | 10.1 [7.3; 13.9] | NA | 21.4 [14.9; 26] | 76.2 [67.5; 83.3] |
| Lake Charles | USA | 1985-2006 | 31479 | 74.1 [55.9; 97.0] | 19.0 [14.0; 26.0] | 9.9 [7.2; 13.7] | 10.1 [7.0; 14.1] | 22.5 [16.1; 27.2] | 71.6 [61.4; 80.7] |
| Lakeland | USA | 1992-2006 | 70007 | 75.1 [58.2; 94.7] | 19.7 [15.7; 24.6] | 9.3 [6.6; 12.6] | NA | 24.7 [20.4; 27.5] | 66.7 [58.8; 74.0] |
| Lancaster | USA | 1985-2006 | 80724 | 83.3 [53.4; 114.0] | 19.8 [13.5; 28.6] | 14.3 [9.5; 21.5] | 29.0 [20.9; 38.2] | 12.6 [4.1; 20.9] | 65.6 [55.1; 76.9] |

| City | Country | Period | N deaths | Ozone (μg/m$^3$) Median [IQR] | $PM_{10}$ (μg/m$^3$) Median [IQR] | $PM_{2.5}$ (μg/m$^3$) Median [IQR] | $NO_2$ (μg/m$^3$) Median [IQR] | Mean temperature (˚C) Median [IQR] | Relative humidity (%) Median [IQR] |
|---|---|---|---|---|---|---|---|---|---|
| Lousville | USA | 1985-2006 | 139347 | 80.4 [52.7; 108.4] | 23.5 [17.0; 33.1] | 14.1 [9.9; 19.5] | 34.3 [26.3; 44.7] | 15.3 [6.4; 22.9] | 67.1 [57.9; 76.6] |
| LaPorte | USA | 1990-2006 | 15900 | 95.1 [76.3; 118.3] | 18.5 [12.8; 24.0] | 11.8 [7.3; 17.1] | NA | 10.7 [1.7; 19.3] | 72.7 [63.5; 81.3] |
| Los Angeles | USA | 1985-2006 | 1239036 | 80.3 [49.5; 115.5] | 34.3 [25.4; 43.5] | 15.8 [11.1; 22.2] | 63.6 [48.1; 81.9] | 17.9 [15.2; 20.2] | 71.0 [60.9; 77.8] |
| Las Vegas | USA | 1985-2006 | 182220 | 86.0 [63.1; 108.4] | 31.9 [23.2; 42.0] | 6.5 [4.4; 9.1] | 30.6 [18.7; 54.1] | 20.3 [11.9; 29.3] | 25.7 [18.0; 37.2] |
| Little Rock | USA | 1985-2006 | 63901 | 76.7 [56.6; 101.8] | 25.7 [18.8; 34.8] | 12.8 [9.2; 17.8] | 19.0 [13.4; 26.6] | 17.9 [9.7; 24.7] | 68.9 [59.4; 78.1] |
| Macon | USA | 1997-2006 | 15318 | 98.7 [76.5; 122.7] | 26.0 [17.0; 36.0] | 14.1 [10.3; 19.0] | NA | 18.8 [11.9; 24.8] | 69.4 [59.2; 78.0] |
| Mc Allen | USA | 1996-2006 | 29196 | 64.6 [49.6; 81.6] | 25.0 [18.9; 34.3] | 9.5 [7.2; 12.5] | NA | 25 [19.9; 28.7] | 68.4 [61.4; 76.0] |
| Middlesex | USA | 1985-2006 | 110324 | 68.6 [45.6; 101.2] | 22.2 [16.5; 30.7] | 9.7 [6.4; 15.4] | 31.4 [22.3; 43.9] | 12.9 [4.6; 21.1] | 63.2 [51.7; 75.5] |
| Middletown | USA | 1985-2006 | 49618 | 89.7 [63.0; 115.4] | 27.5 [19.4; 40.4] | 14.5 [10.3; 20.4] | NA | 13.4 [4.6; 21.1] | 69.8 [61.4; 78.0] |
| Medford | USA | 1985-2006 | 33409 | 83.5 [67.6; 102.5] | 28.5 [18.8; 43.7] | 7.4 [4.5; 12.3] | NA | 12 [6.4; 18.9] | 62.6 [47.5; 79.9] |
| Madison IL | USA | 1985-2006 | 48230 | 69.0 [46.1; 98.0] | 33.8 [21.4; 51.7] | 15.3 [10.3; 20.5] | NA | 15.6 [6.1; 23.6] | 66.5 [56.1; 76.5] |
| Modesto | USA | 1985-2006 | 63591 | 80.4 [52.2; 111.5] | 31.9 [22.6; 45.3] | 11.0 [8.0; 20.0] | 32.9 [25.0; 42.5] | 17.9 [11.7; 24.2] | 54.3 [40.9; 71.3] |
| Madison WI | USA | 1985-2006 | 48763 | 83.1 [62.5; 102.4] | 21.0 [14.5; 29.0] | 10.2 [6.6; 16.1] | NA | 9.3 [0.1; 18.4] | 70.5 [61.7; 78.8] |
| Miami | USA | 1985-2006 | 372130 | 65.1 [49.8; 82.7] | 24.6 [19.9; 30.4] | 8.4 [6.3; 11.3] | 18.6 [12.8; 27.5] | 25.4 [22.8; 27.7] | 72.7 [67.2; 78.0] |
| Melbourn | USA | 1988-2006 | 80122 | 75.0 [59.3; 91.9] | 16.5 [13.0; 20.9] | 7.5 [5.1; 10.4] | NA | 23.9 [20.1; 26.6] | 76.0 [70.0; 81.3] |
| Milwauke | USA | 1985-2006 | 232056 | 76.2 [56.5; 98.4] | 25.1 [17.3; 35.3] | 11.2 [7.1; 17.3] | 36.3 [26.9; 47.4] | 9.2 [1.1; 18.4] | 70.4 [61.7; 79.2] |
| Memphis | USA | 1985-2006 | 152003 | 101.4 [78.9; 124.2] | 24.2 [17.5; 32.7] | 12.1 [8.6; 16.7] | 44.1 [31.9; 57.6] | 18.4 [10.1; 25.2] | 65.5 [55.9; 75.8] |
| Monmouth | USA | 1985-2006 | 235036 | 89.1 [64.2; 117.4] | NA | 9.0 [6.0; 13.9] | NA | 13.6 [5.7; 21.6] | 60.7 [49.6; 73.1] |
| Montgomery | USA | 1986-2006 | 38531 | 87.5 [66.2; 110.0] | 21.0 [15.3; 28.0] | 13.0 [9.9; 18.5] | 19.3 [14.3; 25.2] | 20.7 [13.7; 26.6] | 66.0 [56.0; 76.1] |
| Mobile | USA | 1985-2006 | 72746 | 79.7 [59.7; 102.3] | 25.9 [19.1; 34.7] | 11.4 [8.5; 16.0] | NA | 21.4 [15.1; 26.4] | 72.6 [61.3; 81.8] |
| Monroe | USA | 1985-2006 | 26416 | 78.9 [59.3; 103.4] | 25.0 [17.0; 37.0] | 10.7 [7.6; 15.4] | 16.0 [11.6; 21.9] | 19.7 [12.2; 25.6] | 72.8 [64.0; 80.8] |
| Mercer | USA | 1985-2006 | 27870 | 81.8 [57.1; 111.5] | 30.0 [22.0; 42.0] | 11.9 [8.1; 17.3] | NA | 10.3 [1.3; 18.2] | 71.3 [61.9; 79.9] |
| Marlboro | USA | 1985-2006 | 81024 | 87.0 [56.8; 121.5] | 20.4 [14.0; 29.5] | 11.8 [8.1; 17.3] | 18.1 [13.2; 25.6] | 13.9 [5.8; 21.7] | 66.3 [55.6; 77.5] |

| City | Country | Period | N deaths | Ozone (μg/m$^3$) Median [IQR] | $PM_{10}$ (μg/m$^3$) Median [IQR] | $PM_{2.5}$ (μg/m$^3$) Median [IQR] | $NO_2$ (μg/m$^3$) Median [IQR] | Mean temperature (˚C) Median [IQR] | Relative humidity (%) Median [IQR] |
|---|---|---|---|---|---|---|---|---|---|
| Muskegon | USA | 1985-2006 | 30902 | 89.9 [71.1; 116.4] | 25.8 [17.6; 39.0] | 9.8 [5.4; 16.1] | NA | 9.3 [1.1; 18] | 73.1 [64.7; 80.9] |
| Muncie | USA | 2001-2006 | 6539 | 100.7 [82.6; 119.3] | NA | 12.3 [8.2; 17.0] | NA | 12.3 [2.9; 20.1] | 72.8 [63.8; 82.0] |
| Nashua | USA | 1985-2006 | 51115 | 77.2 [58.8; 97.0] | 16.2 [11.0; 23.5] | 7.5 [4.9; 12.2] | 24.9 [16.3; 35.9] | 11.1 [2.6; 19.9] | 60.1 [48.9; 73.7] |
| Nassau | USA | 1985-2006 | 460192 | 64.9 [45.1; 90.5] | 17.3 [12.3; 24.1] | 9.8 [6.2; 15.2] | 44.1 [31.6; 58.7] | 12.2 [4.7; 20.3] | 66.6 [55.0; 79.6] |
| Niles | USA | 1991-2006 | 22196 | 99.0 [80.6; 122.7] | NA | 10.2 [6.0; 16.1] | 12.4 [10.2; 14.9] | 10.5 [1.6; 19.2] | 72.1 [63.5; 80.3] |
| Nashville | USA | 1985-2006 | 97358 | 65.5 [41.0; 92.7] | 29.2 [21.6; 39.7] | 12.5 [9.0; 17.4] | 25.9 [16.6; 36.3] | 16.7 [8.3; 23.7] | 66.6 [57.3; 76.1] |
| Newburgh | USA | 1995-2006 | 27492 | 90.6 [69.8; 110.7] | NA | 8.9 [5.9; 14.4] | NA | 10.9 [2.5; 19] | 64.6 [54.9; 76.0] |
| Newhaven | USA | 1985-2006 | 157415 | 79.9 [61.6; 102.3] | 23.4 [15.3; 34.8] | 11.4 [7.6; 17.5] | 45.8 [34.0; 58.9] | 12.1 [4.6; 20.2] | 72.6 [59.6; 83.1] |
| Newlond | USA | 1985-2006 | 40419 | 92.6 [75.2; 115.2] | 15.2 [11.0; 21.6] | 8.9 [6.0; 14.4] | NA | 11.5 [4.4; 19.1] | 72.2 [59.0; 84.5] |
| New Orleans | USA | 1985-1989 | 41816 | 73.0 [50.4; 103.2] | 30.9 [22.0; 41.8] | NA | 34.6 [26.9; 44.3] | 21.9 [15.8; 26.6] | 75.4 [68.0; 82.7] |
| Newark | USA | 1985-2006 | 220980 | 66.4 [45.3; 95.1] | 31.4 [22.0; 43.5] | 10.3 [6.7; 16.4] | 44.3 [32.8; 57.9] | 13.3 [5.2; 21.7] | 61.5 [51.1; 73.7] |
| New York | USA | 1985-2006 | 1367085 | 50.5 [30.9; 77.8] | 22.3 [16.3; 31.6] | 12.4 [8.2; 18.5] | 65.8 [53.3; 81.1] | 10.2 [3.5; 17.7] | 73.8 [62.7; 84.3] |
| Ocala | USA | 1998-2006 | 29695 | 77.7 [61.2; 97.5] | NA | 9.4 [6.8; 12.7] | NA | 22 [16.8; 25.4] | 70.9 [62.2; 78.1] |
| Oklahoma | USA | 1985-2006 | 118753 | 80.1 [58.6; 105.2] | 21.6 [15.7; 30.0] | 8.3 [5.9; 12.1] | 20.9 [14.9; 29.9] | 16.8 [8.1; 24.3] | 62.8 [52.6; 74.0] |
| Oakland | USA | 1985-2006 | 325028 | 60.7 [46.0; 74.1] | 19.9 [14.2; 28.2] | 9.1 [5.8; 15.3] | 30.9 [22.0; 41.1] | 14.4 [11.9; 16.4] | 77.8 [70.3; 83.4] |
| Omaha | USA | 1985-2006 | 71558 | 64.7 [47.0; 84.4] | 33.3 [24.2; 45.9] | 8.7 [6.0; 12.9] | NA | 11.6 [1.7; 20.7] | 68.7 [59.3; 77.5] |
| Orlando | USA | 1985-2006 | 157019 | 76.9 [60.5; 97.1] | 19.0 [14.9; 23.9] | 9.1 [6.7; 12.3] | 19.7 [14.1; 26.9] | 23.4 [19.3; 26.4] | 73.3 [66.0; 79.9] |
| Philadelphia | USA | 1985-2006 | 911888 | 64.3 [40.1; 99.3] | 24.2 [17.7; 34.2] | 12.2 [8.1; 18.1] | 46.1 [35.8; 57.7] | 13.1 [5.1; 21.2] | 66.1 [56.2; 77.0] |
| Phoenix | USA | 1985-2006 | 386802 | 89.1 [63.3; 110.4] | 42.8 [30.9; 57.7] | 9.3 [7.1; 13.3] | 42.9 [30.4; 57.8] | 24.1 [16.1; 31.9] | 28.9 [20.3; 41.2] |
| Palm beach | USA | 1985-2006 | 233887 | 62.0 [46.9; 79.0] | 18.9 [15.1; 24.0] | 7.2 [5.2; 9.8] | 23.6 [17.1; 30.8] | 24.9 [22; 27.2] | 74.5 [68.5; 79.8] |
| Plymouth | USA | 1987-1997 | 37343 | 70.6 [53.2; 93.8] | NA | NA | NA | 10.7 [3.1; 18.4] | 64.8 [53.8; 77.4] |
| Pensacola | USA | 1985-2006 | 50546 | 82.1 [62.9; 105.0] | 19.7 [15.2; 25.5] | 10.8 [7.8; 15.4] | 13.1 [9.0; 18.4] | 21.2 [15.2; 26.2] | 76.3 [65.6; 84.0] |
| Portland OR | USA | 1985-2006 | 210301 | 63.8 [49.5; 80.2] | 20.1 [13.7; 29.1] | 7.4 [5.0; 11.1] | 26.9 [20.4; 35.8] | 11.9 [7.4; 17.3] | 73.0 [62.3; 82.9] |

| City | Country | Period | N deaths | Ozone (µg/m³) Median [IQR] | $PM_{10}$ (µg/m³) Median [IQR] | $PM_{2.5}$ (µg/m³) Median [IQR] | $NO_2$ (µg/m³) Median [IQR] | Mean temperature (˚C) Median [IQR] | Relative humidity (%) Median [IQR] |
|---|---|---|---|---|---|---|---|---|---|
| Port Arthur | USA | 1985-2006 | 50969 | 70.4 [50.0; 97.4] | 20.0 [14.0; 26.0] | 10.0 [7.3; 13.7] | 14.3 [7.7; 21.5] | 21.6 [15.4; 26.5] | 78.7 [70.4; 85.0] |
| Portage | USA | 1985-2006 | 20147 | 86.9 [65.2; 111.5] | 17.5 [12.0; 25.1] | 11.7 [7.5; 16.9] | NA | 12.1 [3.1; 21.1] | 68.0 [59.3; 77.4] |
| Portland ME | USA | 1985-2006 | 46217 | 74.7 [59.5; 93.4] | 18.8 [13.5; 26.4] | 8.9 [6.3; 13.9] | 26.7 [17.9; 36.7] | 8.4 [0.9; 16.6] | 69.4 [58.0; 81.2] |
| Providence | USA | 1985-2006 | 254651 | 83.5 [64.9; 104.4] | 22.5 [16.2; 31.7] | 9.1 [6.4; 13.7] | 30.0 [19.2; 43.4] | 11 [3.6; 19] | 68.0 [55.8; 80.4] |
| Pittsburg | USA | 1985-2006 | 317935 | 63.4 [40.0; 96.8] | 24.4 [15.2; 38.6] | 13.2 [8.2; 20.6] | 44.7 [33.9; 56.4] | 12.3 [3.3; 20] | 68.2 [58.4; 77.8] |
| Richmond | USA | 1985-2006 | 116646 | 93.7 [64.7; 119.4] | 21.0 [15.0; 29.3] | 12.4 [8.6; 17.6] | 38.8 [28.9; 49.5] | 15.3 [7.5; 22.7] | 67.6 [56.8; 78.4] |
| Rochester | USA | 1985-2006 | 127040 | 62.5 [45.3; 84.8] | 19.5 [13.9; 28.0] | 9.2 [5.9; 14.8] | NA | 9.5 [1.2; 18.1] | 71.8 [63.9; 79.4] |
| Rockville | USA | 1985-2006 | 92381 | 89.7 [60.0; 120.5] | 23.0 [16.0; 32.0] | 10.8 [7.5; 16.4] | NA | 14.7 [6.6; 22.8] | 65.1 [54.0; 76.1] |
| Reading | USA | 1985-2006 | 72337 | 77.4 [51.2; 106.8] | 20.1 [14.3; 28.2] | 13.7 [8.7; 20.4] | 38.6 [30.2; 47.8] | 12.7 [4.2; 20.9] | 65.6 [55.1; 76.0] |
| Reno | USA | 1985-2006 | 45340 | 78.7 [57.8; 98.5] | 33.0 [22.0; 49.5] | 6.5 [4.6; 9.0] | 26.2 [14.1; 43.0] | 11.3 [4.5; 19.6] | 41.5 [29.6; 57.5] |
| Raleigh | USA | 1985-2006 | 58561 | 97.3 [71.0; 123.5] | 20.3 [14.8; 27.3] | 13.1 [9.3; 18.0] | 22.3 [15.5; 32.1] | 16.4 [8.8; 23.1] | 68.7 [57.7; 78.6] |
| Riverside | USA | 1985-2006 | 433285 | 101.5 [69.2; 139.7] | 36.3 [25.9; 46.5] | 15.3 [9.6; 23.2] | 43.7 [33.5; 54.5] | 17.1 [13; 22.1] | 56.5 [42.0; 68.6] |
| Sacramento | USA | 1985-2006 | 172136 | 80.6 [55.7; 105.7] | 22.9 [16.4; 31.4] | 9.1 [6.0; 14.4] | 26.2 [18.8; 35.9] | 15.8 [10.8; 20.8] | 64.5 [51.9; 77.8] |
| Scranton | USA | 1985-2006 | 150119 | 77.5 [56.3; 102.2] | 17.9 [11.9; 26.9] | 10.0 [6.0; 16.2] | 28.5 [20.1; 39.0] | 10.7 [2.2; 18.7] | 67.9 [58.4; 77.5] |
| San Diego | USA | 1985-2006 | 369956 | 87.5 [70.5; 105.1] | 31.6 [24.0; 40.5] | 11.4 [8.1; 16.1] | 37.9 [27.9; 51.4] | 17.8 [14.8; 20.6] | 68.4 [59.5; 74.5] |
| San Francisco | USA | 1985-2006 | 248607 | 47.9 [35.2; 61.2] | 22.6 [15.6; 32.5] | 9.1 [5.8; 14.4] | 34.9 [23.6; 50.1] | 14.6 [12.2; 16.6] | 75.4 [68.3; 81.5] |
| Salt Lake | USA | 1985-2006 | 89770 | 94.9 [71.8; 114.5] | 29.8 [19.5; 43.1] | 7.7 [5.6; 11.9] | 47.4 [34.8; 63.6] | 11.2 [3.3; 21] | 52.1 [36.2; 69.4] |
| San Jose | USA | 1985-2006 | 176066 | 61.7 [44.3; 77.4] | 25.1 [17.3; 35.4] | 9.9 [6.4; 16.8] | 42.8 [31.0; 57.4] | 17.2 [13.2; 21.1] | 59.7 [50.9; 68.8] |
| San Antonio | USA | 1985-2006 | 186461 | 78.3 [62.8; 99.7] | 22.0 [16.0; 29.5] | 8.7 [6.4; 12.1] | 16.6 [11.4; 24.3] | 22.5 [15.7; 27.7] | 65.4 [55.0; 75.6] |
| Spokane | USA | 1985-2006 | 68681 | 87.0 [73.3; 100.7] | 24.1 [14.3; 38.3] | 7.4 [4.5; 11.4] | NA | 9.2 [2.9; 16.6] | 65.2 [50.0; 80.5] |
| Springfield MA | USA | 1985-2006 | 94971 | 70.2 [51.0; 93.4] | 23.2 [16.4; 32.8] | 10.0 [6.5; 15.7] | 31.4 [21.9; 44.8] | 10.9 [2.2; 19.2] | 64.3 [54.1; 76.0] |
| Springfied MO | USA | 1985-2006 | 43239 | 78.3 [56.9; 99.0] | 18.0 [13.0; 25.0] | 11.0 [7.5; 14.7] | 19.8 [12.9; 27.6] | 14.6 [5.7; 22] | 69.2 [59.6; 78.5] |
| Spartanburg | USA | 1990-2006 | 37387 | 90.9 [61.2; 118.6] | 21.0 [16.0; 28.0] | 13.1 [9.1; 18.2] | NA | 16.7 [9.3; 22.9] | 67.7 [55.3; 79.0] |

| City | Country | Period | N deaths | Ozone (µg/m³) Median [IQR] | $PM_{10}$ (µg/m³) Median [IQR] | $PM_{2.5}$ (µg/m³) Median [IQR] | $NO_2$ (µg/m³) Median [IQR] | Mean temperature (˚C) Median [IQR] | Relative humidity (%) Median [IQR] |
|---|---|---|---|---|---|---|---|---|---|
| Sarasota | USA | 1985-2006 | 151551 | 75.1 [58.7; 94.6] | 19.9 [15.6; 24.6] | 8.5 [6.0; 11.8] | 8.7 [5.5; 13.3] | 24.1 [20.1; 27.1] | 76.1 [69.3; 81.8] |
| Steubenville | USA | 1985-2006 | 21002 | 71.1 [45.3; 101.9] | 28.6 [19.6; 42.6] | 14.7 [10.4; 21.6] | 33.8 [26.2; 43.3] | 12.6 [3.6; 20.4] | 67.3 [56.5; 77.4] |
| Saint Charles | USA | 1985-2006 | 28225 | 87.5 [59.6; 114.7] | 20.0 [13.0; 28.0] | 12.3 [8.0; 17.4] | 17.0 [10.3; 25.2] | 15 [5.4; 23.2] | 65.1 [55.2; 75.3] |
| Stockton | USA | 1985-2006 | 82225 | 72.9 [49.0; 97.7] | 26.5 [18.6; 37.2] | 11.0 [7.0; 17.0] | 35.7 [26.6; 46.1] | 16.3 [11; 21.8] | 60.7 [47.7; 76.2] |
| South bend | USA | 1985-2006 | 48505 | 93.8 [74.0; 115.8] | 22.0 [14.6; 32.6] | 12.1 [7.4; 17.6] | 24.8 [18.1; 32.4] | 10.5 [1.6; 19.3] | 71.9 [63.1; 80.2] |
| St Louis | USA | 1985-2006 | 312923 | 79.8 [55.9; 106.2] | 25.5 [15.2; 39.4] | 12.7 [8.9; 18.2] | 34.8 [26.9; 44.0] | 15.6 [6.1; 23.6] | 66.5 [56.1; 76.5] |
| Stamford | USA | 1985-2006 | 142216 | 86.3 [66.5; 109.6] | 21.5 [15.6; 30.9] | 10.4 [6.5; 16.7] | 37.2 [26.2; 50.4] | 11.3 [3.5; 19.2] | 66.9 [56.1; 78.6] |
| St. Petersbur | USA | 1985-2006 | 158555 | 73.7 [57.4; 94.1] | 21.7 [17.1; 27.0] | 9.3 [6.9; 12.6] | 19.9 [12.1; 29.9] | 24.7 [20.3; 27.9] | 72.8 [65.6; 79.6] |
| State College | USA | 1996-2006 | 8609 | 92.9 [72.2; 113.4] | NA | 10.9 [6.8; 16.5] | 14.0 [9.6; 21.0] | 11.2 [2.6; 19.1] | 64.8 [54.7; 75.0] |
| Seattle | USA | 1985-2006 | 225451 | 59.0 [42.8; 77.2] | 22.7 [15.9; 32.9] | 7.9 [5.4; 11.6] | 32.3 [23.2; 42.2] | 10.7 [7.7; 14.2] | 82.3 [76.9; 86.8] |
| Tacoma | USA | 1985-2006 | 96086 | 63.5 [50.5; 78.1] | 24.3 [15.4; 38.2] | 8.4 [5.3; 13.7] | NA | 11.3 [7.3; 16.2] | 74.4 [63.6; 84.2] |
| Tampa | USA | 1985-2006 | 158555 | 75.8 [59.3; 97.1] | 24.6 [19.0; 30.5] | 10.6 [7.9; 14.3] | 15.9 [10.6; 23.3] | 23.7 [19.4; 26.9] | 74.4 [67.7; 80.2] |
| Tucson | USA | 1985-2006 | 131053 | 85.3 [67.8; 102.7] | 25.3 [18.4; 34.7] | 6.0 [4.7; 7.6] | 33.9 [25.0; 44.6] | 21.3 [14; 27.9] | 31.6 [20.7; 46.5] |
| Tallahassee | USA | 1985-2006 | 25858 | 74.4 [57.2; 97.1] | 15.6 [12.1; 20.2] | 11.6 [8.2; 16.4] | NA | 20.9 [14.7; 25.6] | 72.5 [62.6; 81.0] |
| Toledo | USA | 1985-2006 | 92004 | 81.7 [59.3; 104.9] | 20.3 [14.6; 29.4] | 12.8 [8.6; 18.7] | NA | 10.8 [1.7; 19.6] | 71.8 [63.2; 80.2] |
| Trenton | USA | 1985-2006 | 58430 | 72.0 [49.0; 105.0] | 24.0 [16.0; 33.0] | 10.1 [6.4; 15.9] | 27.6 [19.5; 37.9] | 12.7 [4.6; 20.9] | 62.3 [52.3; 73.0] |
| Terra Haute | USA | 1985-2006 | 23821 | 94.3 [74.0; 115.6] | 24.9 [17.7; 36.1] | 12.1 [8.3; 17.5] | NA | 13.1 [3.8; 21.2] | 72.9 [64.3; 80.9] |
| Tulsa | USA | 1985-2006 | 95475 | 84.4 [62.2; 109.2] | 23.6 [16.6; 32.2] | 9.7 [6.7; 13.6] | 21.2 [14.2; 29.5] | 17 [8.3; 24.5] | 64.3 [54.2; 74.8] |
| Visalia | USA | 1985-2006 | 50358 | 101.2 [67.0; 144.6] | 46.8 [32.1; 63.9] | 16.0 [10.3; 24.0] | 35.3 [27.1; 45.2] | 17.4 [11.1; 24] | 58.0 [47.7; 77.2] |
| Vancouver | USA | 1985-2006 | 37914 | 63.7 [49.0; 79.0] | 15.5 [11.0; 22.1] | 6.9 [4.7; 11.4] | 23.4 [17.2; 30.3] | 11.8 [7.3; 17.2] | 72.8 [61.8; 82.4] |
| Ventura | USA | 1985-2006 | 87603 | 95.3 [74.2; 117.6] | 28.7 [18.8; 37.0] | 11.4 [6.9; 15.4] | 23.0 [17.5; 29.8] | 16.4 [14; 18.7] | 75.5 [66.5; 81.8] |
| Wichita | USA | 1985-2006 | 68542 | 69.2 [50.0; 93.1] | 21.9 [15.2; 30.4] | 9.8 [6.7; 13.5] | 17.6 [12.4; 24.6] | 14.5 [5.4; 23.1] | 65.3 [55.2; 75.4] |
| Weber | USA | 1985-2006 | 24455 | 108.0 [94.2; 122.5] | 25.0 [16.0; 35.0] | 7.8 [5.4; 11.6] | 45.0 [32.7; 58.5] | 11.6 [3.2; 21.7] | 47.9 [32.3; 66.4] |

| City | Country | Period | N deaths | Ozone (μg/m³) Median [IQR] | $PM_{10}$ (μg/m³) Median [IQR] | $PM_{2.5}$ (μg/m³) Median [IQR] | $NO_2$ (μg/m³) Median [IQR] | Mean temperature (˚C) Median [IQR] | Relative humidity (%) Median [IQR] |
|---|---|---|---|---|---|---|---|---|---|
| Wilmington | USA | 1985-2006 | 76254 | 67.7 [44.1; 98.7] | 22.6 [16.0; 31.9] | 13.0 [8.8; 19.2] | 37.6 [24.3; 52.0] | 13.1 [5.1; 21.3] | 67.5 [57.3; 78.2] |
| Winston | USA | 1985-2006 | 52543 | 101.0 [79.1; 123.0] | 22.0 [15.7; 29.7] | 13.4 [9.0; 18.8] | 26.8 [19.4; 36.3] | 16.4 [8.7; 23] | 63.0 [50.4; 75.5] |
| Worcester | USA | 1985-2006 | 135785 | 79.4 [61.5; 101.9] | 19.1 [13.6; 28.1] | 9.4 [6.2; 14.5] | 38.3 [26.9; 51.6] | 9.1 [0.8; 17.3] | 67.2 [56.1; 79.4] |
| WDC | USA | 1985-2006 | 141028 | 56.8 [34.7; 91.5] | 25.1 [17.6; 34.9] | 13.3 [8.9; 18.9] | 44.4 [34.4; 56.0] | 14.7 [6.7; 22.8] | 65.1 [54.0; 76.1] |
| Washington | USA | 1985-2006 | 49002 | 82.6 [54.8; 110.6] | 18.0 [12.0; 27.0] | 12.6 [9.0; 18.2] | 28.8 [20.6; 38.1] | 11.8 [3.2; 19.2] | 66.5 [56.2; 75.6] |
| Youngstown | USA | 1985-2006 | 86656 | 86.8 [61.2; 114.5] | 23.0 [16.0; 33.2] | 13.6 [9.5; 19.5] | 31.6 [23.1; 43.1] | 10.6 [1.7; 18.4] | 72.7 [64.1; 81.2] |
| York | USA | 1985-2006 | 62767 | 79.9 [51.9; 111.7] | 22.2 [15.4; 31.8] | 14.7 [9.6; 21.9] | 37.7 [27.5; 48.2] | 12.2 [3.9; 20.5] | 67.2 [56.5; 77.7] |

Ozone: daily maximum 8-hour mean, μg/m³. Mean temperature, ˚C. $PM_{10}$: inhalable particulate matter with an aerodynamic diameter of 10 μm or less, μg/m³. $PM_{2.5}$: inhalable particulate matter with an aerodynamic diameter of 2.5 μm or less, μg/m³. $NO_2$: nitrogen dioxide, μg/m³. IQR: interquartile range. Relative humidity, in %.

**eTable 3.** Overall and country-specific excess mortality fractions (%, 95% confidence interval) associated to ozone for the total (above 70 μg/m$^3$) and above specific thresholds consistent with current air quality standards (AQS).

| | **Total* (Above 70 μg/m$^3$)** | **Above WHO AQS (100 μg/m$^3$)** | **Above EU AQS (120 μg/m$^3$)** | **Above NAAQS in the US (140 μg/m$^3$)** | **Above CAAQS in China (160 μg/m$^3$)** |
|---|---|---|---|---|---|
| Australia** | 0.00 [0.00; 0.00] | 0.00 [0.00; 0.00] | 0.00 [0.00; 0.00] | 0.00 [0.00; 0.00] | 0.00 [0.00; 0.00] |
| Canada | 0.34 [0.29; 0.41] | 0.24 [0.18; 0.30] | 0.16 [0.10; 0.23] | 0.11 [0.04; 0.18] | 0.07 [-0.01; 0.14] |
| China | 0.22 [0.04; 0.39] | 0.19 [0.00; 0.36] | 0.14 [-0.05; 0.32] | 0.10 [-0.11; 0.29] | 0.06 [-0.15; 0.26] |
| Czech Republic | 0.27 [0.02; 0.48] | 0.20 [-0.06; 0.44] | 0.12 [-0.16; 0.39] | 0.06 [-0.24; 0.34] | 0.03 [-0.30; 0.34] |
| Estonia | 0.02 [0.01; 0.03] | 0.00 [-0.01; 0.01] | 0.00 [-0.01; 0.01] | 0.00 [-0.01; 0.01] | 0.00 [-0.01; 0.01] |
| France | 0.20 [0.15; 0.25] | 0.14 [0.09; 0.19] | 0.08 [0.02; 0.13] | 0.04 [-0.02; 0.10] | 0.02 [-0.05; 0.08] |
| Germany | 0.12 [0.09; 0.15] | 0.08 [0.05; 0.11] | 0.05 [0.01; 0.08] | 0.02 [-0.02; 0.06] | 0.01 [-0.03; 0.05] |
| Greece | 0.16 [-0.07; 0.41] | 0.11 [-0.13; 0.37] | 0.04 [-0.27; 0.33] | 0.01 [-0.31; 0.34] | 0.00 [-0.33; 0.35] |
| Italy | 0.27 [0.13; 0.42] | 0.19 [0.04; 0.35] | 0.09 [-0.08; 0.27] | 0.03 [-0.15; 0.23] | 0.01 [-0.20; 0.21] |
| Japan | 0.31 [0.27; 0.35] | 0.21 [0.17; 0.26] | 0.12 [0.08; 0.16] | 0.05 [0.00; 0.10] | 0.02 [-0.03; 0.07] |
| Mexico | 0.55 [0.16; 0.94] | 0.52 [0.14; 0.92] | 0.48 [0.09; 0.87] | 0.42 [0.03; 0.81] | 0.35 [-0.07; 0.75] |
| Portugal | 0.08 [0.00; 0.16] | 0.04 [-0.05; 0.13] | 0.02 [-0.09; 0.11] | 0.01 [-0.10; 0.11] | 0.00 [-0.11; 0.12] |
| South Africa | 0.28 [0.20; 0.35] | 0.18 [0.11; 0.26] | 0.10 [0.01; 0.19] | 0.04 [-0.05; 0.15] | 0.02 [-0.09; 0.13] |
| South Korea | 0.12 [0.08; 0.16] | 0.08 [0.04; 0.12] | 0.04 [-0.01; 0.09] | 0.02 [-0.03; 0.07] | 0.01 [-0.04; 0.06] |
| Spain | 0.04 [0.02; 0.06] | 0.02 [-0.01; 0.04] | 0.00 [-0.03; 0.03] | 0.00 [-0.03; 0.03] | 0.00 [-0.03; 0.03] |
| Sweden | 0.10 [0.02; 0.18] | 0.03 [-0.07; 0.13] | 0.01 [-0.11; 0.12] | 0.00 [-0.12; 0.12] | 0.00 [-0.11; 0.11] |
| Switzerland | 0.31 [0.21; 0.41] | 0.23 [0.12; 0.34] | 0.15 [0.03; 0.27] | 0.08 [-0.05; 0.21] | 0.04 [-0.11; 0.18] |
| Taiwan | 0.41 [0.13; 0.68] | 0.37 [0.08; 0.64] | 0.29 [0.00; 0.58] | 0.21 [-0.09; 0.51] | 0.14 [-0.20; 0.47] |
| UK | 0.09 [0.08; 0.10] | 0.05 [0.03; 0.06] | 0.03 [0.01; 0.04] | 0.01 [0.00; 0.03] | 0.01 [-0.01; 0.02] |
| USA | 0.29 [0.28; 0.31] | 0.23 [0.22; 0.25] | 0.16 [0.14; 0.17] | 0.09 [0.08; 0.11] | 0.05 [0.03; 0.07] |
| Overall | 0.26 [0.24; 0.28] | 0.20 [0.18; 0.22] | 0.14 [0.12; 0.16] | 0.09 [0.07; 0.11] | 0.05 [0.03; 0.07] |

*Total refers to ozone-related deaths when levels above 70 μg/m$^3$ (defined as maximum background levels).
**No mortality fractions associated to ozone were found in Australia, as daily ozone levels were below the maximum background level set up at 70 μg/m$^3$.
AQS: air quality standards; WHO: World Health Organization; EU: European Union; NAAQS: National Ambient Air Quality Standard; CAAQS: Chinese Ambient Air Quality Standard

**eTable 4.** Overall and country-specific excess mortality fractions (and 95% confidence interval) associated to ozone between specific thresholds consistent with current air quality standards.

| | Between 70 to 100 μg/m$^3$ | Between 100 to 120 μg/m$^3$ | Between 120 to 140 μg/m$^3$ | Between 140 to 160 μg/m$^3$ | Above 160 μg/m$^3$ |
|---|---|---|---|---|---|
| Australia* | 0 [0;0] | 0 [0;0] | 0 [0;0] | 0 [0;0] | 0 [0;0] |
| Canada | 0.11 [0.09;0.12] | 0.08 [0.05;0.11] | 0.05 [0.01;0.1] | 0.04 [-0.02;0.1] | 0.07 [-0.01;0.14] |
| China | 0.04 [0.01;0.06] | 0.04 [-0.02;0.11] | 0.04 [-0.07;0.15] | 0.04 [-0.11;0.17] | 0.06 [-0.15;0.26] |
| Czech Republic | 0.06 [0.01;0.12] | 0.08 [-0.07;0.21] | 0.06 [-0.14;0.28] | 0.03 [-0.24;0.32] | 0.03 [-0.30;0.34] |
| Estonia | 0.02 [0.01;0.03] | 0.00 [-0.01;0.01] | 0.00 [-0.01;0.01] | 0.00 [-0.01;0.01] | 0.00 [-0.01;0.01] |
| France | 0.06 [0.05;0.08] | 0.06 [0.03;0.09] | 0.04 [-0.01;0.08] | 0.02 [-0.03;0.08] | 0.02 [-0.05;0.08] |
| Germany | 0.04 [0.03;0.05] | 0.04 [0.01;0.06] | 0.02 [-0.01;0.06] | 0.01 [-0.02;0.05] | 0.01 [-0.03;0.05] |
| Greece | 0.05 [-0.03;0.13] | 0.07 [-0.12;0.28] | 0.03 [-0.27;0.30] | 0.01 [-0.31;0.34] | 0.00 [-0.33;0.35] |
| Italy | 0.08 [0.04;0.12] | 0.10 [-0.01;0.21] | 0.06 [-0.10;0.23] | 0.02 [-0.17;0.20] | 0.01 [-0.20;0.21] |
| Japan | 0.10 [0.09;0.11] | 0.09 [0.07;0.12] | 0.07 [0.03;0.11] | 0.03 [-0.02;0.08] | 0.02 [-0.03;0.07] |
| Mexico | 0.02 [0.01;0.03] | 0.04 [0.01;0.07] | 0.06 [-0.01;0.13] | 0.07 [-0.06;0.21] | 0.35 [-0.07;0.75] |
| Portugal | 0.04 [0.00;0.09] | 0.02 [-0.06;0.10] | 0.01 [-0.09;0.11] | 0.00 [-0.10;0.12] | 0.00 [-0.11;0.12] |
| South Africa | 0.09 [0.07;0.12] | 0.08 [0.03;0.13] | 0.06 [-0.03;0.14] | 0.03 [-0.07;0.12] | 0.02 [-0.09;0.13] |
| South Korea | 0.04 [0.03;0.06] | 0.04 [0.01;0.07] | 0.02 [-0.02;0.07] | 0.01 [-0.04;0.06] | 0.01 [-0.04;0.06] |
| Spain | 0.02 [0.01;0.04] | 0.01 [-0.01;0.03] | 0.00 [-0.03;0.03] | 0.00 [-0.03;0.03] | 0.00 [-0.03;0.03] |
| Sweden | 0.07 [0.01;0.13] | 0.03 [-0.07;0.13] | 0.01 [-0.11;0.12] | 0.00 [-0.11;0.12] | 0.00 [-0.11;0.11] |
| Switzerland | 0.08 [0.05;0.10] | 0.08 [0.02;0.14] | 0.07 [-0.03;0.16] | 0.04 [-0.08;0.16] | 0.04 [-0.11;0.18] |
| Taiwan | 0.05 [0.01;0.08] | 0.07 [-0.03;0.16] | 0.08 [-0.09;0.25] | 0.08 [-0.16;0.31] | 0.14 [-0.20;0.47] |
| UK | 0.04 [0.04;0.05] | 0.02 [0.01;0.03] | 0.02 [0.00;0.03] | 0.01 [-0.01;0.03] | 0.01 [-0.01;0.02] |
| USA | 0.06 [0.06;0.06] | 0.07 [0.07;0.08] | 0.06 [0.05;0.08] | 0.04 [0.03;0.06] | 0.05 [0.03;0.07] |
| Overall | 0.06 [0.06;0.06] | 0.06 [0.06;0.07] | 0.05 [0.04;0.06] | 0.04 [0.02;0.05] | 0.05 [0.03;0.07] |

*No mortality fractions associated to ozone were found in Australia, as daily ozone levels were below 70 μg/m$^3$

**eTable 5.** Excess mortality associated to ozone for the total (above 70 µg/m$^3$) and above WHO guideline of 100 µg/m$^3$ in the 406 cities.

| City | Country | Total (Above 70 µg/m$^3$)* | | Above WHO Guideline (100 µg/m$^3$) | |
|---|---|---|---|---|---|
| | | Excess fraction (%, 95% CI) | Annual excess deaths (N, 95% CI) | Excess fraction (%, 95% CI) | Annual excess deaths (N, 95% CI) |
| Brisbane | Australia | 0 [0; 0] | 0 [0; 0] | 0 [0; 0] | 0 [0; 0] |
| Melbourne | Australia | 0 [0; 0] | 0 [0; 0] | 0 [0; 0] | 0 [0; 0] |
| Sydney | Australia | 0 [0; 0] | 0 [0; 0] | 0 [0; 0] | 0 [0; 0] |
| Abbotsford | Canada | 0.18 [0.11; 0.25] | 2 [1; 3] | 0.09 [0.01; 0.17] | 1 [0; 2] |
| Calgary | Canada | 0.3 [0.18; 0.42] | 16 [9; 22] | 0.14 [0.01; 0.28] | 8 [1; 15] |
| Edmonton | Canada | 0.28 [0.18; 0.4] | 17 [11; 25] | 0.16 [0.05; 0.29] | 10 [3; 18] |
| Halifax | Canada | 0.12 [0.07; 0.16] | 3 [2; 5] | 0.05 [-0.01; 0.1] | 1 [0; 3] |
| Hamilton | Canada | 0.4 [0.25; 0.56] | 18 [11; 26] | 0.31 [0.15; 0.48] | 14 [7; 22] |
| Kingston | Canada | 0.33 [0.19; 0.46] | 5 [3; 7] | 0.24 [0.1; 0.37] | 4 [2; 6] |
| Kitchener-Waterloo | Canada | 0.37 [0.22; 0.52] | 11 [6; 15] | 0.27 [0.11; 0.43] | 8 [3; 12] |
| London Ontario | Canada | 0.36 [0.21; 0.5] | 14 [8; 20] | 0.28 [0.12; 0.42] | 11 [4; 16] |
| Montreal | Canada | 0.41 [0.24; 0.58] | 45 [27; 65] | 0.28 [0.11; 0.46] | 31 [12; 51] |
| Niagara | Canada | 0.48 [0.29; 0.66] | 19 [11; 26] | 0.39 [0.2; 0.58] | 16 [8; 23] |
| Oakville | Canada | 0.37 [0.22; 0.53] | 9 [5; 13] | 0.28 [0.12; 0.44] | 6 [3; 10] |
| Oshawa | Canada | 0.3 [0.18; 0.43] | 9 [5; 12] | 0.2 [0.07; 0.33] | 6 [2; 10] |
| Ottawa | Canada | 0.22 [0.13; 0.32] | 12 [7; 18] | 0.13 [0.03; 0.24] | 7 [2; 13] |
| Regina | Canada | 0.08 [0.05; 0.11] | 2 [1; 2] | 0.03 [-0.01; 0.07] | 1 [0; 1] |
| Sarnia | Canada | 0.45 [0.26; 0.64] | 5 [3; 7] | 0.35 [0.15; 0.53] | 4 [2; 6] |
| Sudbury | Canada | 0.28 [0.16; 0.39] | 5 [3; 6] | 0.17 [0.04; 0.28] | 3 [1; 5] |
| Saint John NB | Canada | 0.24 [0.14; 0.34] | 4 [2; 6] | 0.1 [-0.01; 0.21] | 2 [0; 4] |
| St. John's NFL | Canada | 0.08 [0.05; 0.11] | 2 [1; 2] | 0.02 [-0.02; 0.06] | 0 [0; 1] |
| Sault Ste. Marie | Canada | 0.24 [0.14; 0.33] | 3 [2; 4] | 0.13 [0.02; 0.23] | 2 [0; 3] |
| Saskatoon | Canada | 0.08 [0.05; 0.12] | 2 [1; 3] | 0.02 [-0.02; 0.06] | 0 [0; 1] |
| Thunder Bay | Canada | 0.14 [0.08; 0.2] | 2 [1; 3] | 0.05 [-0.02; 0.12] | 1 [0; 2] |

| City | Country | Total (Above 70 μg/m³)* | | Above WHO Guideline (100 μg/m³) | |
|---|---|---|---|---|---|
| | | Excess fraction (%, 95% CI) | Annual excess deaths (N, 95% CI) | Excess fraction (%, 95% CI) | Annual excess deaths (N, 95% CI) |
| Toronto | Canada | 0.59 [0.34; 0.85] | 159 [90; 228] | 0.48 [0.22; 0.73] | 128 [59; 197] |
| Victoria | Canada | 0.13 [0.08; 0.18] | 4 [3; 6] | 0.03 [-0.03; 0.1] | 1 [-1; 3] |
| Vancouver | Canada | 0.21 [0.13; 0.3] | 28 [17; 40] | 0.1 [0.01; 0.2] | 13 [1; 26] |
| Windsor | Canada | 0.42 [0.25; 0.59] | 11 [6; 15] | 0.35 [0.17; 0.52] | 9 [4; 14] |
| Winnipeg | Canada | 0.16 [0.09; 0.23] | 11 [6; 15] | 0.07 [-0.01; 0.15] | 5 [0; 10] |
| Hong Kong | China | 0.02 [0; 0.03] | 6 [1; 11] | 0.01 [-0.01; 0.02] | 2 [-3; 8] |
| Shanghai | China | 0.32 [0.04; 0.57] | 117 [15; 209] | 0.27 [-0.01; 0.53] | 99 [-4; 195] |
| Suzhou | China | 0.15 [0.02; 0.26] | 25 [4; 44] | 0.12 [-0.01; 0.23] | 19 [-2; 39] |
| Prague | Czech Republic | 0.27 [0.02; 0.48] | 38 [3; 69] | 0.2 [-0.06; 0.44] | 29 [-9; 63] |
| Kohtla-Järve linn | Estonia | 0.06 [0.02; 0.1] | 1 [0; 1] | 0.01 [-0.04; 0.06] | 0 [0; 1] |
| Narva linn | Estonia | 0.03 [0.01; 0.05] | 0 [0; 1] | 0 [-0.03; 0.03] | 0 [0; 0] |
| Tallinn | Estonia | 0.01 [0; 0.02] | 1 [0; 1] | 0 [-0.01; 0.01] | 0 [-1; 1] |
| Tartu linn | Estonia | 0.02 [0; 0.04] | 0 [0; 0] | 0 [-0.02; 0.02] | 0 [0; 0] |
| Bordeaux | France | 0.2 [0.07; 0.32] | 10 [4; 17] | 0.12 [-0.01; 0.25] | 6 [-1; 14] |
| Clermont-Ferrand | France | 0.24 [0.09; 0.42] | 5 [2; 8] | 0.15 [-0.02; 0.34] | 3 [0; 6] |
| Dijon | France | 0.21 [0.07; 0.35] | 4 [1; 7] | 0.14 [0; 0.29] | 3 [0; 5] |
| Grenoble | France | 0.26 [0.09; 0.42] | 8 [3; 14] | 0.19 [0.01; 0.35] | 6 [0; 12] |
| Le Havre | France | 0.14 [0.05; 0.22] | 3 [1; 5] | 0.06 [-0.04; 0.16] | 1 [-1; 4] |
| Lille | France | 0.12 [0.05; 0.2] | 11 [4; 18] | 0.08 [-0.01; 0.16] | 7 [-1; 14] |
| Lens-Douai | France | 0.12 [0.04; 0.2] | 4 [1; 7] | 0.07 [-0.02; 0.15] | 3 [-1; 6] |
| Lyon | France | 0.24 [0.09; 0.4] | 19 [7; 31] | 0.17 [0.01; 0.34] | 13 [1; 26] |
| Montpellier | France | 0.33 [0.12; 0.55] | 9 [3; 15] | 0.23 [0; 0.46] | 6 [0; 12] |
| Marseille | France | 0.32 [0.09; 0.54] | 31 [9; 52] | 0.23 [0; 0.47] | 22 [0; 45] |
| Nice | France | 0.43 [0.15; 0.7] | 23 [8; 37] | 0.35 [0.07; 0.64] | 18 [4; 33] |
| Nancy | France | 0.18 [0.06; 0.29] | 5 [2; 8] | 0.12 [0; 0.24] | 3 [0; 7] |
| Nantes | France | 0.19 [0.06; 0.31] | 8 [2; 14] | 0.11 [-0.04; 0.24] | 5 [-2; 11] |

| City | Country | Total (Above 70 µg/m³)* | | Above WHO Guideline (100 µg/m³) | |
|---|---|---|---|---|---|
| | | Excess fraction (%, 95% CI) | Annual excess deaths (N, 95% CI) | Excess fraction (%, 95% CI) | Annual excess deaths (N, 95% CI) |
| Paris | France | 0.15 [0.05; 0.26] | 70 [24; 119] | 0.11 [0; 0.21] | 48 [0; 96] |
| Rennes | France | 0.14 [0.05; 0.23] | 2 [1; 4] | 0.07 [-0.02; 0.17] | 1 [0; 3] |
| Rouen | France | 0.14 [0.04; 0.24] | 6 [2; 10] | 0.08 [-0.03; 0.18] | 3 [-1; 8] |
| Strasbourg | France | 0.22 [0.08; 0.37] | 8 [3; 13] | 0.17 [0.02; 0.32] | 6 [1; 11] |
| Toulouse | France | 0.25 [0.08; 0.42] | 12 [4; 21] | 0.16 [-0.02; 0.33] | 8 [-1; 16] |
| Berlin | Germany | 0.12 [0.04; 0.2] | 46 [14; 74] | 0.08 [-0.01; 0.17] | 30 [-3; 62] |
| Bremen | Germany | 0.1 [0.03; 0.16] | 7 [2; 11] | 0.06 [-0.01; 0.13] | 4 [-1; 9] |
| Dresden | Germany | 0.15 [0.06; 0.25] | 9 [3; 14] | 0.1 [0; 0.2] | 6 [0; 12] |
| Dortmund | Germany | 0.11 [0.04; 0.19] | 8 [3; 13] | 0.08 [0; 0.16] | 6 [0; 11] |
| Duesseldorf | Germany | 0.11 [0.03; 0.19] | 8 [2; 14] | 0.08 [0; 0.16] | 6 [0; 12] |
| Frankfurt | Germany | 0.12 [0.04; 0.2] | 9 [3; 16] | 0.08 [0.01; 0.17] | 6 [0; 13] |
| Hamburg | Germany | 0.08 [0.03; 0.13] | 16 [5; 26] | 0.04 [-0.02; 0.1] | 8 [-3; 20] |
| Hannover | Germany | 0.13 [0.04; 0.22] | 17 [5; 28] | 0.09 [-0.01; 0.18] | 11 [-1; 23] |
| Koeln | Germany | 0.13 [0.05; 0.21] | 13 [5; 22] | 0.09 [0.01; 0.19] | 10 [1; 19] |
| Leipzig | Germany | 0.16 [0.05; 0.27] | 11 [4; 19] | 0.11 [0; 0.22] | 8 [0; 15] |
| Muenchen | Germany | 0.14 [0.04; 0.23] | 18 [5; 30] | 0.09 [-0.01; 0.19] | 12 [-2; 25] |
| Stuttgart | Germany | 0.17 [0.06; 0.28] | 10 [4; 17] | 0.12 [0.01; 0.24] | 8 [1; 15] |
| Athens | Greece | 0.16 [-0.07; 0.41] | 52 [-23; 132] | 0.11 [-0.13; 0.37] | 35 [-42; 117] |
| Cagliari | Italy | 0.04 [0.01; 0.08] | 1 [0; 1] | 0 [-0.05; 0.06] | 0 [-1; 1] |
| Florence | Italy | 0.32 [0.03; 0.58] | 16 [2; 28] | 0.25 [-0.03; 0.52] | 12 [-1; 25] |
| Frosinone | Italy | 0.4 [0.07; 0.72] | 2 [0; 3] | 0.32 [-0.02; 0.66] | 1 [0; 3] |
| Genoa | Italy | 0.29 [0.04; 0.52] | 27 [4; 49] | 0.21 [-0.04; 0.46] | 20 [-4; 44] |
| Latina | Italy | 0.25 [0.04; 0.47] | 2 [0; 5] | 0.14 [-0.09; 0.38] | 1 [-1; 4] |
| Milan | Italy | 0.24 [0.03; 0.46] | 33 [4; 61] | 0.19 [-0.02; 0.41] | 26 [-2; 55] |
| Rieti | Italy | 0.28 [0.04; 0.51] | 1 [0; 2] | 0.2 [-0.06; 0.45] | 1 [0; 2] |
| Rome | Italy | 0.27 [0.05; 0.52] | 69 [13; 132] | 0.19 [-0.05; 0.44] | 48 [-12; 111] |

| City | Country | Total (Above 70 μg/m$^3$)* | | Above WHO Guideline (100 μg/m$^3$) | |
|---|---|---|---|---|---|
| | | Excess fraction (%, 95% CI) | Annual excess deaths (N, 95% CI) | Excess fraction (%, 95% CI) | Annual excess deaths (N, 95% CI) |
| Trieste | Italy | 0.32 [0.03; 0.58] | 10 [1; 19] | 0.21 [-0.09; 0.49] | 7 [-3; 16] |
| Aikita | Japan | 0.35 [0.18; 0.53] | 15 [8; 23] | 0.21 [0.02; 0.4] | 9 [1; 17] |
| Aomori | Japan | 0.23 [0.12; 0.35] | 10 [5; 15] | 0.12 [-0.01; 0.25] | 5 [0; 11] |
| Chiba | Japan | 0.3 [0.15; 0.46] | 29 [14; 44] | 0.21 [0.05; 0.37] | 20 [5; 35] |
| Fukushima | Japan | 0.26 [0.13; 0.39] | 10 [5; 15] | 0.16 [0.01; 0.31] | 6 [0; 12] |
| Fukuoka | Japan | 0.37 [0.18; 0.56] | 50 [25; 75] | 0.26 [0.06; 0.45] | 35 [8; 60] |
| Fukui | Japan | 0.42 [0.2; 0.64] | 14 [7; 21] | 0.3 [0.08; 0.52] | 10 [3; 18] |
| Gifu | Japan | 0.38 [0.19; 0.58] | 20 [10; 30] | 0.28 [0.09; 0.48] | 14 [5; 25] |
| Hiroshima | Japan | 0.37 [0.19; 0.55] | 44 [23; 66] | 0.26 [0.08; 0.45] | 32 [10; 54] |
| Kagoshima | Japan | 0.2 [0.1; 0.3] | 15 [7; 22] | 0.1 [-0.02; 0.21] | 7 [-1; 15] |
| Kumamoto | Japan | 0.39 [0.21; 0.59] | 32 [17; 48] | 0.28 [0.1; 0.49] | 23 [8; 40] |
| Kanazawa | Japan | 0.44 [0.23; 0.65] | 23 [12; 34] | 0.3 [0.07; 0.5] | 16 [4; 27] |
| Kobe | Japan | 0.33 [0.16; 0.5] | 61 [30; 91] | 0.23 [0.07; 0.4] | 43 [12; 74] |
| Kochi | Japan | 0.34 [0.17; 0.5] | 15 [8; 22] | 0.21 [0.03; 0.39] | 9 [2; 17] |
| Kofu | Japan | 0.37 [0.18; 0.56] | 10 [5; 15] | 0.27 [0.07; 0.46] | 7 [2; 12] |
| Kyoto | Japan | 0.36 [0.18; 0.54] | 62 [30; 93] | 0.25 [0.07; 0.44] | 44 [12; 75] |
| Matsue | Japan | 0.52 [0.27; 0.78] | 14 [7; 22] | 0.37 [0.11; 0.64] | 10 [3; 18] |
| Maebashi | Japan | 0.47 [0.24; 0.7] | 21 [11; 30] | 0.37 [0.13; 0.62] | 16 [6; 27] |
| Mito | Japan | 0.36 [0.17; 0.55] | 11 [6; 17] | 0.25 [0.05; 0.44] | 8 [2; 14] |
| Morioka | Japan | 0.21 [0.11; 0.32] | 7 [4; 11] | 0.12 [0; 0.23] | 4 [0; 8] |
| Matsuyama | Japan | 0.27 [0.14; 0.41] | 17 [9; 26] | 0.17 [0.03; 0.31] | 11 [2; 19] |
| Nagano | Japan | 0.3 [0.15; 0.44] | 15 [7; 22] | 0.19 [0.05; 0.34] | 9 [2; 17] |
| Nagoya | Japan | 0.36 [0.19; 0.54] | 90 [47; 136] | 0.26 [0.07; 0.44] | 65 [18; 111] |
| Naha | Japan | 0.17 [0.09; 0.26] | 5 [3; 8] | 0.08 [-0.01; 0.17] | 2 [0; 5] |
| Nara | Japan | 0.35 [0.18; 0.52] | 15 [7; 22] | 0.24 [0.07; 0.42] | 10 [3; 18] |
| Nagasaki | Japan | 0.28 [0.14; 0.41] | 17 [8; 25] | 0.17 [0.02; 0.31] | 10 [1; 19] |

| City | Country | Total (Above 70 µg/m$^3$)* | | Above WHO Guideline (100 µg/m$^3$) | |
|---|---|---|---|---|---|
| | | Excess fraction (%, 95% CI) | Annual excess deaths (N, 95% CI) | Excess fraction (%, 95% CI) | Annual excess deaths (N, 95% CI) |
| Niigata | Japan | 0.36 [0.19; 0.52] | 36 [19; 53] | 0.21 [0.04; 0.4] | 22 [4; 40] |
| Oita | Japan | 0.22 [0.12; 0.33] | 11 [6; 16] | 0.12 [0; 0.24] | 6 [0; 12] |
| Okayama | Japan | 0.31 [0.16; 0.45] | 25 [12; 36] | 0.21 [0.06; 0.37] | 17 [5; 29] |
| Osaka | Japan | 0.31 [0.16; 0.47] | 104 [53; 158] | 0.22 [0.06; 0.39] | 74 [20; 131] |
| Otsu | Japan | 0.38 [0.18; 0.58] | 13 [6; 20] | 0.27 [0.06; 0.47] | 9 [2; 16] |
| Saga | Japan | 0.39 [0.2; 0.58] | 12 [6; 18] | 0.28 [0.09; 0.47] | 9 [3; 15] |
| Saitama | Japan | 0.37 [0.19; 0.55] | 44 [23; 65] | 0.28 [0.1; 0.47] | 33 [11; 55] |
| Sendai | Japan | 0.26 [0.13; 0.39] | 27 [13; 40] | 0.16 [0.01; 0.3] | 16 [1; 30] |
| Shizuoka | Japan | 0.38 [0.18; 0.56] | 35 [16; 52] | 0.28 [0.07; 0.47] | 26 [6; 44] |
| Sapporo | Japan | 0.14 [0.08; 0.21] | 30 [16; 44] | 0.06 [-0.02; 0.14] | 13 [-4; 30] |
| Takamatsu | Japan | 0.3 [0.14; 0.46] | 16 [8; 24] | 0.19 [0.02; 0.35] | 10 [1; 18] |
| Tokushima | Japan | 0.37 [0.19; 0.54] | 13 [6; 19] | 0.27 [0.08; 0.45] | 9 [3; 16] |
| Tokyo | Japan | 0.27 [0.14; 0.4] | 249 [127; 371] | 0.18 [0.04; 0.32] | 170 [40; 304] |
| Toyama | Japan | 0.4 [0.2; 0.59] | 22 [11; 33] | 0.27 [0.06; 0.47] | 15 [3; 26] |
| Tsu | Japan | 0.42 [0.21; 0.62] | 16 [8; 23] | 0.31 [0.08; 0.51] | 12 [3; 19] |
| Utsunomiya | Japan | 0.38 [0.21; 0.57] | 21 [11; 31] | 0.29 [0.1; 0.48] | 16 [6; 26] |
| Wakayama | Japan | 0.37 [0.2; 0.55] | 20 [11; 30] | 0.27 [0.09; 0.46] | 14 [5; 25] |
| Yokohama | Japan | 0.3 [0.16; 0.44] | 111 [60; 162] | 0.23 [0.09; 0.38] | 86 [31; 140] |
| Yamaguchi | Japan | 0.33 [0.16; 0.48] | 9 [4; 13] | 0.22 [0.05; 0.39] | 6 [1; 10] |
| Yamagata | Japan | 0.3 [0.16; 0.45] | 10 [5; 15] | 0.19 [0.04; 0.35] | 6 [1; 12] |
| Busan | South Korea | 0.12 [0.02; 0.21] | 25 [5; 42] | 0.07 [-0.04; 0.17] | 13 [-9; 33] |
| Daegu | South Korea | 0.18 [0.05; 0.3] | 22 [7; 38] | 0.13 [0.01; 0.26] | 16 [1; 32] |
| Daejeon | South Korea | 0.15 [0.03; 0.26] | 10 [2; 17] | 0.11 [0; 0.22] | 7 [0; 14] |
| Gwangju | South Korea | 0.14 [0.04; 0.25] | 9 [3; 16] | 0.1 [-0.01; 0.2] | 6 [-1; 13] |
| Incheon | South Korea | 0.12 [0.04; 0.2] | 14 [4; 24] | 0.07 [-0.02; 0.16] | 9 [-2; 20] |
| Seoul | South Korea | 0.1 [0.03; 0.17] | 41 [13; 71] | 0.06 [-0.01; 0.14] | 27 [-3; 58] |

| City | Country | Total (Above 70 μg/m³)* | | Above WHO Guideline (100 μg/m³) | |
|---|---|---|---|---|---|
| | | Excess fraction (%, 95% CI) | Annual excess deaths (N, 95% CI) | Excess fraction (%, 95% CI) | Annual excess deaths (N, 95% CI) |
| Ulsan | South Korea | 0.14 [0.05; 0.24] | 6 [2; 11] | 0.09 [-0.02; 0.2] | 4 [-1; 9] |
| Guadalajara | Mexico | 0.42 [0.04; 0.8] | 93 [10; 179] | 0.38 [0.01; 0.78] | 85 [2; 173] |
| Leon | Mexico | 0.32 [0.01; 0.62] | 25 [1; 48] | 0.28 [-0.04; 0.58] | 22 [-3; 45] |
| Monterrey | Mexico | 0.25 [0.01; 0.48] | 45 [3; 87] | 0.2 [-0.03; 0.44] | 37 [-6; 80] |
| Puebla-Tlaxcala | Mexico | 0.23 [0.01; 0.45] | 30 [2; 59] | 0.19 [-0.03; 0.42] | 25 [-4; 55] |
| Tijuana | Mexico | 0.09 [0; 0.17] | 6 [0; 11] | 0.04 [-0.06; 0.14] | 2 [-4; 9] |
| Toluca de Lerdo | Mexico | 0.33 [0.01; 0.69] | 27 [1; 57] | 0.3 [-0.03; 0.66] | 24 [-2; 54] |
| Valley of Mexico | Mexico | 0.73 [0.04; 1.38] | 707 [39; 1339] | 0.72 [0.02; 1.36] | 694 [22; 1317] |
| Lisboa | Portugal | 0.09 [-0.03; 0.2] | 20 [-6; 45] | 0.04 [-0.09; 0.17] | 9 [-20; 39] |
| Porto | Portugal | 0.07 [-0.02; 0.15] | 10 [-3; 23] | 0.03 [-0.06; 0.12] | 5 [-10; 19] |
| City of Johannesburg | South Africa | 0.32 [0.15; 0.49] | 121 [59; 187] | 0.22 [0.05; 0.39] | 82 [19; 148] |
| eThekwini | South Africa | 0.14 [0.06; 0.22] | 56 [25; 85] | 0.08 [0; 0.17] | 33 [0; 66] |
| Gert Sibande | South Africa | 0.54 [0.27; 0.83] | 79 [39; 121] | 0.37 [0.08; 0.68] | 54 [11; 99] |
| Nkangala | South Africa | 0.43 [0.22; 0.68] | 65 [32; 101] | 0.33 [0.1; 0.58] | 50 [15; 87] |
| Sedibeng | South Africa | 0.33 [0.15; 0.5] | 43 [20; 64] | 0.19 [-0.01; 0.37] | 24 [-2; 48] |
| A Coruna | Spain | 0.03 [-0.04; 0.1] | 1 [-1; 3] | 0.01 [-0.07; 0.1] | 0 [-2; 2] |
| Albacete | Spain | 0.1 [-0.13; 0.35] | 1 [-2; 4] | 0.07 [-0.19; 0.33] | 1 [-2; 4] |
| Alicante | Spain | 0.06 [-0.08; 0.18] | 2 [-2; 5] | 0.01 [-0.17; 0.18] | 0 [-5; 5] |
| Almeria | Spain | 0.07 [-0.08; 0.23] | 1 [-1; 3] | 0.03 [-0.15; 0.23] | 0 [-2; 3] |
| Avila | Spain | 0.04 [-0.06; 0.13] | 0 [0; 1] | 0.01 [-0.11; 0.11] | 0 [-1; 1] |
| Badajoz | Spain | 0.02 [-0.02; 0.06] | 0 [0; 1] | 0 [-0.05; 0.05] | 0 [-1; 1] |
| Bilbao | Spain | 0.01 [-0.02; 0.04] | 0 [-1; 2] | 0 [-0.04; 0.04] | 0 [-1; 2] |
| Barcelona | Spain | 0.02 [-0.03; 0.08] | 4 [-5; 13] | 0 [-0.07; 0.07] | 1 [-11; 13] |
| Burgos | Spain | 0.07 [-0.09; 0.25] | 1 [-2; 4] | 0.04 [-0.15; 0.23] | 1 [-2; 4] |
| Cadiz | Spain | 0.08 [-0.11; 0.27] | 1 [-1; 4] | 0.04 [-0.17; 0.25] | 0 [-2; 3] |
| Caceres | Spain | 0.02 [-0.02; 0.05] | 0 [0; 0] | 0 [-0.04; 0.05] | 0 [0; 0] |

| City | Country | Total (Above 70 μg/m³)* | | Above WHO Guideline (100 μg/m³) | |
|---|---|---|---|---|---|
| | | Excess fraction (%, 95% CI) | Annual excess deaths (N, 95% CI) | Excess fraction (%, 95% CI) | Annual excess deaths (N, 95% CI) |
| Ciudad Real | Spain | 0.1 [-0.15; 0.32] | 1 [-1; 2] | 0.06 [-0.19; 0.3] | 0 [-1; 2] |
| Ceuta | Spain | 0.09 [-0.11; 0.29] | 0 [-1; 2] | 0.05 [-0.17; 0.27] | 0 [-1; 1] |
| Cordoba | Spain | 0.06 [-0.08; 0.2] | 2 [-2; 5] | 0.02 [-0.15; 0.19] | 0 [-4; 5] |
| Castellon | Spain | 0.01 [-0.01; 0.04] | 0 [0; 0] | 0 [-0.04; 0.04] | 0 [0; 1] |
| Cuenca | Spain | 0.09 [-0.12; 0.29] | 1 [-1; 2] | 0.06 [-0.16; 0.27] | 0 [-1; 2] |
| Guadalajara | Spain | 0.11 [-0.15; 0.39] | 1 [-1; 2] | 0.09 [-0.18; 0.37] | 1 [-1; 2] |
| Granada | Spain | 0.05 [-0.07; 0.15] | 1 [-2; 3] | 0.01 [-0.12; 0.13] | 0 [-3; 3] |
| Huesca | Spain | 0.09 [-0.1; 0.32] | 1 [-1; 2] | 0.06 [-0.14; 0.31] | 0 [-1; 2] |
| Jaen | Spain | 0.12 [-0.14; 0.41] | 1 [-1; 4] | 0.09 [-0.19; 0.39] | 1 [-2; 4] |
| Leon | Spain | 0.03 [-0.04; 0.1] | 0 [-1; 1] | 0.01 [-0.08; 0.09] | 0 [-1; 1] |
| Lleida | Spain | 0.07 [-0.09; 0.21] | 1 [-1; 3] | 0.04 [-0.12; 0.19] | 0 [-1; 2] |
| Lugo | Spain | 0.02 [-0.03; 0.08] | 0 [0; 1] | 0.01 [-0.06; 0.07] | 0 [-1; 1] |
| Malaga | Spain | 0.06 [-0.09; 0.21] | 3 [-4; 10] | 0.02 [-0.17; 0.21] | 1 [-8; 10] |
| Madrid | Spain | 0.03 [-0.04; 0.11] | 9 [-12; 31] | 0.01 [-0.07; 0.1] | 3 [-21; 27] |
| Melilla | Spain | 0.1 [-0.12; 0.34] | 0 [-1; 2] | 0.08 [-0.15; 0.33] | 0 [-1; 2] |
| Murcia | Spain | 0.08 [-0.1; 0.27] | 3 [-3; 9] | 0.05 [-0.15; 0.26] | 2 [-5; 9] |
| Ourense | Spain | 0.05 [-0.07; 0.16] | 1 [-1; 2] | 0.03 [-0.11; 0.15] | 0 [-1; 2] |
| Oviedo | Spain | 0.02 [-0.03; 0.08] | 1 [-1; 2] | 0.01 [-0.06; 0.07] | 0 [-1; 2] |
| Palmas G. Canaria | Spain | 0 [0; 0.01] | 0 [0; 0] | 0 [-0.01; 0.01] | 0 [0; 0] |
| Palma Mallorca | Spain | 0.04 [-0.05; 0.13] | 1 [-1; 4] | 0.01 [-0.1; 0.12] | 0 [-3; 4] |
| Palencia | Spain | 0.03 [-0.04; 0.09] | 0 [0; 1] | 0 [-0.08; 0.09] | 0 [-1; 1] |
| Pamplona | Spain | 0.04 [-0.04; 0.11] | 1 [-1; 2] | 0.01 [-0.08; 0.11] | 0 [-1; 2] |
| Segovia | Spain | 0.04 [-0.06; 0.13] | 0 [0; 1] | 0.01 [-0.12; 0.13] | 0 [-1; 1] |
| Salamanca | Spain | 0.02 [-0.03; 0.08] | 0 [0; 1] | 0 [-0.06; 0.08] | 0 [-1; 1] |
| San Sebastian | Spain | 0.02 [-0.02; 0.05] | 0 [0; 1] | 0 [-0.05; 0.05] | 0 [-1; 1] |
| Santander | Spain | 0.01 [-0.01; 0.04] | 0 [0; 1] | 0 [-0.03; 0.03] | 0 [-1; 1] |

| City | Country | Total (Above 70 μg/m$^3$)* | | Above WHO Guideline (100 μg/m$^3$) | |
|---|---|---|---|---|---|
| | | Excess fraction (%, 95% CI) | Annual excess deaths (N, 95% CI) | Excess fraction (%, 95% CI) | Annual excess deaths (N, 95% CI) |
| Soria | Spain | 0.04 [-0.05; 0.15] | 0 [0; 1] | 0.02 [-0.1; 0.14] | 0 [0; 1] |
| Sevilla | Spain | 0.08 [-0.11; 0.27] | 5 [-7; 17] | 0.05 [-0.14; 0.25] | 3 [-9; 16] |
| Teruel | Spain | 0.09 [-0.12; 0.28] | 0 [0; 1] | 0.05 [-0.19; 0.27] | 0 [-1; 1] |
| Tenerife | Spain | 0.05 [-0.07; 0.16] | 1 [-1; 3] | 0.01 [-0.14; 0.15] | 0 [-3; 3] |
| Toledo | Spain | 0.09 [-0.12; 0.29] | 0 [-1; 2] | 0.04 [-0.19; 0.26] | 0 [-1; 1] |
| Tarragona | Spain | 0.08 [-0.11; 0.24] | 1 [-1; 2] | 0.04 [-0.17; 0.23] | 0 [-2; 2] |
| Vitoria | Spain | 0.04 [-0.04; 0.14] | 1 [-1; 3] | 0.01 [-0.08; 0.13] | 0 [-2; 2] |
| Valladolid | Spain | 0.07 [-0.09; 0.23] | 2 [-3; 7] | 0.04 [-0.14; 0.23] | 1 [-4; 7] |
| Valencia | Spain | 0.02 [-0.02; 0.05] | 1 [-1; 4] | 0 [-0.04; 0.04] | 0 [-3; 3] |
| Zamora | Spain | 0.03 [-0.04; 0.09] | 0 [0; 1] | 0 [-0.09; 0.09] | 0 [-1; 1] |
| Zaragoza | Spain | 0.01 [-0.01; 0.03] | 1 [-1; 2] | 0 [-0.03; 0.03] | 0 [-2; 2] |
| Basel | Switzerland | 0.28 [0.05; 0.5] | 6 [1; 10] | 0.21 [-0.04; 0.43] | 4 [-1; 9] |
| Bern | Switzerland | 0.28 [0.06; 0.54] | 4 [1; 8] | 0.2 [-0.03; 0.46] | 3 [0; 7] |
| Geneva | Switzerland | 0.2 [0.05; 0.36] | 3 [1; 5] | 0.13 [-0.03; 0.31] | 2 [0; 4] |
| Lausanne | Switzerland | 0.29 [0.06; 0.52] | 3 [1; 6] | 0.21 [-0.04; 0.46] | 2 [0; 5] |
| Lugano | Switzerland | 0.45 [0.1; 0.82] | 7 [2; 13] | 0.4 [0.04; 0.78] | 6 [1; 12] |
| Luzern | Switzerland | 0.26 [0.04; 0.46] | 2 [0; 4] | 0.19 [-0.03; 0.4] | 2 [0; 3] |
| St. Gallen | Switzerland | 0.39 [0.08; 0.7] | 3 [1; 5] | 0.27 [-0.07; 0.59] | 2 [-1; 4] |
| Zurich | Switzerland | 0.31 [0.05; 0.54] | 13 [2; 22] | 0.23 [-0.02; 0.48] | 10 [-1; 20] |
| Stockholm | Sweden | 0.1 [0.02; 0.18] | 10 [2; 18] | 0.03 [-0.07; 0.13] | 3 [-7; 13] |
| Kaohsiung | Taiwan | 0.57 [-0.08; 1.19] | 115 [-16; 237] | 0.55 [-0.1; 1.16] | 109 [-20; 231] |
| Taipei | Taiwan | 0.34 [-0.05; 0.72] | 131 [-21; 276] | 0.28 [-0.11; 0.67] | 109 [-43; 258] |
| Taichung | Taiwan | 0.38 [-0.01; 0.8] | 59 [-2; 125] | 0.33 [-0.05; 0.75] | 52 [-8; 117] |
| Bristol | UK | 0.09 [0.06; 0.12] | 5 [4; 7] | 0.03 [0; 0.06] | 2 [0; 4] |
| Cardiff | UK | 0.12 [0.08; 0.15] | 4 [3; 5] | 0.07 [0.03; 0.1] | 2 [1; 4] |
| Greater London | UK | 0.1 [0.07; 0.12] | 63 [44; 81] | 0.06 [0.02; 0.09] | 37 [15; 57] |

| City | Country | Total (Above 70 μg/m$^3$)* | | Above WHO Guideline (100 μg/m$^3$) | |
|---|---|---|---|---|---|
| | | Excess fraction (%, 95% CI) | Annual excess deaths (N, 95% CI) | Excess fraction (%, 95% CI) | Annual excess deaths (N, 95% CI) |
| Greater Manchester | UK | 0.06 [0.04; 0.08] | 11 [7; 14] | 0.03 [0.01; 0.05] | 5 [2; 9] |
| Kingston upon Hull | UK | 0.06 [0.04; 0.08] | 2 [2; 3] | 0.03 [0; 0.05] | 1 [0; 2] |
| Leicester | UK | 0.15 [0.1; 0.2] | 7 [5; 9] | 0.09 [0.04; 0.14] | 4 [2; 6] |
| Liverpool | UK | 0.07 [0.05; 0.09] | 6 [4; 8] | 0.02 [-0.01; 0.04] | 2 [0; 4] |
| Norwich | UK | 0.21 [0.15; 0.27] | 5 [3; 6] | 0.12 [0.05; 0.19] | 3 [1; 4] |
| Nottingham | UK | 0.06 [0.04; 0.08] | 3 [2; 4] | 0.03 [0.01; 0.05] | 2 [1; 3] |
| Sheffield | UK | 0.06 [0.04; 0.08] | 4 [3; 5] | 0.03 [0; 0.05] | 2 [0; 3] |
| Southampton | UK | 0.07 [0.05; 0.1] | 2 [1; 3] | 0.03 [0; 0.06] | 1 [0; 2] |
| The Potteries | UK | 0.13 [0.09; 0.17] | 6 [4; 8] | 0.06 [0.02; 0.11] | 3 [1; 5] |
| Tyneside | UK | 0.08 [0.06; 0.11] | 6 [4; 8] | 0.02 [-0.01; 0.05] | 2 [-1; 4] |
| West Midlands | UK | 0.11 [0.08; 0.14] | 28 [20; 37] | 0.06 [0.02; 0.1] | 15 [6; 25] |
| West Yorkshire | UK | 0.05 [0.03; 0.06] | 5 [4; 7] | 0.02 [0; 0.04] | 2 [0; 4] |
| Augusta | USA | 0.46 [0.27; 0.62] | 8 [5; 11] | 0.38 [0.18; 0.55] | 7 [3; 10] |
| Akron | USA | 0.43 [0.26; 0.59] | 22 [13; 30] | 0.36 [0.2; 0.53] | 19 [10; 27] |
| Albany | USA | 0.19 [0.11; 0.26] | 5 [3; 8] | 0.13 [0.05; 0.21] | 4 [1; 6] |
| Albuquerque | USA | 0.28 [0.17; 0.4] | 10 [6; 14] | 0.19 [0.06; 0.31] | 7 [2; 11] |
| Allentown | USA | 0.36 [0.22; 0.5] | 10 [6; 15] | 0.29 [0.15; 0.44] | 9 [4; 13] |
| Anaheim | USA | 0.28 [0.16; 0.4] | 42 [25; 60] | 0.2 [0.08; 0.32] | 30 [12; 48] |
| Ann Arbor | USA | 0.39 [0.25; 0.55] | 6 [4; 9] | 0.3 [0.15; 0.45] | 5 [2; 7] |
| Annandale | USA | 0.29 [0.18; 0.41] | 10 [6; 14] | 0.24 [0.13; 0.37] | 8 [4; 12] |
| Austin | USA | 0.3 [0.18; 0.42] | 10 [6; 14] | 0.22 [0.09; 0.35] | 7 [3; 11] |
| Atlantic City | USA | 0.33 [0.19; 0.46] | 8 [5; 11] | 0.26 [0.13; 0.39] | 6 [3; 9] |
| Atlanta | USA | 0.52 [0.32; 0.71] | 77 [47; 105] | 0.45 [0.24; 0.65] | 67 [35; 96] |
| Atzec | USA | 0.38 [0.22; 0.53] | 2 [1; 2] | 0.28 [0.13; 0.44] | 1 [1; 2] |
| Buffalo | USA | 0.24 [0.15; 0.34] | 24 [15; 34] | 0.19 [0.09; 0.28] | 19 [9; 28] |
| Bakersfield | USA | 0.63 [0.38; 0.89] | 27 [16; 38] | 0.58 [0.33; 0.84] | 25 [14; 36] |

| City | Country | Total (Above 70 μg/m$^3$)* | | Above WHO Guideline (100 μg/m$^3$) | |
|---|---|---|---|---|---|
| | | Excess fraction (%, 95% CI) | Annual excess deaths (N, 95% CI) | Excess fraction (%, 95% CI) | Annual excess deaths (N, 95% CI) |
| Boulder | USA | 0.28 [0.17; 0.4] | 3 [2; 5] | 0.2 [0.08; 0.32] | 2 [1; 4] |
| Baltimore | USA | 0.3 [0.18; 0.42] | 45 [28; 64] | 0.25 [0.13; 0.37] | 38 [20; 57] |
| Bangor | USA | 0.22 [0.14; 0.32] | 3 [2; 4] | 0.13 [0.04; 0.23] | 2 [0; 3] |
| Bergen | USA | 0.21 [0.12; 0.29] | 23 [14; 33] | 0.16 [0.08; 0.24] | 19 [10; 28] |
| Burlington | USA | 0.25 [0.15; 0.35] | 2 [1; 3] | 0.16 [0.05; 0.27] | 1 [0; 2] |
| Birmingham | USA | 0.35 [0.22; 0.49] | 29 [18; 40] | 0.28 [0.14; 0.42] | 23 [11; 34] |
| Barnstable | USA | 0.41 [0.26; 0.58] | 10 [7; 14] | 0.32 [0.16; 0.5] | 8 [4; 12] |
| Brownsville | USA | 0.13 [0.08; 0.18] | 2 [1; 3] | 0.07 [0.01; 0.13] | 1 [0; 2] |
| Boston | USA | 0.16 [0.1; 0.23] | 37 [23; 52] | 0.12 [0.06; 0.19] | 27 [13; 43] |
| Baton Rouge | USA | 0.27 [0.17; 0.38] | 8 [5; 11] | 0.21 [0.1; 0.32] | 6 [3; 10] |
| Cedar Rapids | USA | 0.14 [0.09; 0.2] | 2 [1; 3] | 0.08 [0.02; 0.15] | 1 [0; 2] |
| Chicago | USA | 0.14 [0.08; 0.19] | 73 [44; 101] | 0.1 [0.04; 0.15] | 51 [21; 80] |
| Charlotte | USA | 0.55 [0.32; 0.77] | 22 [13; 30] | 0.48 [0.26; 0.7] | 19 [10; 28] |
| Charleston SC | USA | 0.23 [0.13; 0.33] | 6 [3; 8] | 0.16 [0.05; 0.26] | 4 [1; 6] |
| Chattanoga | USA | 0.42 [0.26; 0.59] | 12 [7; 17] | 0.35 [0.18; 0.52] | 10 [5; 15] |
| Charleston WV | USA | 0.34 [0.2; 0.47] | 8 [5; 11] | 0.28 [0.15; 0.42] | 7 [3; 10] |
| Columbus | USA | 0.41 [0.24; 0.57] | 31 [18; 43] | 0.33 [0.16; 0.49] | 25 [12; 37] |
| Colorado Springs | USA | 0.25 [0.15; 0.34] | 6 [4; 8] | 0.15 [0.05; 0.25] | 4 [1; 6] |
| Cleveland | USA | 0.34 [0.2; 0.46] | 64 [38; 89] | 0.27 [0.13; 0.4] | 52 [25; 76] |
| Cincinnati | USA | 0.41 [0.25; 0.57] | 34 [20; 47] | 0.34 [0.17; 0.51] | 28 [14; 41] |
| Canton | USA | 0.47 [0.28; 0.66] | 17 [10; 24] | 0.39 [0.2; 0.59] | 15 [8; 22] |
| Columbia | USA | 0.34 [0.2; 0.47] | 12 [7; 17] | 0.28 [0.13; 0.42] | 10 [5; 15] |
| Corpus Christi | USA | 0.2 [0.12; 0.28] | 4 [3; 6] | 0.14 [0.05; 0.22] | 3 [1; 5] |
| Davis | USA | 0.5 [0.31; 0.71] | 4 [3; 6] | 0.41 [0.21; 0.62] | 4 [2; 5] |
| Dallas | USA | 0.29 [0.17; 0.4] | 36 [22; 50] | 0.23 [0.12; 0.35] | 29 [14; 43] |
| Denver | USA | 0.28 [0.16; 0.38] | 24 [14; 33] | 0.2 [0.08; 0.31] | 18 [7; 27] |

| City | Country | Total (Above 70 µg/m$^3$)* | | Above WHO Guideline (100 µg/m$^3$) | |
|---|---|---|---|---|---|
| | | Excess fraction (%, 95% CI) | Annual excess deaths (N, 95% CI) | Excess fraction (%, 95% CI) | Annual excess deaths (N, 95% CI) |
| Dodge | USA | 0.3 [0.18; 0.41] | 2 [1; 2] | 0.21 [0.09; 0.33] | 1 [0; 2] |
| Dover | USA | 0.49 [0.3; 0.69] | 5 [3; 7] | 0.41 [0.22; 0.63] | 4 [2; 6] |
| Durham | USA | 0.54 [0.34; 0.76] | 9 [6; 13] | 0.46 [0.26; 0.68] | 8 [4; 11] |
| Des Moines | USA | 0.08 [0.05; 0.12] | 2 [1; 3] | 0.04 [0; 0.08] | 1 [0; 2] |
| Detroit | USA | 0.28 [0.17; 0.39] | 97 [58; 136] | 0.22 [0.1; 0.33] | 76 [34; 116] |
| Davenport | USA | 0.29 [0.17; 0.4] | 8 [4; 11] | 0.21 [0.09; 0.33] | 6 [2; 9] |
| Daytona Beach | USA | 0.22 [0.12; 0.3] | 12 [7; 17] | 0.13 [0.04; 0.22] | 7 [2; 12] |
| Dayton | USA | 0.44 [0.27; 0.63] | 23 [14; 33] | 0.36 [0.2; 0.55] | 19 [10; 28] |
| El Centro | USA | 0.37 [0.23; 0.53] | 3 [2; 4] | 0.28 [0.12; 0.44] | 2 [1; 3] |
| Elkhart | USA | 0.44 [0.27; 0.61] | 6 [4; 8] | 0.33 [0.16; 0.51] | 5 [2; 7] |
| El Paso | USA | 0.25 [0.15; 0.35] | 9 [5; 12] | 0.16 [0.06; 0.26] | 6 [2; 9] |
| Elizabeth | USA | 0.25 [0.15; 0.35] | 13 [8; 17] | 0.21 [0.1; 0.31] | 10 [5; 15] |
| Erie | USA | 0.35 [0.21; 0.49] | 9 [5; 13] | 0.28 [0.13; 0.42] | 7 [3; 11] |
| Essex | USA | 0.21 [0.13; 0.29] | 13 [8; 18] | 0.14 [0.05; 0.23] | 9 [3; 14] |
| Eugene | USA | 0.2 [0.12; 0.27] | 5 [3; 7] | 0.12 [0.04; 0.21] | 3 [1; 5] |
| Evansville | USA | 0.56 [0.35; 0.77] | 10 [6; 14] | 0.48 [0.27; 0.69] | 9 [5; 12] |
| Fargo | USA | 0.14 [0.09; 0.21] | 1 [1; 2] | 0.06 [0; 0.13] | 0 [0; 1] |
| Flint | USA | 0.36 [0.21; 0.51] | 13 [8; 18] | 0.27 [0.12; 0.43] | 10 [4; 15] |
| Fresno | USA | 0.57 [0.35; 0.8] | 28 [18; 40] | 0.52 [0.3; 0.76] | 26 [15; 38] |
| Fort Lauderdale | USA | 0.13 [0.08; 0.18] | 18 [12; 26] | 0.07 [0.01; 0.12] | 10 [2; 18] |
| Fort Myers | USA | 0.2 [0.12; 0.29] | 8 [5; 12] | 0.12 [0.03; 0.21] | 5 [1; 9] |
| Fort Pierce | USA | 0.19 [0.12; 0.27] | 7 [5; 10] | 0.11 [0.03; 0.19] | 4 [1; 7] |
| Fort Worth | USA | 0.35 [0.22; 0.49] | 29 [18; 40] | 0.29 [0.15; 0.43] | 24 [12; 36] |
| Fort Wayne | USA | 0.5 [0.28; 0.7] | 12 [7; 17] | 0.41 [0.19; 0.6] | 10 [5; 14] |
| Fayetville | USA | 0.55 [0.34; 0.77] | 9 [6; 13] | 0.48 [0.27; 0.7] | 8 [4; 12] |
| Gary | USA | 0.44 [0.26; 0.61] | 19 [11; 26] | 0.36 [0.18; 0.53] | 15 [8; 23] |

| City | Country | Total (Above 70 μg/m$^3$)* | | Above WHO Guideline (100 μg/m$^3$) | |
|---|---|---|---|---|---|
| | | Excess fraction (%, 95% CI) | Annual excess deaths (N, 95% CI) | Excess fraction (%, 95% CI) | Annual excess deaths (N, 95% CI) |
| Greensburg | USA | 0.34 [0.19; 0.47] | 14 [8; 20] | 0.26 [0.12; 0.4] | 11 [5; 16] |
| Grand Heaven | USA | 0.45 [0.26; 0.64] | 6 [3; 8] | 0.36 [0.17; 0.56] | 5 [2; 7] |
| Grand Junctio | USA | 0.31 [0.19; 0.43] | 3 [2; 4] | 0.18 [0.04; 0.31] | 2 [0; 3] |
| Grand Rapids | USA | 0.39 [0.24; 0.55] | 15 [9; 20] | 0.31 [0.16; 0.47] | 12 [6; 18] |
| Greensboro | USA | 0.6 [0.38; 0.83] | 19 [12; 26] | 0.53 [0.31; 0.76] | 17 [10; 24] |
| Gasinesville | USA | 0.28 [0.17; 0.38] | 4 [2; 5] | 0.2 [0.08; 0.31] | 3 [1; 4] |
| Gettysburg | USA | 0.43 [0.27; 0.6] | 4 [3; 6] | 0.34 [0.17; 0.51] | 3 [2; 5] |
| Holland | USA | 0.5 [0.31; 0.7] | 2 [1; 3] | 0.41 [0.22; 0.61] | 2 [1; 3] |
| Harrisburg | USA | 0.36 [0.22; 0.49] | 8 [5; 12] | 0.29 [0.16; 0.43] | 7 [4; 10] |
| Hartford | USA | 0.3 [0.18; 0.42] | 23 [14; 32] | 0.23 [0.1; 0.35] | 17 [8; 27] |
| Houston | USA | 0.27 [0.16; 0.37] | 47 [27; 65] | 0.22 [0.11; 0.33] | 38 [19; 58] |
| Indianapolis | USA | 0.52 [0.32; 0.72] | 37 [23; 51] | 0.44 [0.23; 0.64] | 31 [16; 46] |
| Iowa city | USA | 0.2 [0.12; 0.28] | 1 [1; 1] | 0.13 [0.05; 0.21] | 1 [0; 1] |
| Jacksonville | USA | 0.26 [0.16; 0.36] | 15 [9; 21] | 0.18 [0.07; 0.28] | 10 [4; 17] |
| Jersy city | USA | 0.25 [0.14; 0.34] | 12 [7; 17] | 0.2 [0.09; 0.31] | 10 [5; 15] |
| Kalamazoo | USA | 0.36 [0.2; 0.5] | 6 [4; 9] | 0.27 [0.12; 0.42] | 5 [2; 8] |
| kenosha | USA | 0.46 [0.28; 0.63] | 5 [3; 7] | 0.37 [0.17; 0.53] | 4 [2; 6] |
| Kansas | USA | 0.2 [0.12; 0.28] | 21 [13; 29] | 0.15 [0.07; 0.23] | 16 [7; 24] |
| Knoxville | USA | 0.41 [0.25; 0.56] | 16 [9; 22] | 0.33 [0.16; 0.48] | 13 [6; 18] |
| Lafayette LA | USA | 0.29 [0.18; 0.41] | 3 [2; 5] | 0.22 [0.1; 0.34] | 3 [1; 4] |
| Lake charles | USA | 0.25 [0.14; 0.34] | 4 [2; 5] | 0.18 [0.06; 0.27] | 3 [1; 4] |
| Lakeland | USA | 0.23 [0.14; 0.33] | 12 [7; 17] | 0.15 [0.06; 0.25] | 8 [3; 13] |
| Lancaster | USA | 0.41 [0.25; 0.58] | 16 [10; 22] | 0.35 [0.19; 0.52] | 13 [7; 20] |
| Louisville | USA | 0.33 [0.19; 0.46] | 22 [13; 31] | 0.27 [0.13; 0.4] | 18 [8; 27] |
| La Porte | USA | 0.5 [0.3; 0.7] | 5 [3; 7] | 0.41 [0.2; 0.62] | 4 [2; 6] |
| Los Angeles | USA | 0.41 [0.24; 0.57] | 242 [142; 335] | 0.36 [0.19; 0.52] | 211 [112; 307] |

| City | Country | Total (Above 70 μg/m³)* | | Above WHO Guideline (100 μg/m³) | |
|---|---|---|---|---|---|
| | | Excess fraction (%, 95% CI) | Annual excess deaths (N, 95% CI) | Excess fraction (%, 95% CI) | Annual excess deaths (N, 95% CI) |
| Las Vegas | USA | 0.34 [0.2; 0.47] | 29 [17; 41] | 0.26 [0.12; 0.39] | 22 [10; 34] |
| Little Rock | USA | 0.28 [0.16; 0.39] | 8 [5; 12] | 0.2 [0.09; 0.32] | 6 [3; 10] |
| Macon | USA | 0.53 [0.32; 0.76] | 9 [6; 13] | 0.45 [0.23; 0.68] | 8 [4; 12] |
| Mc Allen | USA | 0.13 [0.08; 0.18] | 4 [2; 5] | 0.07 [0.01; 0.13] | 2 [0; 4] |
| Middlesex | USA | 0.31 [0.18; 0.44] | 16 [10; 23] | 0.26 [0.14; 0.4] | 14 [7; 21] |
| Middletown | USA | 0.44 [0.27; 0.62] | 10 [6; 15] | 0.36 [0.2; 0.55] | 9 [5; 13] |
| Medford | USA | 0.32 [0.2; 0.45] | 5 [3; 7] | 0.22 [0.09; 0.36] | 4 [2; 6] |
| Madison IL | USA | 0.25 [0.15; 0.34] | 6 [3; 8] | 0.19 [0.09; 0.29] | 4 [2; 7] |
| Modesto | USA | 0.36 [0.23; 0.49] | 11 [7; 15] | 0.29 [0.16; 0.44] | 9 [5; 13] |
| Madison WI | USA | 0.32 [0.19; 0.45] | 7 [4; 10] | 0.23 [0.09; 0.37] | 5 [2; 8] |
| Miami | USA | 0.14 [0.09; 0.2] | 25 [16; 36] | 0.08 [0.02; 0.14] | 14 [3; 26] |
| Melbourn | USA | 0.22 [0.13; 0.3] | 10 [6; 13] | 0.13 [0.04; 0.22] | 6 [2; 10] |
| Milwauke | USA | 0.28 [0.17; 0.4] | 31 [19; 45] | 0.21 [0.09; 0.33] | 23 [10; 37] |
| Memphis | USA | 0.54 [0.34; 0.75] | 39 [24; 54] | 0.46 [0.25; 0.67] | 33 [18; 49] |
| Monmouth | USA | 0.46 [0.27; 0.65] | 52 [30; 73] | 0.39 [0.21; 0.58] | 43 [23; 65] |
| Montgomery | USA | 0.37 [0.22; 0.52] | 7 [4; 10] | 0.29 [0.14; 0.44] | 6 [3; 8] |
| Mobile | USA | 0.3 [0.18; 0.42] | 10 [6; 15] | 0.22 [0.09; 0.34] | 8 [3; 12] |
| Monroe | USA | 0.29 [0.18; 0.41] | 4 [2; 5] | 0.22 [0.1; 0.34] | 3 [1; 4] |
| Mercer | USA | 0.38 [0.23; 0.53] | 5 [3; 7] | 0.32 [0.17; 0.47] | 4 [2; 6] |
| Marlboro | USA | 0.46 [0.29; 0.64] | 18 [11; 25] | 0.4 [0.23; 0.59] | 16 [9; 23] |
| Muskegon | USA | 0.49 [0.3; 0.69] | 7 [4; 10] | 0.41 [0.21; 0.61] | 6 [3; 9] |
| Muncie | USA | 0.53 [0.31; 0.77] | 7 [4; 10] | 0.44 [0.22; 0.67] | 6 [3; 9] |
| Nashua | USA | 0.28 [0.17; 0.39] | 7 [4; 9] | 0.19 [0.08; 0.3] | 5 [2; 7] |
| Nassau | USA | 0.23 [0.14; 0.32] | 50 [30; 71] | 0.18 [0.08; 0.28] | 39 [18; 61] |
| Niles | USA | 0.56 [0.34; 0.79] | 8 [5; 12] | 0.46 [0.24; 0.69] | 7 [4; 10] |
| Nashville | USA | 0.21 [0.13; 0.29] | 10 [6; 14] | 0.15 [0.07; 0.24] | 7 [3; 11] |

| City | Country | Total (Above 70 µg/m³)* | | Above WHO Guideline (100 µg/m³) | |
|---|---|---|---|---|---|
| | | Excess fraction (%, 95% CI) | Annual excess deaths (N, 95% CI) | Excess fraction (%, 95% CI) | Annual excess deaths (N, 95% CI) |
| Newburgh | USA | 0.42 [0.26; 0.59] | 11 [6; 15] | 0.33 [0.16; 0.51] | 8 [4; 13] |
| Newhaven | USA | 0.35 [0.21; 0.49] | 27 [16; 37] | 0.27 [0.13; 0.41] | 20 [9; 31] |
| Newlond | USA | 0.49 [0.29; 0.68] | 9 [6; 13] | 0.39 [0.19; 0.59] | 7 [4; 11] |
| New Orleans | USA | 0.3 [0.18; 0.42] | 31 [19; 44] | 0.24 [0.12; 0.36] | 25 [13; 38] |
| Newark | USA | 0.26 [0.16; 0.36] | 27 [17; 38] | 0.21 [0.11; 0.31] | 22 [11; 33] |
| New York | USA | 0.16 [0.09; 0.22] | 103 [59; 141] | 0.12 [0.05; 0.19] | 81 [33; 121] |
| Ocala | USA | 0.25 [0.15; 0.35] | 9 [6; 13] | 0.17 [0.06; 0.27] | 6 [2; 10] |
| Oklahoma | USA | 0.31 [0.19; 0.44] | 17 [11; 25] | 0.24 [0.11; 0.37] | 13 [6; 21] |
| Oakland | USA | 0.08 [0.04; 0.1] | 12 [7; 16] | 0.03 [0; 0.06] | 5 [-1; 10] |
| Omaha | USA | 0.14 [0.09; 0.2] | 5 [3; 7] | 0.08 [0.02; 0.14] | 3 [1; 5] |
| Orlando | USA | 0.26 [0.16; 0.36] | 19 [12; 27] | 0.17 [0.07; 0.28] | 13 [5; 21] |
| Philadelphia | USA | 0.28 [0.17; 0.39] | 122 [73; 170] | 0.24 [0.12; 0.35] | 102 [53; 151] |
| Phoenix | USA | 0.36 [0.23; 0.5] | 66 [42; 92] | 0.28 [0.14; 0.43] | 52 [26; 80] |
| Palm beach | USA | 0.12 [0.07; 0.16] | 13 [8; 18] | 0.06 [0.01; 0.11] | 7 [1; 12] |
| Plymouth | USA | 0.26 [0.16; 0.35] | 10 [6; 13] | 0.19 [0.09; 0.29] | 7 [3; 11] |
| Pensacola | USA | 0.32 [0.21; 0.45] | 8 [5; 11] | 0.24 [0.11; 0.36] | 6 [3; 9] |
| Portland OR | USA | 0.15 [0.09; 0.2] | 15 [9; 20] | 0.09 [0.03; 0.15] | 9 [3; 15] |
| Port Arthur | USA | 0.26 [0.16; 0.36] | 6 [4; 9] | 0.2 [0.1; 0.3] | 5 [2; 7] |
| Portage | USA | 0.41 [0.25; 0.57] | 4 [2; 5] | 0.32 [0.17; 0.49] | 3 [2; 5] |
| Portland ME | USA | 0.25 [0.16; 0.35] | 6 [3; 8] | 0.17 [0.07; 0.28] | 4 [2; 6] |
| Providence | USA | 0.37 [0.24; 0.51] | 45 [29; 62] | 0.28 [0.14; 0.42] | 34 [17; 51] |
| Pittsburg | USA | 0.25 [0.15; 0.35] | 38 [23; 53] | 0.2 [0.1; 0.3] | 31 [16; 46] |
| Richmond | USA | 0.46 [0.28; 0.64] | 25 [16; 36] | 0.39 [0.21; 0.58] | 22 [12; 32] |
| Rochester | USA | 0.18 [0.11; 0.25] | 11 [7; 15] | 0.13 [0.06; 0.21] | 8 [4; 12] |
| Rockville | USA | 0.46 [0.28; 0.64] | 20 [13; 28] | 0.4 [0.22; 0.59] | 17 [10; 26] |
| Reading | USA | 0.34 [0.2; 0.47] | 12 [7; 16] | 0.28 [0.14; 0.41] | 10 [5; 14] |

| City | Country | Total (Above 70 µg/m$^3$)* | | Above WHO Guideline (100 µg/m$^3$) | |
|---|---|---|---|---|---|
| | | Excess fraction (%, 95% CI) | Annual excess deaths (N, 95% CI) | Excess fraction (%, 95% CI) | Annual excess deaths (N, 95% CI) |
| Reno | USA | 0.24 [0.15; 0.35] | 5 [3; 7] | 0.15 [0.05; 0.27] | 3 [1; 6] |
| Raleigh | USA | 0.48 [0.29; 0.66] | 13 [8; 18] | 0.41 [0.22; 0.6] | 11 [6; 17] |
| Riverside | USA | 0.6 [0.35; 0.85] | 124 [73; 175] | 0.55 [0.31; 0.8] | 113 [64; 166] |
| Sacramento | USA | 0.32 [0.2; 0.45] | 27 [16; 37] | 0.25 [0.12; 0.38] | 21 [10; 31] |
| Scranton | USA | 0.3 [0.18; 0.43] | 22 [13; 31] | 0.23 [0.1; 0.36] | 17 [7; 26] |
| San Diego | USA | 0.34 [0.2; 0.47] | 59 [35; 82] | 0.23 [0.09; 0.37] | 41 [16; 66] |
| San Francisco | USA | 0.02 [0.01; 0.03] | 3 [2; 3] | 0.01 [0; 0.02] | 1 [-1; 2] |
| Salt Lake | USA | 0.44 [0.26; 0.62] | 19 [11; 26] | 0.35 [0.18; 0.53] | 15 [8; 23] |
| San Jose | USA | 0.11 [0.07; 0.15] | 9 [6; 13] | 0.06 [0.01; 0.11] | 5 [1; 9] |
| San Antonio | USA | 0.28 [0.18; 0.38] | 25 [16; 34] | 0.2 [0.08; 0.31] | 17 [7; 27] |
| Spokane | USA | 0.31 [0.18; 0.45] | 10 [6; 15] | 0.18 [0.04; 0.32] | 6 [1; 11] |
| Springfield MA | USA | 0.24 [0.14; 0.33] | 11 [6; 15] | 0.17 [0.07; 0.27] | 8 [3; 12] |
| Springfied MO | USA | 0.26 [0.16; 0.37] | 5 [3; 8] | 0.18 [0.08; 0.28] | 4 [2; 6] |
| Spartanburg | USA | 0.43 [0.24; 0.59] | 10 [6; 14] | 0.36 [0.18; 0.54] | 8 [4; 13] |
| Sarasota | USA | 0.24 [0.14; 0.33] | 17 [10; 24] | 0.16 [0.05; 0.25] | 11 [4; 18] |
| Steubenville | USA | 0.26 [0.16; 0.38] | 3 [2; 4] | 0.21 [0.11; 0.32] | 2 [1; 3] |
| Saint Charles | USA | 0.45 [0.27; 0.62] | 6 [4; 8] | 0.38 [0.2; 0.55] | 5 [3; 7] |
| Stockton | USA | 0.25 [0.15; 0.35] | 10 [6; 14] | 0.19 [0.09; 0.29] | 7 [3; 11] |
| South bend | USA | 0.46 [0.28; 0.64] | 11 [6; 15] | 0.37 [0.18; 0.55] | 9 [4; 13] |
| St Louis | USA | 0.32 [0.2; 0.44] | 48 [29; 66] | 0.25 [0.12; 0.38] | 37 [18; 56] |
| Stamford | USA | 0.42 [0.26; 0.59] | 29 [18; 40] | 0.33 [0.17; 0.5] | 23 [11; 34] |
| St. Petersbur | USA | 0.22 [0.13; 0.31] | 17 [10; 24] | 0.15 [0.06; 0.24] | 11 [5; 18] |
| State College | USA | 0.44 [0.26; 0.61] | 4 [2; 5] | 0.35 [0.18; 0.53] | 3 [2; 5] |
| Seattle | USA | 0.12 [0.07; 0.16] | 13 [8; 17] | 0.07 [0.02; 0.12] | 7 [2; 13] |
| Tacoma | USA | 0.11 [0.07; 0.15] | 5 [3; 7] | 0.05 [0.01; 0.11] | 2 [0; 5] |
| Tampa | USA | 0.25 [0.16; 0.36] | 19 [12; 27] | 0.18 [0.08; 0.29] | 13 [6; 22] |

| City | Country | Total (Above 70 μg/m³)* | | Above WHO Guideline (100 μg/m³) | |
|---|---|---|---|---|---|
| | | Excess fraction (%, 95% CI) | Annual excess deaths (N, 95% CI) | Excess fraction (%, 95% CI) | Annual excess deaths (N, 95% CI) |
| Tucson | USA | 0.29 [0.18; 0.41] | 18 [11; 26] | 0.19 [0.07; 0.32] | 12 [5; 20] |
| Tallahassee | USA | 0.24 [0.15; 0.34] | 3 [2; 4] | 0.17 [0.06; 0.27] | 2 [1; 3] |
| Toledo | USA | 0.34 [0.19; 0.47] | 15 [9; 21] | 0.26 [0.11; 0.4] | 11 [5; 17] |
| Trenton | USA | 0.34 [0.21; 0.47] | 10 [6; 13] | 0.29 [0.15; 0.42] | 8 [4; 12] |
| Terra Haute | USA | 0.46 [0.28; 0.64] | 5 [3; 7] | 0.37 [0.19; 0.57] | 4 [2; 6] |
| Tulsa | USA | 0.34 [0.2; 0.48] | 16 [9; 22] | 0.27 [0.12; 0.4] | 12 [5; 18] |
| Visalia | USA | 0.6 [0.36; 0.85] | 14 [9; 20] | 0.56 [0.32; 0.8] | 13 [8; 19] |
| Vancouver | USA | 0.13 [0.08; 0.18] | 2 [1; 3] | 0.07 [0.01; 0.12] | 1 [0; 2] |
| Ventura | USA | 0.44 [0.27; 0.64] | 18 [11; 27] | 0.36 [0.18; 0.55] | 15 [8; 23] |
| Wichita | USA | 0.2 [0.12; 0.28] | 7 [4; 9] | 0.14 [0.06; 0.22] | 5 [2; 7] |
| Weber | USA | 0.62 [0.37; 0.85] | 7 [4; 10] | 0.53 [0.28; 0.77] | 6 [3; 9] |
| Wilmington | USA | 0.27 [0.16; 0.38] | 10 [6; 14] | 0.22 [0.11; 0.33] | 8 [4; 12] |
| Winston | USA | 0.53 [0.31; 0.74] | 13 [8; 18] | 0.45 [0.23; 0.66] | 11 [6; 17] |
| Worcester | USA | 0.31 [0.19; 0.44] | 20 [13; 28] | 0.23 [0.11; 0.36] | 15 [7; 23] |
| WDC | USA | 0.22 [0.13; 0.31] | 15 [9; 21] | 0.18 [0.09; 0.28] | 12 [6; 19] |
| Washington | USA | 0.37 [0.22; 0.52] | 9 [5; 12] | 0.31 [0.16; 0.45] | 7 [4; 11] |
| Youngstown | USA | 0.41 [0.24; 0.57] | 17 [10; 24] | 0.34 [0.18; 0.51] | 14 [7; 21] |
| York | USA | 0.38 [0.22; 0.52] | 11 [7; 16] | 0.32 [0.16; 0.46] | 9 [5; 14] |

**eTable 6.** Overall pooled estimates of ozone-mortality association (lag01) (95% confidence interval (CI)) expressed as relative risk (RR) per 10-µg/m$^3$ increase in ozone for each of the sensitivity analyses, except for the different approaches to control for temperature (reported in eFigure 3). Details on the analytical approach of each sub-analysis reported in eMethods 2.

| | N locations | RR [95% CI] |
|---|---|---|
| *Main analysis* | 406 | 1.0018 [1.0012; 1.0024] |
| Time control with 4 df | 406 | 1.0019 [1.0011; 1.0026] |
| Time control with 10 df | 406 | 1.0017 [1.0012; 1.0023] |
| Main analysis (restricted to $PM_{10}$ locations) | 228 | 1.0017 [1.0010; 1.0024] |
| Control for $PM_{10}$ (24-h average – linear lag01) | 228 | 1.0015 [1.0007; 1.0024] |
| Main analysis (restricted to $NO_2$ locations) | 269 | 1.0015 [1.0010; 1.0019] |
| Control for $NO_2$ (24-h average – linear lag01) | 269 | 1.0014 [1.0010; 1.0019] |
| Main analysis (restricted to $NO_2$ locations) | 89 | 1.0011 [0.9999; 1.0024] |
| Control for $NO_2$ (24-h average – linear lag01) | 89 | 1.0007 [0.9994; 1.002] |
| Main analysis (restricted to locations with rel humidity) | 317 | 1.0020 [1.0013; 1.0026] |
| Control for relative humidity (24-h average – linear lag0) | 317 | 1.0026 [1.0017; 1.0035] |
| Restriction to all-year series | 309 | 1.0018 [1.0012; 1.0024] |

*df: degrees of freedom. qAIC df7 (main model): 10376950. qAIC df4 (sensitivity analysis 1): 10378178. qAIC df10 (sensitivity analysis 2): 10389530.

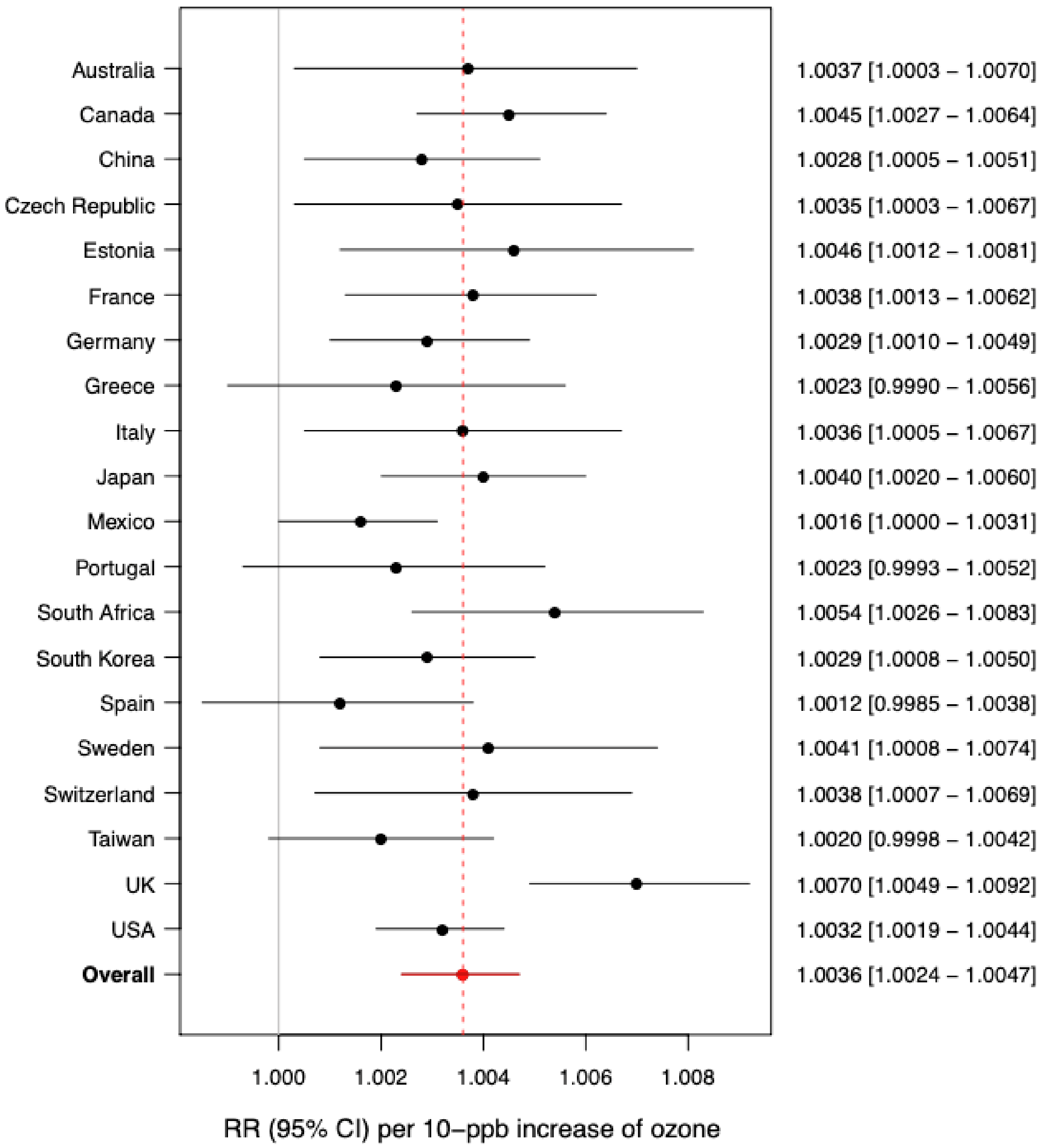


**eFigure 1.** Overall and country-specific short-term ozone-mortality association, expressed as relative risk (RR) per 10-ppb increase in ozone (maximum 8-hour average) (lag 01).

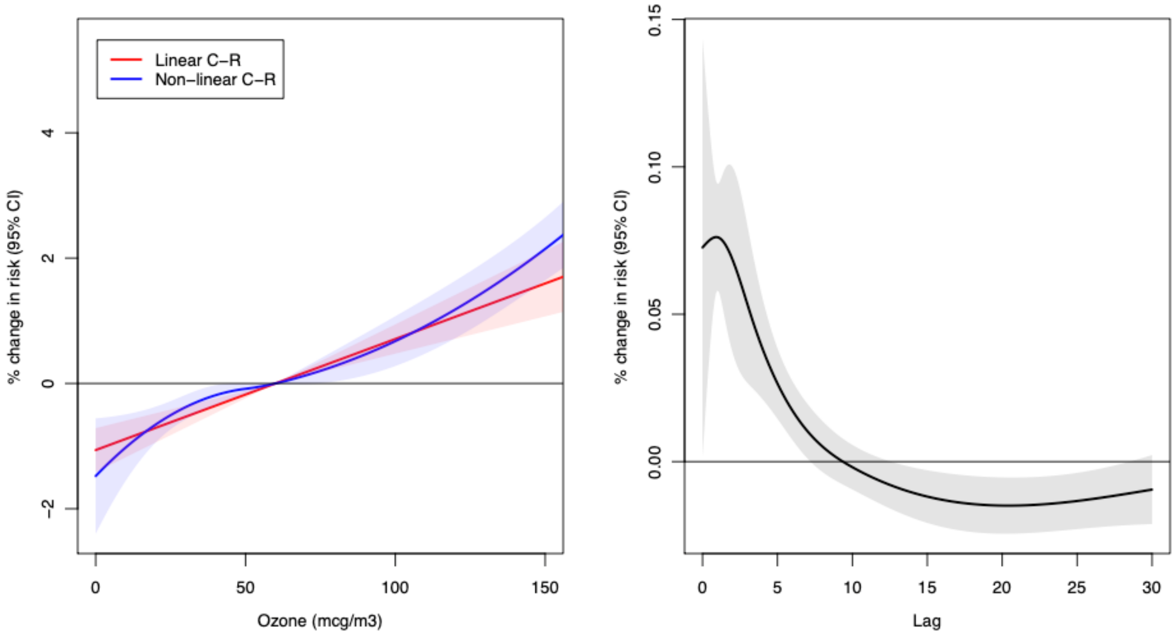


**eFigure 2.** Results from the additional analyses. Comparison of the average concentration-response (C-R) shapes using linear and non-linear functions (left) and lag-response association up to 30 days (right) per 10-unit increase in ozone. q-AIC linear: 10376950, Q-AIC non-linear: 10379020.

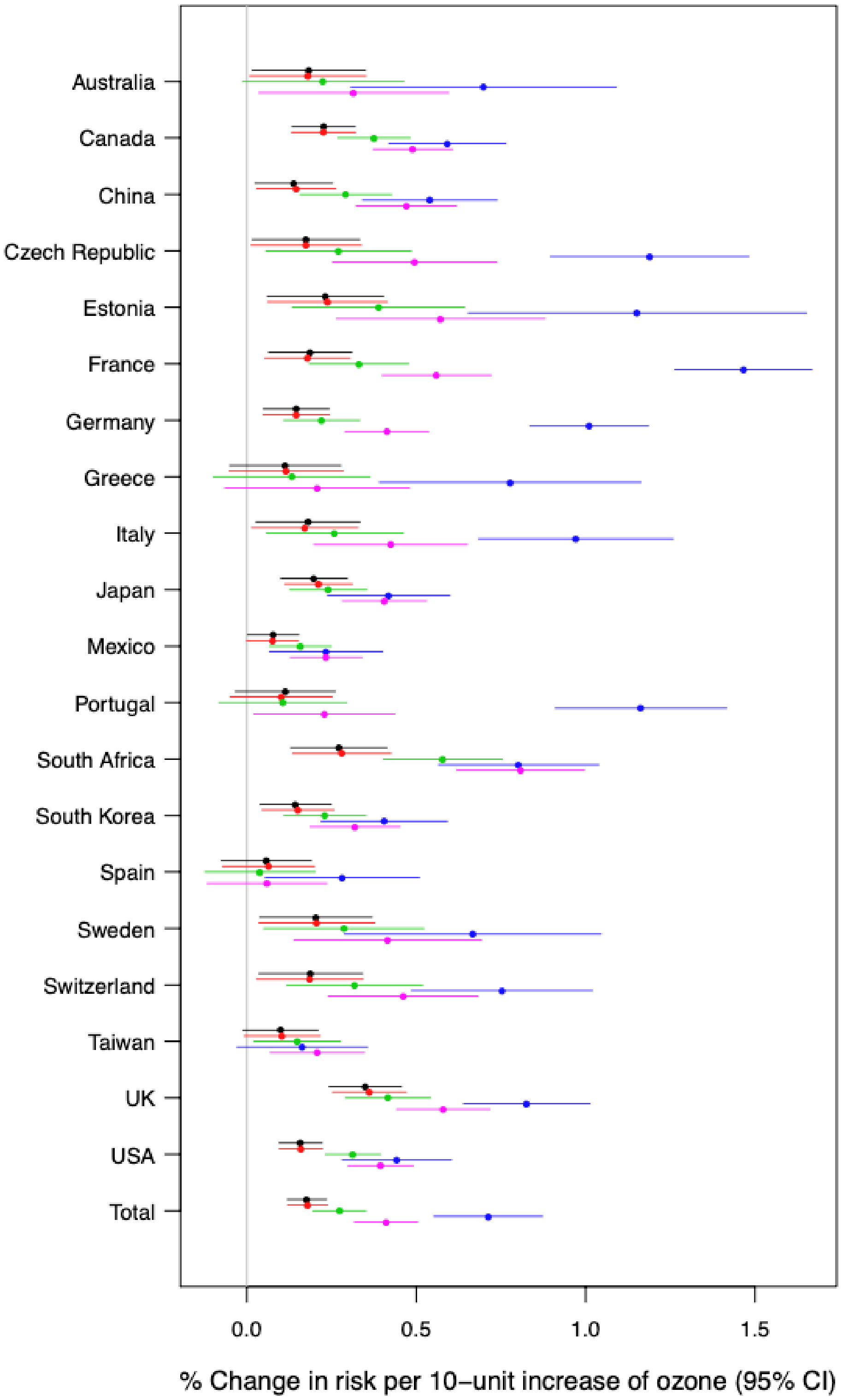


**eFigure 3.** Overall and country-specific short-term ozone-mortality association, expressed as relative risk (RR) per 10-unit increase in ozone, using different control for temperature (details on the modelling in eMethods2). (Black, main model (qAIC: 10376950); red, approach 1 (qAIC: 10377749); green, approach 2 (qAIC: 10380318); blue, approach 3 (qAIC: 10389132); pink, approach 4 (qAIC: 10387640)).
